# A High-Dimensional, Deep-Sequencing Study of Lung Adenocarcinoma in Female Never-Smokers

**DOI:** 10.1371/journal.pone.0055596

**Published:** 2013-02-06

**Authors:** Sang Cheol Kim, Yeonjoo Jung, Jinah Park, Sooyoung Cho, Chaehwa Seo, Jaesang Kim, Pora Kim, Jehwan Park, Jihae Seo, Jiwoong Kim, Seongjin Park, Insu Jang, Namshin Kim, Jin Ok Yang, Byungwook Lee, Kyoohyoung Rho, Yeonhwa Jung, Juhee Keum, Jinseon Lee, Jungho Han, Sangeun Kang, Sujin Bae, So-Jung Choi, Sujin Kim, Jong-Eun Lee, Wankyu Kim, Jhingook Kim, Sanghyuk Lee

**Affiliations:** 1 Korean Bioinformation Center (KOBIC), Korea Research Institute of Bioscience and Biotechnology, Daejeon, Korea; 2 Ewha Research Center for Systems Biology (ERCSB), Ewha Womans University, Seoul, Korea; 3 Division of Life and Pharmaceutical Sciences and the Center for Cell Signaling and Drug Discovery Research, Ewha Womans University, Seoul, Korea; 4 Samsung Biomedical Research Institute (SBRI) and Cancer Research Institute, Samsung Medical Center, Sungkyunkwan University School of Medicine, Seoul, Korea; 5 Department of Pathology, Samsung Medical Center, Sungkyunkwan University School of Medicine, Seoul, Korea; 6 DNA Link Inc., Seoul, Korea; 7 Department of Thoracic Surgery, Samsung Medical Center, Sungkyunkwan University School of Medicine, Seoul, Korea; Queen Elizabeth Hospital, Hong Kong

## Abstract

**Background:**

Deep sequencing techniques provide a remarkable opportunity for comprehensive understanding of tumorigenesis at the molecular level. As omics studies become popular, integrative approaches need to be developed to move from a simple cataloguing of mutations and changes in gene expression to dissecting the molecular nature of carcinogenesis at the systemic level and understanding the complex networks that lead to cancer development.

**Results:**

Here, we describe a high-throughput, multi-dimensional sequencing study of primary lung adenocarcinoma tumors and adjacent normal tissues of six Korean female never-smoker patients. Our data encompass results from exome-seq, RNA-seq, small RNA-seq, and MeDIP-seq. We identified and validated novel genetic aberrations, including 47 somatic mutations and 19 fusion transcripts. One of the fusions involves the *c-RET* gene, which was recently reported to form fusion genes that may function as drivers of carcinogenesis in lung cancer patients. We also characterized gene expression profiles, which we integrated with genomic aberrations and gene regulations into functional networks. The most prominent gene network module that emerged indicates that disturbances in G2/M transition and mitotic progression are causally linked to tumorigenesis in these patients. Also, results from the analysis strongly suggest that several novel microRNA-target interactions represent key regulatory elements of the gene network.

**Conclusions:**

Our study not only provides an overview of the alterations occurring in lung adenocarcinoma at multiple levels from genome to transcriptome and epigenome, but also offers a model for integrative genomics analysis and proposes potential target pathways for the control of lung adenocarcinoma.

## Introduction

Recent advances in DNA sequencing technology have revolutionized genomics and biomedical research, especially in the field of cancer research [1]. Various types of mutations as well as large scale chromosomal aberrations are being reported and cataloged, and the rate of data accumulation will likely accelerate for the foreseeable future. This should certainly apply to lung cancer which is currently the second most common cancer and the primary cause of mortality among cancer-related death in the United States [2].

The first complete sequence of a lung adenocarcinoma genome revealed about 50 000 single nucleotide variations in the tumor relative to normal lung [3]. This was followed by the sequencing study of a small-cell lung cancer genome which highlighted the role of tobacco carcinogens such as polycyclic aromatic hydrocarbons in shaping mutational patterns in lung cancers from smokers [4]. Transcriptome analysis of multiple lung adenocarcinoma patients using next-generation sequencing (NGS) recently showed the existence of a fusion gene containing the tyrosine kinase domain of the *c-RET* oncogene in 1%–2% of patients; this fusion leads to aberrant activation of *RET* kinase and is considered to be a new driver mutation of lung adenocarcinoma [5]. This finding was further confirmed through an independent study using a combination of targeted sequencing with an integrated molecular- and histopathology-based screening system [6]. Given that patients with *c-RET* fusions do not harbor mutations or fusions in *EGFR*, *KRAS or ALK* oncogenes, it is likely that c-*RET* fusion genes represent lung adenocarcinoma drivers and will lead to the definition of a new subclass of lung cancer [5].

Identifying mutations with high probabilities of being ‘drivers’, mutations that confer genes with oncogenic activity, is clearly a prototypical and certainly already a productive application of NGS, but the greater challenge is moving beyond the simple cataloging of mutations and establishing means for integrating diverse high-throughput data generated by NGS [7] to understand cancer at the multiple levels of gene networks and signaling pathways [8]. In this report, we describe a high-dimensional, high-throughput sequencing study of primary lung tumors and matched normal tissues isolated from 6 Korean female never-smoker patients with non-small cell lung cancer (NSCLC). This is the first multi-dimensional study of NSCLC that covers the exome-seq, RNA-seq, small RNA-seq, and methylated DNA immunoprecipitation-sequencing (MeDIP-seq). To complement the NGS data and obtain a full picture of sequence and structural variation, we also performed microarray-based gene expression profiling and array comparative genomic hybridization (array-CGH) study for DNA copy number variations (CNVs). Our study represents the simultaneous probing of the genome, transcriptome, and epigenome of biological samples revealing the full spectrum of cancer-associated alterations, including structural and genetic variations as well as changes in gene expression and epigenetic regulation. More importantly, we describe integrative analyses that entail the combination of the different types of omic data obtained in this study and that identify key regulators of NSCLC and elucidate relevant cellular processes at the systems level. Our findings show that the gene network modules that are highly relevant to the development of cancer, including those that govern progression through mitosis, are consistently disturbed in these NSCLC patients. We also report that multiple microRNAs are consistently inversely correlated with the predicted and validated target genes within these modules and throughout the whole network, indicating that microRNAs might be key regulatory agents of NSCLC development.

## Results

### Multi-omic Data Description

**Figure 1:**
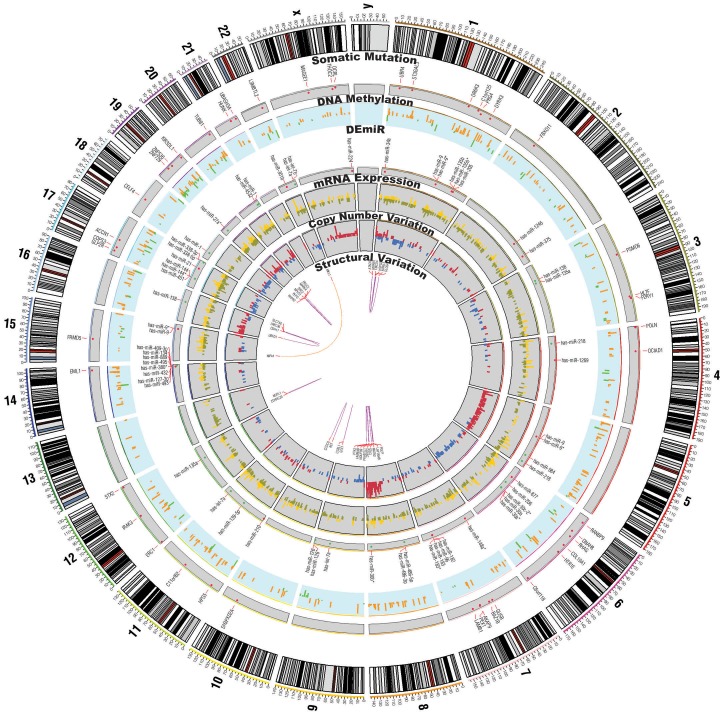
Circos plot of somatic mutations, copy number variations, transcriptome expression, and structural variations. From inside to out, structural variations (purple and orange), copy number variations (gain in dark red, loss in dark blue, mRNA expression (up in gold, down in olive), differentially expressed microRNAs (up in red, down in green), DNA methylation with sky-blue background (up in dark orange, down in chartreuse), somatic mutations with a gene symbols, and chromosomal cytobands.

To understand the genomic, transcriptomic and epigenomic changes in NSCLC, we performed high-throughput sequencing experiments for exome, transcriptome, and methylome on matched normal and tumor samples of 6 female non-smoker patients (see Figure S1 in File S8; data summary, experimental procedures are provided in the File S8; detailed sample/patient descriptions are provided in Table S1 in File S8 and File S1). CNV data were obtained from array-CGH assays. The genomic landscape of all NSCLC samples analyzed is visualized as a Circos plot of somatic mutations, transcriptome expression, CNVs, and structural variations_ENREF_6 (Figure 1; see Table S2 in File S8 for summary statistics of the exome data and Figure S2 in File S8 for Circos plots for individual patients) [9]. Experimental raw data and processed results are deposited in GEO (GSE37765) and SRA (SRA051952) databases. Raw additional data and information are also available at http://www.lungcancer.or.kr.

### NSCLC Somatic Mutations from Exome Sequencing Data

In our case, mutation calling by conventional programs such as *Varscan* (version 1.0) [10] did not show satisfactory performance, which was most likely due to the problem of normal cell contamination or heterogeneity of cancer cells. We therefore used the *JointSNVmix* program instead, to take advantage of the paired nature of samples (tumour and adjacent normal material) [11]. After validation by Sanger sequencing, we identified 47 somatic mutations that included 37 missense, 2 nonsense, and 7 silent mutations; there was also 1 mutation in the 3′ UTR (see Figure S3 in File S8). For several ambiguous cases, we subcloned PCR products and sequenced individual plasmid clones to confirm the mutation calls. Analyses of the validation process indicated that stringent criteria are required for the reliable prediction of somatic mutations if bulk clinical samples are used, as they were in our study. Cases with a predicted probability of over 0.999 often turned out to be false (45 positives and 55 negatives out of the 103 cases tested; PCR amplification failed in 3 cases). It should nevertheless be noted that some of these ‘false-positive’ somatic mutations may have occurred in a minority of tumor cells and are in fact positive, and newly discovered mutations in the future should be examined (e.g. for recurrence) with respect to all available raw data rather than just those confirmed by Sanger sequencing.

All confirmed mutations except for one were homozygous in the normal tissue. None of the somatic mutations identified in this study were identical to those reported in previous studies. In fact, none of the mutated genes isolated in this study except for *CELF4* (G86C/S29T in the COSMIC database; G86A/S29N in patient 3) have been reported to be mutated in other studies or in the COSMIC database. A complete list of mutations is provided in Table S3 in File S8, and a summary of gene functions is provided in File S6. It should be noted that several genes identified are known to have functions that might be relevant to cancer development: *BAZ1B* regulates the DNA-damage response by phosphorylating the histone H2A.X; *POLN* is a DNA polymerase that performs translesion synthesis in response to DNA damage; and *FBOX11*, a component of the Skp1-Cullin1-F-box (SCF) complex, promotes neddylation of p53 and inhibits its transcriptional activity.

### Differentially Expressed genes and Isoforms from RNA-Seq Data

We used the *Bowtie* and *NEUMA* applications for the mapping and quantification of RNA-Seq data, respectively [12], [13] (see Table S4 in File S8 for RNA-Seq data mapping summary). *NEUMA*, our in-house developed software (accessible at http://neuma.kobic.re.kr/), provides a highly accurate estimation of transcript abundance both at the gene and individual splice variant (isoform) levels using an algorithm that mimics the real-time PCR process.

Determining differentially expressed genes (DEGs) and differentially expressed isoforms (DEIs) from RNA-Seq data was performed using the *edgeR* program, which supports the analysis of paired samples. A rigorous filtering procedure based on false discovery rates, minimum applicable patient numbers, and gene expression levels was devised to select reliable sets of DEGs and DEIs (see File S8 for details). For the final result, we obtained 1459 DEGs (543 upregulated and 916 downregulated) and 1320 DEIs (460 upregulated and 860 downregulated) in tumors when compared with normal tissues (see Table S5 in File S8). Imposing additional requirement of a minimum two-fold change yielded 387 DEGs (98 upregulated and 289 downregulated in tumors). The detailed procedure of the RNA-Seq analysis is described in the File S8, and the list of DEGs is provided in File S2.

### Identification of Fusion Transcripts from RNA-Seq Data

**Figure 2:**
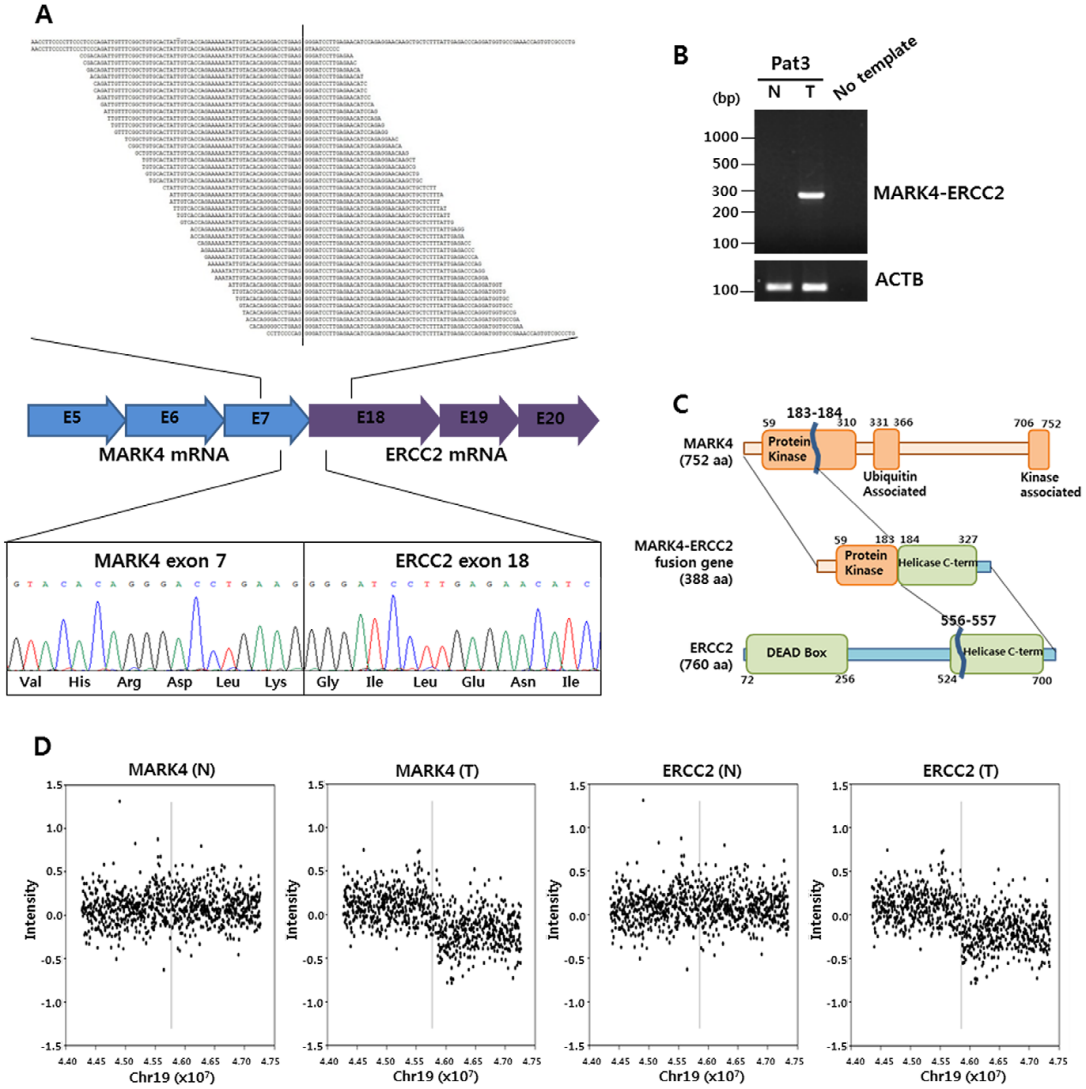
*MARK4-ERCC2* fusion transcript. (a) Allignment of sequence reads of fusion transcripts. The extent of the assembled fusion transcript appears at the top and reads are shows below it. The vertical line indicates the fusion point. The sequence to the left matches the 3′ end of exon 7 of *MARK4*, and the sequence to the right matches the 5′ end of exon 18 of *ERCC2*. (b) cDNA samples taken from tumor (T) and adjacent normal (N) tissue of patient 3 were used to confirm the presence of the *MARK4-ERCC2* fusion transcript by RT-PCR only in the tumor sample. ACTB was used as the internal control. (c) Schematic diagram of the predicted fusion protein along with domains having a defined function. The fusion protein is predicted to contain a part of the *MARK4* kinase domain and most of the C-terminal helicase domain of *ERCC2*. (d) Array-CGH profiles are shown for the *MARK4-ERCC2* intrachromosomal fusion. Note that the copy number variation is seen only in the tumor tissue but in not normal tissue. Vertical lines represent fusion points.

We used *FusionMap* [14] and an in-house developed application, *FusionScan*, to predict fusion transcripts from RNA-Seq data. These two programs require the fusion boundary to be found inside one of the sequence reads, even in the case of paired-end data. The likelihood of missing fusion transcripts due to this requirement should be minimal since our RNA-Seq data have a high sequencing coverage (32.7X on average after mapping) and long read length (78 bp on average). Given that the two applications produced an overwhelmingly large number of candidates, we further filtered the initial output candidates by manual inspection of alignment against the hypothetical fusion transcripts. All candidate transcripts were examined for coherency of the 5′–3′ direction between the two fusion partner transcripts and strict adherence to the established wild-type exon-intron boundaries. Experimental validation was carried out by RT-PCR and Sanger sequencing. In total, we confirmed 19 fusion transcripts from 5 out of 6 patients as summarily presented in Table 1, including the *MARK4-ERCC2* gene fusion shown in detail as an example (see Figure 2 and Table S6 in File S8).

**Table 1:** List of experimentally confirmed fusion genes.

Fusion Gene^1^	Head Gene	Head GeneLocation	Tail Gene	Tail GeneLocation	Sample	N/T^2^	Method^3^	CN^4^
*RHPN2-PEPD* ^*^	*RHPN2*	19q13.11	*PEPD*	19q13.11	P1T	T	B	✓
*SIRT2-NPHS1* ^*^	*SIRT2*	19q13	*NPHS1*	19q13.1	P1T	T	S	✓
*COX6C-LAPTM4B*	*COX6C*	8q22.2	*LAPTM4B*	8q22.1	P3T	T	B	✓
*MARK4-ERCC2* ^*^	*MARK4*	19q13.3	*ERCC2*	19q13.3	P3T	T	M	✓
*NDUFB9-PGCP*	*NDUFB9*	8q13.3	*PGCP*	8q22.2	P3T	T	B	✓
*SLC6A9-MORN1*	*SLC6A9*	1p33	*MORN1*	1p36.33	P3T	T	B	
*STK3-PTK2*	*STK3*	8q22.2	*PTK2*	8q24.3	P3T	T	B	✓
*PTK2-FAM84B*	*PTK2*	8q24.3	*FAM84B*	8q24.21	P3T	T	B	✓
*PKHD1L1-MATN2*	*PKHD1L1*	8q23	*MATN2*	8q22	P3T	T	B	✓
*MKL1-NIPA1*	*MKL1*	22q13	*NIPA1*	15q11.2	P4T	T	B	
*HSPG2-TMCO4*	*HSPG2*	1p36.1	*TMCO4*	1p36.13	P4T	T	B	
*NIPAL3-ATAD3B* ^*^	*NIPAL3*	1p36.12	*ATAD3B*	1p36.33	P4T	T	S	
*UBFD1-CDH11* ^*^	*UBFD1*	16p12	*CDH11*	16q21	P4T	T	S	
*SLC7A6-LRRC36*	*SLC7A6*	16q22.1	*LRRC36*	16q22.1	P4T	T	S	
*KDM6A-WSB1*	*KDM6A*	Xp11.2	*WSB1*	17q11.1	P6T	N,T	M	
*EIF1AX-PDE4DIP*	*EIF1AX*	Xp22.12	*PDE4DIP*	1q12	P6T	N,T	S	
*GRHL2-PTPN12* ^*^	*GRHL2*	8q22.3	*PTPN12*	7q11.23	P6T	N,T	S	
*CCDC6-RET* ^*^	*CCDC6*	10q21	*RET*	10q11.2	P8T	T	B	
*GLE1-CCBL1* ^*^	*GLE1*	9q34.11	*CCBL1*	9q34.11	P8T	T	B	

1 In-frame fusions are indicated with an asterisk.

2 N/T specifies the tissue type where fusion was detected (normal or tumor).

3 Method: S = FusionScan only, M = FusionMap only, B = both S and M.

4 CN: Supported by copy number variation (array-CGH) data.

Most of the validated fusion transcripts were intra-chromosomal (15 out of 19) and presented tumor-specific events (16 out of 19; Table 1); the others were found in both tumor and normal cells. This finding implies that at least a subset of fusion transcripts likely arose from intra-chromosomal events such as copy number changes specific to cancer cells. Indeed, the fusion case of *MARK4-ERCC2* showed the tumor-specific CNVs at the fusion points in the array-CGH data (Figure 2D). In-depth array-CGH analyses revealed 7 additional fusion events with strong association with DNA copy number changes (Table 1). Two fusions (*RHPN2-PEPD* and *SIRT2-NPHS1*) were observed in chromosome 19 of patient 1, and five cases were observed in chromosome 8 of patient 3 (*PTK2-FAM84B, COX6C-LAPTM4B, STK3-PTK2, PKHD1L1-MATN2, NDUFB9-PGCP*). Patient 3 harbored 7 fusion events in total two of which involved *PTK2*, also known as Focal Adhesion Kinase, a kinase with multiple functions including regulation of cell locomotion, survival and mitogen response [15]. In one patient (#6), all 3 fusions were detected in both normal and tumor samples, which suggests that these fusions are likely germline mutations. Of the 19 fusion events, 8 yielded in-frame gene fusions that potentially created proteins with novel functions.

Several of the gene fusion events could have an impact on cancer development and could potentially be driver mutations. For example, *ERCC2* is involved in transcription-coupled DNA repair, and the tyrosine phosphatase *PTPN12* is known to dephosphorylate and thereby inactivate the proto-oncogene c-*ABL* [16]. Perhaps most notably, one of the fusions involves *CCDC6* and c-*RET* kinase, which is seen frequently in papillary thyroid carcinoma [17]. Preliminary structural analysis shows that the c-*RET* kinase domain is intact, raising the strong possibility that this fusion is a so-called ‘driver’ mutation of NSCLC. Recent publications have reported fusion events of c-*RET* kinase with *KIF5B* as well as with *CCDC6* [5], [6], [18]. Finally, none of the identified fusions were found recurrently in our 6 patients, suggesting that a larger number of patients must be examined before the full significance of these fusion events can be evaluated.

### Functional Interpretation of Somatic Mutations, DEGs, and Fusion Events

Although most of the somatic mutations and gene fusions are probably ‘passenger’ mutations, we cannot rule any of them out *a priori* as drivers of carcinogenesis. To facilitate the process of isolating functional DEGs and significant mutations, we performed a gene set analysis (GSA) and network analysis for 1536 genes, including the 47 genes that we found to have somatic mutations, the 37 genes involved in fusion, and the 1459 DEGs, some of which belong to more than one category.

A GSA, which tests the statistical enrichment or depletion of specific annotation terms, provides a comprehensive functional summary for genes in the list. We used the Ingenuity Pathway Analysis (IPA) software, which uses a database of evidence manually compiled from the literature. The most enriched term in the diseases and disorders category was cancer (p value = 2.13E-42), which supports the validity of our gene set. Other relevant terms in the molecular and cellular functions category included cellular growth and proliferation (p value = 1.71E-17) and cell death (p value = 1.97E-17). The IPA results are presented in Figure S9 in File S8. Gene ontology (GO) analysis produced similar results to IPA, albeit in a less comprehensive manner (data not shown).

We sought to gain further mechanistic and functional insights about the genes of interest using a network-based analysis. Protein-protein interaction data from the MIMI database was superimposed onto our gene list. The overall network thus obtained (Figure S10 in File S8) is complex but reveals a number of interesting interactions that may be connected to tumorigenesis. To identify network modules of coherent function, we used the *MCODE* application to find densely connected network components [19]. We found 8 network modules consisting of 66 genes in total (Figure S11 in File S8). Most genes in each network module showed expression changes in the same direction, suggesting a coherent and coordinated function in carcinogenesis as a gene network module. The largest network module features genes involved in mitotic cell-cycle regulation. In fact, this network module contains several key genes such as *AURKB, PLK1, CCNE1, CCNB2, CHEK1*, and *PKMYT1*, which are involved in the G2/M transition and/or M-phase progression [20], [21], [22]. All the genes within the cell-cycle regulation module were significantly upregulated (see below).

### MicroRNA Analysis from Small RNA-Seq Data

Multiple studies have demonstrated that microRNAs could serve as viable tumor biomarkers and potential therapeutic targets or tools [23]. The computational pipeline for analyzing small RNA-Seq data (i.e., mapping, normalization, quantification, and identification of differentially expressed microRNAs) is illustrated in Figure S4 in File S8. On average, 70% and 76% of the total reads from small RNA-seq experiments were identified and mapped as microRNAs for normal and tumor tissues, respectively (see Table S7 in File S8).

Differentially expressed microRNAs (DEmiRs) were obtained using a process similar to that used to obtain DEGs. We limited the analysis to a subset of microRNAs belonging to the upper 25% in expression levels in at least one of the 12 samples. Other filtering conditions, such as fold change, were maintained. In total, we obtained 40 DEmiRs (23 up- and 17 downregulated in tumors compared to adjacent normal material; Table S8 in File S8 and File S3).

An inverse correlation in expression levels within a validated or predicted microRNA-mRNA pair provides strong evidence for an extant microRNA-target relationship in the biological context under examination. A search for inverse correlations between DEmiRs and DEGs yielded 151 relations (14 validated and 137 predicted) with a Pearson correlation cutoff of −0.5 and a p-value cutoff of 0.05 (see Table S9 and Figure S6 in File S8). Expanding the search scope beyond DEmiRs to identify other microRNAs of potential functional importance, we found 13 additional microRNAs with at least a two-fold change in expression between normal and tumor samples involved in 53 validated inverse correlations with DEGs. We did not use the predicted targets in this case, in order to avoid the inclusion of false positives. Overall, we identified 40 DEmiRs and 13 additional microRNAs that may play important roles in lung cancer development.

**Figure 3:**
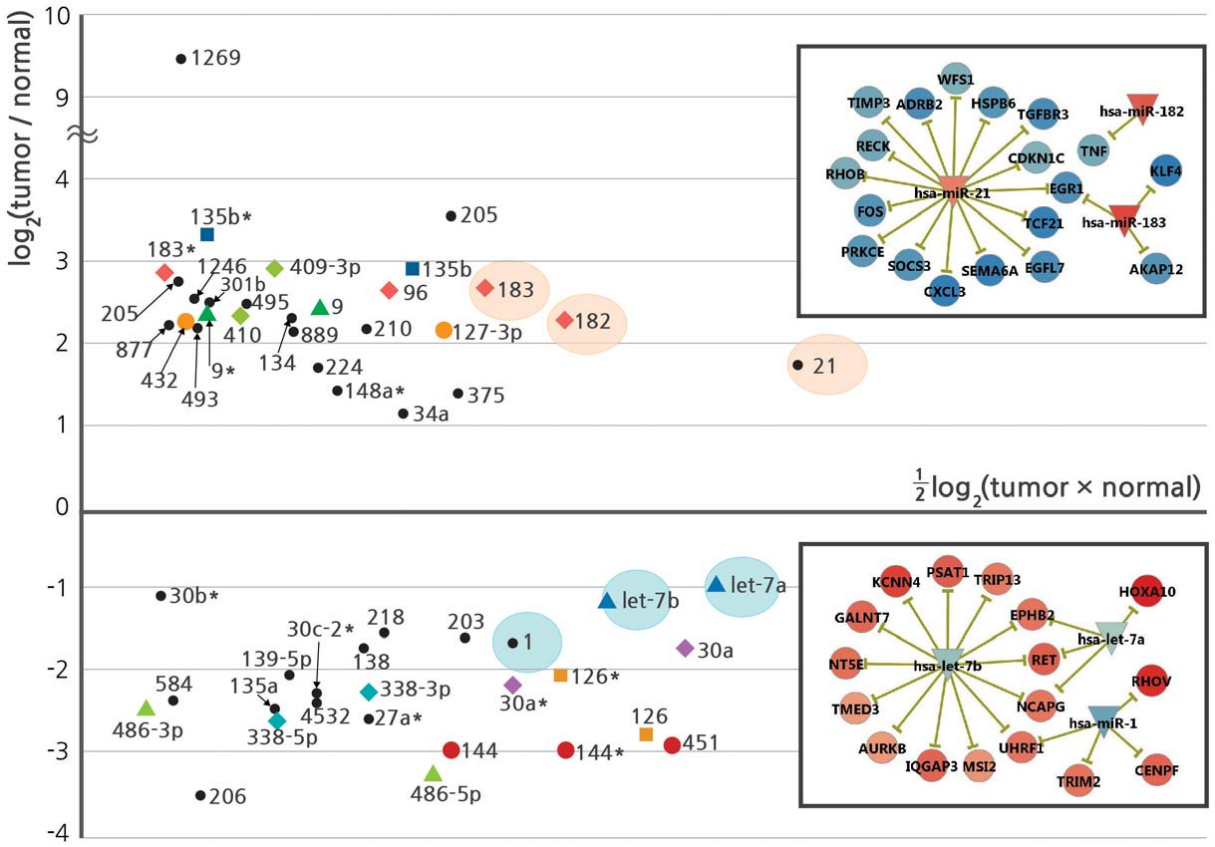
Differential expression of microRNAs. Fold change versus expression level is shown in the MA-plot of DEmiRs and anti-correlated microRNAs. MicroRNAs from the same genomic locus are shown with the same color and symbol (e.g., 96, 182, 183). MicroRNAs inversely correlated with DEGs are indicated with a black circle. Fold changes in log_2_ (tumor/normal) and expression magnitude in ½log_2_ (tumor × normal) are the average values over six patients. Inset figures show subsets of microRNA-centric relationships with targets potentially involved in carcinogenesis. Relevant microRNAs are indicated by background orange and blue ovals within the plot. Only the validated targets are shown for simplicity. Changes in expression levels are indicated via node color.

The MA-plot (logConcentration vs. logFoldChange) shows abundance and changes in expression and is thus an effective method for assessing the significance of potential biomarkers (see Figure 3 for the MA-plot of 40 DEmiRs and 13 additional microRNAs). Notably, the DEmiRs from a single genomic locus of chr7q32.2 (miR-96, miR-182, miR-183, and miR-183*) found upregulated in our study have also been previously reported as potential biomarkers for NSCLC [24]. By contrast, miR-144, miR-144* and miR-451 (all from chr17q11.2 locus) form a cluster of downregulated DEmiRs. Given that microRNAs derived from a polycistronic transcript often target the same set of genes, the change in expression of these microRNAs may have particularly strong effects. Among the 13 additional ‘non-DEmiR’ microRNAs, miR-21 (3.32 fold increase) and let-7b (2.20 fold decrease) deserve special attention. The miR-21 and let-7 families have previously been reported as oncomirs and tumor suppressors, respectively [25], [26]. Although neither family satisfied our stringent criteria for DEmiRs, their expression levels were among the highest, and their fold changes were statistically significant.

### Analysis of CNV and DNA Methylation Data

Copy number data from Agilent 1M CNV microarrays were analyzed using the within-slide lowess normalization and the circular binary segmentation (*CBS*) method [27]. We detected statistically significant somatic copy number alterations in tumor with the threshold of CNV change beyond log_2_ (tumor/normal) = ±0.3 (corresponding to the range outside 1.62–2.46 copies). We found multiple significant copy number gains in chromosomes 1, 5, 8, 16, 19, 20, and X, and copy number losses in chromosomes 1, 6, 9, 16, 17, 18, 19, and 22 (see Figure S7 and S8 in File S8 for CNV plots; Table S10 in File S8 for statistics). The genomic loci of copy number gains or losses in at least 3 patients included 8q22–24, 16p13, and 20q13 for gains and 1p12–13 and 9p21 for losses (File S4).

Comparison with previous studies (Table S11 in File S8) showed that our data agreed most closely with the Job *et al.* data that profiled 60 adenocarcinoma samples of never-smokers [28]. The genomic loci of 5p, 8q24, and 20q13 were repeatedly detected for copy number gains, and 9p21 was consistently detected for loss in copy numbers. Other markers may be never-smoker-female specific, which need to be validated with additional patient samples. Other chromosome-level events require further examination down the road. For example, the copy number gain in the large chr8q region, which has already been reported as a smoking-related biomarker for NSCLC patients [29], was detected in three of our six patients. Preliminary analysis indicated that many genes within this region were overexpressed in tumor samples (data not shown) and were involved in gene fusion in one of the patients (see Table 1).

DNA methylation patterns represent a potentially valuable biomarker in various types of cancer. We analyzed the DNA methylation data from MeDIP-seq using the *Eland* (version 2) and *edgeR* programs (see Table S14 in File S8 for mapping statistics; details in File S8). With conservative filtering options, we identified 558 differentially methylated regions (DMRs), almost 75% of which were in the promoter or 5′ UTR regions. The list of DMRs with genome annotation (from the UCSC genome browser database for hg19) is provided in File S5.

Copy number variations and DNA methylation are important factors in regulating gene expression. We investigated the correlation between CNV and mRNA expression in a similar manner to that performed in the microRNAs-target correlation study and obtained 107 positive correlations with a correlation cutoff of 0.5 (Table S12 in File S8). Highly correlated genes include several genes involved in gene fusion such as *STK3, NDUFB9, COX6C, FAM84B*, and *PTK2*. The inverse correlation analysis between mRNA expression and DNA methylation in promoter regions yielded 32 relations whose correlation coefficients were smaller than −0.3 (Table S13 in File S8).

### Pathway Modeling with Network Modules and microRNAs

**Figure 4:**
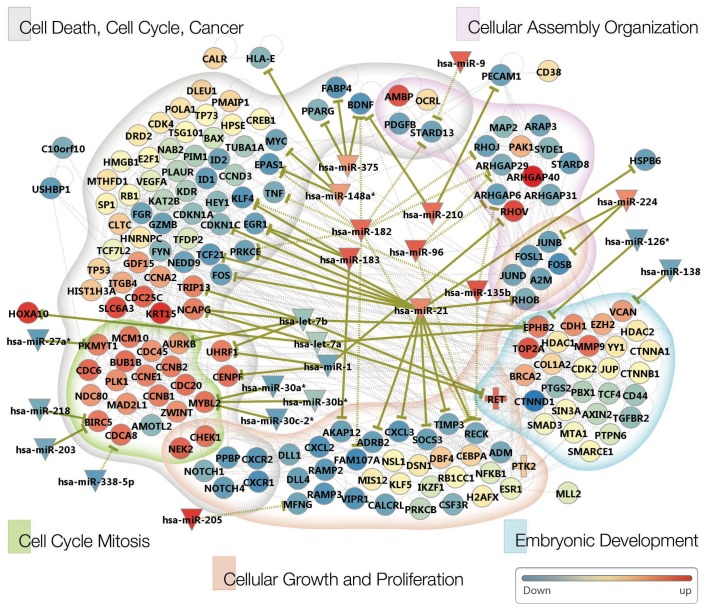
NSCLC pathway modeling for female never-smokers. The pathway information was obtained from an Ingenuity Pathway Analysis (IPA) using the 66 network module genes as an input list. The resulting genes were grouped into five functional categories as suggested by IPA. Validated and predicted microRNA-target relations are shown in solid and dotted lines, respectively. Changes in expression levels are indicated via node color (red for up-regulation and blue for down-regulation). For *c-RET* and *PTK2*, the+symbol was used to indicate that they are involved in gene fusion event.

Several validated and predicted target genes of anti-correlated microRNAs were DEGs that were found in gene network modules using the *MCODE* clustering analysis described above. To obtain a more comprehensive picture of the regulatory networks, we integrated the 66 genes from *MCODE* clustering and microRNA-target relations using the IPA systems knowledgebase. The resulting gene network now incorporates the relevant microRNAs and additional genes interacting with the 66 DEGs (Figure 4). Genes were clustered initially into broad functional categories, and the inverse correlations with microRNAs were superimposed. Consistent with the initial clustering analysis, the cell-cycle cluster labeled as “cell death, cell cycle, cancer” formed the most comprehensive cluster. Furthermore, the sub-clustering analysis shows that the mitosis module, subsumed within the cell-cycle cluster, formed the most tightly organized gene network model (Figure 4).

The integrated network presented in this study enables the examination of gene- and microRNA-expression changes in combination with the interactions among them. Several microRNA-target relationships relevant to tumorigenesis are readily observed within this network. As described above, let-7b shows multiple functionally significant and inverse correlative interactions with genes within the network. The expression of two genes whose overexpression can drive tumorigenesis, *AURKB* and *c-RET*, is inversely correlated to the reduced expression of let-7b microRNA. *AURKB* (aurora kinase B) is the catalytic component of the chromosomal passenger complex that is responsible for chromatin condensation, bipolar spindle formation and attachment of chromatin to the bipolar spindle [21]. *AURKB* is also a DEG and a member of the network module described above whose members regulate the progression through the M phase. The *c-RET* receptor tyrosine kinase [17] has been found as a fusion protein in which the kinase domain remains intact. An overexpression of this fusion gene is therefore expected to have oncogenic activity, as recent studies have consistently suggested^4^. Two other cell cycle-related genes, *NCAPG* and *UHRF1*, are targeted by let-7b. *NCAPG*, the subunit G of non-SMC condensin I complex, which is required for conversion of interphase chromatin into condensed mitotic chromatin [30], is targeted by both let-7a and let-7b, exemplifying the regulation of a gene by multiple members of a microRNA cluster. *UHRF1*, a RING-finger type E3 ubiquitin ligase, which plays a major role in G1/S transition [31], is another target of let-7b. *UHRF1* is also targeted by another microRNA, miR-1, which also regulates *CENPF*, a protein involved in kinetochore formation and chromosomal segregation. Whether the convergence of regulatory inputs into the genes from multiple microRNAs, as exemplified above, will lead to synergistic effects in the regulation of the mRNA level and/or in carcinogenesis remains an interesting question.

One of the most notable targets of the oncomir miR-21 is *RECK*, a membrane-anchored glycoprotein that inhibits matrix metalloproteinase-9. *RECK*, which is known to be strongly downregulated in multiple tumors and in cell lines transformed by oncogenes [32], is a predicted and inverse correlated target of several microRNAs (miR-96, miR-182, and miR-135b) within our network (Figure 4, Figure S6 in File S8). miR-96 and miR-182, which are DEmiRs from a single cluster, along with miR-183, have been proposed as *RECK* inhibitors, again exemplifying co-targeting by members of a single polycistronic cluster. Another microRNA-target relationship of significance involves *MYBL2*. This proto-oncogene produces the B-myb transcription factor, a well-known transcription factor with critical functions during G1/S and G2/M transitions. It is a validated target of miR-30a*, miR30b* and 30C-2*, all of which are downregulated in tumors consistent with upregulation of *MYBL2*.

## Conclusions

In this study, we sought to define the nature of pathology at the molecular and systemic level within tumor cells of NSCLC female never-smoker patients, a group with few mutations known. Never-smokers account for 20% of men and 50% of all women with lung cancer and likely represent an etiologically distinct group from smokers [33]. Activating mutations of the *EGFR* tyrosine kinase are found at a far higher frequency among non-smokers, and subsequently a correspondingly higher efficacy is seen with EGFR tyrosine kinase inhibitors such as gefitinib and erlotinib in these patients [34], [35]. The Tumor Sequencing Project (TSP), which used a conventional Sanger sequencing technique to examine the coding exons of 623 candidate cancer genes in 188 lung adenocarcinomas, revealed that smokers suffer mutations at rates 5- to 10-times higher than never-smokers [36]. Given that most somatic mutations are expected to be “passengers,” the smaller number of mutations among never-smoker patients offers the possibility of more effectively isolating driver mutations.

We have identified and validated multiple point mutations and gene fusion events from 6 patients. It should be noted that these patients were negative mutations in *KRAS* and *EGFR* genes and did not sustain a fusion between *EML4* and *ALK* which are the well-known transforming events of NSCLC. In fact, comparisons with other gene expression profiling studies of NSCLC cases indicate that the patient group tested in this study has acquired patterns in gene expression distinct from NSCLC cases with *KRAS*, *EGFR* or *ALK* mutations (File S7). A telling observation from our study was that none of the mutations or fusion genes was found in more than one patient. This strongly implies that for the large proportion of NSCLC cases negative for the established ‘driver’ mutations, the diversity or heterogeneity of mutations will likely remain a challenge for isolating biomarkers or drivers even in never-smokers. One of the most important goals for the future should be to expand the number of patient samples for NGS studies. This is critical for determining the recurrence and thereby the significance of each of the genetic aberrations.

Other than the *CCDC6*-*RET* fusion, which is expected to generate an oncogenic driver, it is difficult in most cases to predict the function of a given mutation (i.e. gain in oncogenic activity or a loss of tumor suppressor activity, or neither). This would be the case even when some of the information is available for the gene structure and function, unless an extensive characterization of the gene activity is performed *in vitro* and *in vivo*. Although such an analysis is obviously necessary, a different tactic based on integration of multiple omic data that investigates the interplay among genetic aberrations, transcripts, and regulatory factors in tumor cells can provide valuable information, as demonstrated in this study. Most importantly, we propose that the key cellular malfunction during tumorigenesis in our sampled population occurs in the control of the M-phase progression and that a set of specific microRNAs may be a source of viable biomarkers and functionally significant regulators of tumorigenesis. This will however require examination of individual genes in the context of lung cancer development as most of the genes promoting M-phase progression appear to up-regulated in diverse types of cancer as well according to our analysis on gene expression pattern using the GENT database (http://medical-genome.kribb.re.kr/GENT/; data not shown).

In summary, integrative data analyses, such as those performed in this study, may be the only practically viable method for handling the anticipated volume of data from NGS studies. As more high-throughput data of multiple types from additional tumor samples are combined with reference datasets and gene networks, such as those reported here, the predictive power of integrative analyses should become more evident.

## Supporting Information

File S1 **Complete pathological reports of 6 patients.**
(PDF)

File S2 **List of differentially expressed genes and isoforms (DEGs and DEIs).**
(XLS)

File S3 **MicroRNA expression plot for tumor and normal tissues.**
(XLS)

File S4 **List of CNV genes in at least 3 out of 6 patients.**
(XLS)

File S5 **List of DMR genes.**
(XLS)

File S6 **Gene lists with brief summaries for somatic mutations and network modules.**
(PDF)

File S7 **Comparison with public expression data on NSCLC.**
(PDF)

File S8 **Supplemental information; Supplemental materials, methods, figures, tables and references.**
(PDF)
