# Supplementary material for: A High-Dimensional, Deep-Sequencing Study of Lung Adenocarcinoma in Female Never-Smokers
Source: PLoS One. 2013 Feb 6;8(2):e55596. doi: 10.1371/journal.pone.0055596 (PMC3566005; doi:10.1371/journal.pone.0055596)
Supplement: File S1 — Complete pathological reports of 6 patients. (PDF) [file pone.0055596.s001.pdf]

Supplemental File 1. Complete pathological reports of 6 patients

PAT 1

Lung, left lower lobe, lobectomy:

Adenocarcinoma, moderately differentiated, mixed papillary with micropapillary and acinar type, lateral basal segment:

1. Size: 4x4 cm
2. Vascular invasion (arteriolar or venous): not identified
3. Lymphatic invasion: present
4. Perineural invasion: not identified
5. Margins Bronchial: 4 cm
6. Pleural/extrapleural: Tumor invades to visceral pleural surface (PL2)
7. Regional lymph nodes included in main specimen (N1)
    a. Total number examined: 3
    b. Number involved by tumor: 0
8. Separately submitted N1 or N2 lymph nodes
    a. Total number examined: 8
    b. Number involved by tumor: 0
       (0/8: "hilar", 0/2; "interlobar fissure 2", 0/3; "subcarina", 0/1; "L9", 0/1; "5", 0/1)
9. Additional pathologic findings: Background lung; pseudoangiomatous change, pleura

PAT 3

Lung, left lower lobe, lobectomy:

Mucinous adenocarcinoma, moderately differentiated, superior segment:

1. Size: 3.2x3 cm
2. Vascular invasion (arteriolar or venous): not identified
3. Lymphatic invasion: present
4. Perineural invasion: not identified
5. Margins Bronchial: 1 cm
6. Pleural/extrapleural
   Tumor superficially invading in the pleural connective tissue,
   but not beyond the elastic layer of the visceral pleura (PL0)
7. Micropapillary pattern, Central scar
8. Regional lymph nodes included in main specimen (N1)
    a. Total number examined: 6
    b. Number involved by tumor: 0
9. Separately submitted N1 or N2 lymph nodes
    a. Total number examined: 7
    b. Number involved by tumor: 0
       (0/7: "L4", 0/1; "5", 0/1; "7", 0/1; L10", 0/2; "L11", 0/2)
10. Additional pathologic findings: Background lung; Bullae

PAT 4

Lung, right lower lobe, lobectomy:

Adenocarcinoma, moderately differentiated, papillary,acinar, solid and focal micropapillary pattern, lateral basal segment:

1. Size: 5.5x5.0x5.0 cm
2. Vascular invasion (venous): present
3. Lymphatic invasion: present
4. Perineural invasion: not identified
5. Margins Bronchial 2.3 cm
6. Pleural/extrapleural
   Tumor invades to visceral pleural surface (PL2)
7. Regional lymph nodes included in main specimen (N1)
   a. Total number examined: 1
8. Number involved by tumor: 0
9. Separately submitted N1 or N2 lymph nodes
   a. Total number examined: 8
   b. Number involved by tumor: 4
      (4/8: interlobar fissure", 1/1; "RLLobar bronchus", 1/1;
      "7", 2/2; "R2", 0/3; "R4", 0/0; "R9", 0/1)
10. Additional pathologic findings: Background lung: unremarkable

PAT 5

Lung, left lower lobe and left upper lobe, left lower lobectomy and left upper lobe lingular segmentectomy:
Adenocarcinoma, poorly differentiated, mixed, solid and acinar patterns, anteromedial basal segment of left lower segment:

1. Size: 5.5x4x3.5 cm
2. Vascular invasion: not identified
3. Lymphatic invasion: present
4. Perineural invasion: not identified
5. Margins Bronchial: 0.5 cm
6. Pleural/extrapleural
   Tumor located within the lung parenchyma (PL0)
7. Others
   a. Focal micropapillary pattern
   b. Focal necrosis
8. Regional lymph nodes included in main specimen (N1)
   a. Total number examined: 4
   b. Number involved by tumor: 0
9. Separately submitted N1 or N2 lymph nodes
   a. Total number examined: 21
   b. Number involved by tumor: 1
   c. Size of the largest metastasis: 0.9
   d. Extracapsular extension: absent
      (1/21: "interlobar fissure", 0/1; "hilar", 1/1; "left pleural LN", 0/0; "5", 0/1;
      1. "7", 0/1; "L9", 0/11; "L11", 0/6)
10. Additional pathologic findings:
    Background lung; pleural fibrosis
    No evidence of malignancy, left upper lobe lingular segment

PAT 6

Lung, right upper lobe, video assisted thoracotomy's wedge resection:
Adenocarcinoma, well differentiated, mixed acinar and non-mucinous BAC pattern:

1. Size: 1.5x1 cm (invasion component, 0.9 cm)
2. Negative resection margins
3. Lymphatic invasion: not identified
4. Perineural invasion: not identified
5. Pleural/extrapleural
   intraparenchymal
6. Regional lymph nodes included in main specimen (N1)
   a. Total number examined: 3
   b. Number involved by tumor: 0
7. Separately submitted N1 or N2 lymph nodes
   a. Total number examined: 16
   b. Number involved by tumor: 0
      (0/16: "4", 0/3; "7", 0/3; "2", 0/3; "L10", 0/4; "L11", 0/3)
8. Additional pathologic findings: Background lung; unremarkable

PAT 8

Lung, left lower lobe, lobectomy:
Adenocarcinoma, moderately differentiated, mixed acinar and micropapillary pattern, with mucin producing, anterior segment:

1. Size: 2.5x2.5x2.0 cm
2. Vascular invasion (arteriolar or venous) : not identified
3. Lymphatic invasion: present
4. Perineural invasion: not identified
5. Margins Bronchial: 6 cm
6. Pleural/extrapleural
   Tumor superficially invading in the pleural connective tissue,
   but not beyond the elastic layer of the visceral pleura (PL0)
7. Regional lymph nodes included in main specimen (N1)
   a. Total number examined: 2
   b. Number involved by tumor: 0
8. Separately submitted N1 or N2 lymph nodes
   a. Total number examined : 11
   b. Number involved by tumor: 0
      (0/11: "5", 0/1; "7", 0/3; "L9", 0/3; "L10", 0/1; "L11", 0/3)
9. Additional pathologic findings: Background lung; unremarkable
