# Supplementary material for: A High-Dimensional, Deep-Sequencing Study of Lung Adenocarcinoma in Female Never-Smokers
Source: PLoS One. 2013 Feb 6;8(2):e55596. doi: 10.1371/journal.pone.0055596 (PMC3566005; doi:10.1371/journal.pone.0055596)
Supplement: File S6 — Gene lists with brief summaries for somatic mutations and network modules. (PDF) [file pone.0055596.s006.pdf]

Suppl. File 6. Gene lists with brief summaries for somatic mutations, fusion transcripts, and network modules

< List of somatic mutations experimentally confirmed >

Gene Name	Official Full Name	NCBI Gene Summary
BAZ1B	bromodomain adjacent to zinc finger domain, 1B	This gene encodes a member of the bromodomain protein family. The bromodomain is a structural motif characteristic of proteins involved in chromatin-dependent regulation of transcription. This gene is deleted in Williams-Beuren syndrome, a developmental disorder caused by deletion of multiple genes at 7q11.23.
GLP2R	glucagon-like peptide 2 receptor	The GLP2 receptor (GLP2R) is a G protein-coupled receptor superfamily member closely related to the glucagon receptor ans GLP1 receptor. Glucagon-like peptide-2 (GLP2) is a 33-amino acid proglucagon-derived peptide produced by intestinal enteroendocrine cells. Like glucagon-like peptide-1 (GLP1) and glucagon itself, it is derived from the proglucagon peptide encoded by the GCG gene. GLP2 stimulates intestinal growth and upregulates villus height in the small intestine, concomitant with increased crypt cell proliferation and decreased enterocyte apoptosis. Moreover, GLP2 prevents intestinal hypoplasia resulting from total parenteral nutrition. GLP2R, a G protein-coupled receptor superfamily member is expressed in the gut and closely related to the glucagon receptor (GCGR) and the receptor for GLP1 (GLP1R).
IRAK3	interleukin-1 receptor-associated kinase 3	This gene encodes a member of the interleukin-1 receptor-associated kinase protein family. Members of this family are essential components of the Toll/IL-R immune signal transduction pathways. This protein is primarily expressed in monocytes and macrophages and functions as a negative regulator of Toll-like receptor signaling. Mutations in this gene are associated with a susceptibility to asthma. Alternate splicing results in multiple transcript variants.
KIR3DL1	killer cell immunoglobulin-like receptor, three domains, long cytoplasmic tail, 1	Killer cell immunoglobulin-like receptors (KIRs) are transmembrane glycoproteins expressed by natural killer cells and subsets of T cells. The KIR genes are polymorphic and highly homologous and they are found in a cluster on chromosome 19q13.4 within the 1 Mb leukocyte receptor complex (LRC). The gene content of the KIR gene cluster varies among haplotypes, although several "framework" genes are found in all haplotypes (KIR3DL3, KIR3DP1, KIR3DL4, KIR3DL2). The KIR proteins are classified by the number of extracellular immunoglobulin domains (2D or 3D) and by whether they have a long (L) or short (S) cytoplasmic domain. KIR proteins with the long cytoplasmic domain transduce inhibitory signals upon ligand binding via an immune tyrosine-based inhibitory motif (ITIM), while KIR proteins with the short cytoplasmic domain lack the ITIM motif and instead associate with the TYRO protein tyrosine kinase binding protein to transduce activating signals. The ligands for several KIR proteins are subsets of HLA class I molecules; thus, KIR proteins are thought to play an important role in regulation of the immune response.
OCIAD1	OCIA domain containing 1	
OCRL	oculocerebrorenal syndrome of Lowe	This gene encodes a phosphatase enzyme that is involved in actin polymerization and is found in the trans-Golgi network. Mutations in this gene cause oculocerebrorenal syndrome of Lowe and also Dent disease.
OR6K3	olfactory receptor, family 6, subfamily K, member 3	Olfactory receptors interact with odorant molecules in the nose, to initiate a neuronal response that triggers the perception of a smell. The olfactory receptor proteins are members of a large family of G-protein-coupled receptors (GPCR) arising from single coding-exon genes. Olfactory receptors share a 7-transmembrane domain structure with many neurotransmitter and hormone receptors and are responsible for the recognition and G protein-mediated transduction of odorant signals. The olfactory receptor gene family is the largest in the genome. The nomenclature assigned to the olfactory receptor genes and proteins for this organism is independent of other organisms.
PSMD6	proteasome (prosome, macropain) 26S subunit, non-ATPase, 6	
RANBP9	RAN binding protein 9	This gene encodes a protein that binds RAN, a small GTP binding protein belonging to the RAS superfamily that is essential for the translocation of RNA and proteins through the nuclear pore complex. The protein encoded by this gene has also been shown to interact with several other proteins, including met proto-oncogene, homeodomain interacting protein kinase 2, androgen receptor, and cyclin-dependent kinase 11.
ZNF729		
EML1	echinoderm microtubule associated protein like 1	Human echinoderm microtubule-associated protein-like is a strong candidate for the Usher syndrome type 1A gene. Usher syndromes (USHs) are a group of genetic disorders consisting of congenital deafness, retinitis pigmentosa, and vestibular dysfunction of variable onset and severity depending on the genetic type. The disease process in USHs involves the entire brain and is not limited to the posterior fossa or auditory and visual systems. The USHs are catagorized as type I (USH1A, USH1B, USH1C, USH1D, USH1E and USH1F), type II (USH2A and USH2B) and type III (USH3). The type I is the most severe form. Gene loci responsible for these three types are all mapped. Two transcript variants encoding different isoforms have been found for this gene.
POLN	polymerase (DNA directed) nu	
ZNF536	zinc finger protein 536	
C11orf82	chromosome 11 open reading frame 82	
UBASH3A	ubiquitin associated and SH3 domain containing A	This gene encodes one of two family members belonging to the T-cell ubiquitin ligand (TULA) family. Both family members can negatively regulate T-cell signaling. This family member can facilitate growth factor withdrawal-induced apoptosis in T cells, which may occur via its interaction with AIF, an apoptosis-inducing factor. Alternative splicing of this gene results in multiple transcript variants.
HPS5	Hermansky-Pudlak syndrome 5	This gene encodes a protein that may play a role in organelle biogenesis associated with melanosomes, platelet dense granules, and lysosomes. This protein interacts with Hermansky-Pudlak syndrome 6 protein and may interact with the cytoplasmic domain of integrin, alpha-3. Mutations in this gene are associated with Hermansky-Pudlak syndrome type 5. Multiple transcript variants encoding two distinct isoforms have been identified for this gene.
C6orf118	chromosome 6 open reading frame 118	
PEX1	peroxisomal biogenesis factor 1	This gene encodes a member of the AAA ATPase family, a large group of ATPases associated with diverse cellular activities. This protein is cytoplasmic but is often anchored to a peroxisomal membrane where it forms a heteromeric complex and plays a role in the import of proteins into peroxisomes and peroxisome biogenesis. Mutations in this gene have been associated with complementation group 1 peroxisomal disorders such as neonatal adrenoleukodystrophy, infantile Refsum disease, and Zellweger syndrome.
THOC2	THO complex 2	The TREX multiprotein complex binds specifically to spliced mRNAs to facilitate mRNA export. The protein encoded by this gene is a member of the THO complex, a subset of the TREX complex. The encoded protein interacts with the THOC1 protein.
FBXO11	F-box protein 11	This gene encodes a member of the F-box protein family which is characterized by an approximately 40 amino acid motif, the F-box. The F-box proteins constitute one of the four subunits of ubiquitin protein ligase complex called SCFs (SKP1-cullin-F-box), which function in phosphorylation-dependent ubiquitination. The F-box proteins are divided into 3 classes: Fbws containing WD-40 domains, Fbls containing leucine-rich repeats, and Fbxs containing either different protein-protein interaction modules or no recognizable motifs. The protein encoded by this gene belongs to the Fbxs class. It can function as an arginine methyltransferase that symmetrically dimethylates arginine residues, and it acts as an adaptor protein to mediate

		the neddylation of p53, which leads to the suppression of p53 function. This gene is known to be down-regulated in melanocytes from patients with vitiligo, a skin disorder that results in depigmentation. Polymorphisms in this gene are associated with chronic otitis media with effusion and recurrent otitis media (COME/ROM), a hearing loss disorder, and the knockout of the homologous mouse gene results in the deaf mouse mutant Jeff (Jf), a single gene model of otitis media. Alternatively spliced transcript variants encoding distinct isoforms have been identified for this gene.
AKAP9	A kinase (PRKA) anchor protein (yotiao) 9	The A-kinase anchor proteins (AKAPs) are a group of structurally diverse proteins which have the common function of binding to the regulatory subunit of protein kinase A (PKA) and confining the holoenzyme to discrete locations within the cell. This gene encodes a member of the AKAP family. Alternate splicing of this gene results in at least two isoforms that localize to the centrosome and the Golgi apparatus, and interact with numerous signaling proteins from multiple signal transduction pathways. These signaling proteins include type II protein kinase A, serine/threonine kinase protein kinase N, protein phosphatase 1, protein phosphatase 2a, protein kinase C-epsilon and phosphodiesterase 4D3.
HTR1E	5-hydroxytryptamine (serotonin) receptor 1E, G protein-coupled	
TUBB1	tubulin, beta 1 class VI	This gene encodes a member of the beta tubulin protein family. Beta tubulins are one of two core protein families (alpha and beta tubulins) that heterodimerize and assemble to form microtubules. This protein is specifically expressed in platelets and megakaryocytes and may be involved in proplatelet production and platelet release. A mutations in this gene is associated with autosomal dominant macrothrombocytopenia. Two pseudogenes of this gene are found on chromosome Y.
MAGEE1	melanoma antigen family E, 1	This gene encodes an alpha-dystrobrevin-associated MAGE (melanoma-associated antigen) protein, which is a member of the MAGE family. The protein contains a nuclear localization signal in the N-terminus, 30 12-amino acid repeats beginning at nt 60 with the consensus sequence ASEGPSTSVLPT, and two MAGE domains in the C-terminus. It may play a signaling role in brain, muscle, and peripheral nerve. This gene is located on X chromosome in a region containing loci linked to mental retardation.
LAMB1	laminin, beta 1	Laminins, a family of extracellular matrix glycoproteins, are the major noncollagenous constituent of basement membranes. They have been implicated in a wide variety of biological processes including cell adhesion, differentiation, migration, signaling, neurite outgrowth and metastasis. Laminins are composed of 3 non identical chains: laminin alpha, beta and gamma (formerly A, B1, and B2, respectively) and they form a cruciform structure consisting of 3 short arms, each formed by a different chain, and a long arm composed of all 3 chains. Each laminin chain is a multidomain protein encoded by a distinct gene. Several isoforms of each chain have been described. Different alpha, beta and gamma chain isomers combine to give rise to different heterotrimeric laminin isoforms which are designated by Arabic numerals in the order of their discovery, i.e. alpha1beta1gamma1 heterotrimer is laminin 1. The biological functions of the different chains and trimer molecules are largely unknown, but some of the chains have been shown to differ with respect to their tissue distribution, presumably reflecting diverse functions in vivo. This gene encodes the beta chain isoform laminin, beta 1. The beta 1 chain has 7 structurally distinct domains which it shares with other beta chain isomers. The C-terminal helical region containing domains I and II are separated by domain alpha, domains III and V contain several EGF-like repeats, and domains IV and VI have a globular conformation. Laminin, beta 1 is expressed in most tissues that produce basement membranes, and is one of the 3 chains constituting laminin 1, the first laminin isolated from Engelbreth-Holm-Swarm (EHS) tumor. A sequence in the beta 1 chain that is involved in cell attachment, chemotaxis, and binding to the laminin receptor was identified and shown to have the capacity to inhibit metastasis.
ST3GAL3	ST3 beta-galactoside alpha-2,3-sialyltransferase 3	The protein encoded by this gene is a type II membrane protein that catalyzes the transfer of sialic acid from CMP-sialic acid to galactose-containing substrates. The encoded protein is normally found in the Golgi apparatus but can be proteolytically processed to a soluble form. This protein is a member of glycosyltransferase family 29. Multiple transcript variants encoding several different isoforms have been found for this gene.
COPS3	COP9 constitutive photomorphogenic homolog subunit 3 (Arabidopsis)	The protein encoded by this gene possesses kinase activity that phosphorylates regulators involved in signal transduction. It phosphorylates I kappa-Balpha, p105, and c-Jun. It acts as a docking site for complex-mediated phosphorylation. The gene is located within the Smith-Magenis syndrome region on chromosome 17. Two transcript variants encoding different isoforms have been found for this gene.
DNAH8	dynein, axonemal, heavy chain 8	The protein encoded by this gene is a heavy chain of an axonemal dynein involved in sperm and respiratory cilia motility. Axonemal dyneins generate force through hydrolysis of ATP and binding to microtubules.
DYRK3	dual-specificity tyrosine-(Y)-phosphorylation regulated kinase 3	This gene product belongs to the DYRK family of dual-specificity protein kinases that catalyze autophosphorylation on serine/threonine and tyrosine residues. The members of this family share structural similarity, however, differ in their substrate specificity, suggesting their involvement in different cellular functions. The encoded protein has been shown to autophosphorylate on tyrosine residue and catalyze phosphorylation of histones H3 and H2B in vitro. Alternatively spliced transcript variants encoding different isoforms have been identified.
GUSB	glucuronidase, beta	This gene encodes a hydrolase that degrades glycosaminoglycans, including heparan sulfate, dermatan sulfate, and chondroitin-4,6-sulfate. The enzyme forms a homotetramer that is localized to the lysosome. Mutations in this gene result in mucopolysaccharidosis type VII. There are many pseudogenes of this locus in the human genome.
AXDND1	axonemal dynein light chain domain containing 1	
UBR4	ubiquitin protein ligase E3 component n-recognin 4	The protein encoded by this gene is an E3 ubiquitin-protein ligase that interacts with the retinoblastoma-associated protein in the nucleus and with calcium-bound calmodulin in the cytoplasm. The encoded protein appears to be a cytoskeletal component in the cytoplasm and part of the chromatin scaffold in the nucleus. In addition, this protein is a target of the human papillomavirus type 16 E7 oncoprotein.
L3MBTL2	l(3)mbt-like 2 (Drosophila)	
RHAG	Rh-associated glycoprotein	The protein encoded by this gene is erythrocyte-specific and is thought to be part of a membrane channel that transports ammonium and carbon dioxide across the blood cell membrane. The encoded protein appears to interact with Rh blood group antigens and Rh30 polypeptides. Defects in this gene are a cause of regulator type Rh-null hemolytic anemia (RHN), or Rh-deficiency syndrome.[provided by RefSeq, Mar 2009]
SH3PXD2A	SH3 and PX domains 2A	
HLTF	helicase-like transcription factor	This gene encodes a member of the SWI/SNF family. Members of this family have helicase and ATPase activities and are thought to regulate transcription of certain genes by altering the chromatin structure around those genes. The encoded protein contains a RING finger DNA binding motif. Two transcript variants encoding the same protein have been found for this gene. However, use of an alternative translation start site produces an isoform that is truncated at the N-terminus compared to the full-length protein.
HUNK	hormonally up-regulated Neu-associated kinase	
ACCN1	acid-sensing (proton-gated) ion channel 2	This gene encodes a member of the degenerin/epithelial sodium channel (DEG/ENaC) superfamily. The members of this family are amiloride-sensitive sodium channels that contain intracellular N and C termini, 2 hydrophobic transmembrane regions, and a large extracellular loop, which has many cysteine residues with conserved spacing. The member encoded by this gene may play a role in neurotransmission. In addition, a heteromeric association between this member and acid-sensing (proton-gated) ion channel 3 has been observed to co-assemble into proton-gated channels sensitive to gadolinium. Alternative splicing has been observed at this locus and two variants, encoding distinct isoforms, have been identified.
STX2	syntaxin 2	The product of this gene belongs to the syntaxin/epimorphin family of proteins. The syntaxins are a large protein family implicated in the targeting and fusion of intracellular transport vesicles. The product of this gene regulates epithelial-mesenchymal interactions and epithelial cell morphogenesis and activation. Alternatively spliced transcript variants encoding different isoforms have been identified.
PRG4	proteoglycan 4	The protein encoded by this gene is a large proteoglycan specifically synthesized by chondrocytes located at the surface of articular cartilage, and also by some synovial lining cells. This protein contains both chondroitin sulfate and keratan sulfate glycosaminoglycans. It functions as a boundary lubricant at the cartilage surface and contributes to the elastic absorption and energy dissipation of synovial fluid. Mutations in this gene result in camptodactyly-arthropathy-coxa vara-pericarditis syndrome. Multiple transcript variants encoding different isoforms have been found for this gene.
FRMD5	FERM domain containing 5	

ERC1	ELKS/RAB6-interacting/CAST family member 1	The protein encoded by this gene is a member of a family of RIM-binding proteins. RIMs are active zone proteins that regulate neurotransmitter release. This gene has been found fused to the receptor-type tyrosine kinase gene RET by gene rearrangement due to the translocation t(10;12)(q11;p13). Multiple transcript variants encoding different isoforms have been found for this gene.
COL19A1	collagen, type XIX, alpha 1	This gene encodes the alpha chain of type XIX collagen, a member of the FACIT collagen family (fibril-associated collagens with interrupted helices). Although the function of this collagen is not known, other members of this collagen family are found in association with fibril-forming collagens such as type I and II, and serve to maintain the integrity of the extracellular matrix. The transcript produced from this gene has an unusually large 3' UTR which has not been completely sequenced.
P2RY1	purinergic receptor P2Y, G-protein coupled, 1	The product of this gene belongs to the family of G-protein coupled receptors. This family has several receptor subtypes with different pharmacological selectivity, which overlaps in some cases, for various adenosine and uridine nucleotides. This receptor functions as a receptor for extracellular ATP and ADP. In platelets binding to ADP leads to mobilization of intracellular calcium ions via activation of phospholipase C, a change in platelet shape, and probably to platelet aggregation.
CELF4	CUGBP, Elav-like family member 4	Members of the CELF/BRUNOL protein family contain two N-terminal RNA recognition motif (RRM) domains, one C-terminal RRM domain, and a divergent segment of 160-230 aa between the second and third RRM domains. Members of this protein family regulate pre-mRNA alternative splicing and may also be involved in mRNA editing, and translation. Multiple transcript variants encoding different isoforms have been found for this gene.
MAGEA2	melanoma antigen family A, 2	This gene is a member of the MAGEA gene family. The members of this family encode proteins with 50 to 80% sequence identity to each other. The promoters and first exons of the MAGEA genes show considerable variability, suggesting that the existence of this gene family enables the same function to be expressed under different transcriptional controls. The MAGEA genes are clustered at chromosomal location Xq28. They have been implicated in some hereditary disorders, such as dyskeratosis congenita. This gene has two identical copies at different loci. Alternatively spliced transcript variants encoding the same protein have been identified for this gene.

< List of fusion transcripts experimentally confirmed >

Gene Name	Official Full Name	NCBI Gene Summary
RHPN2	rhophilin, Rho GTPase binding protein 2	This gene encodes a member of the rhophilin family of Ras-homologous (Rho)-GTPase binding proteins. The encoded protein binds both GTP- and GDP-bound RhoA and GTP-bound RhoB and may be involved in the organization of the actin cytoskeleton.
SIRT2	sirtuin 2	This gene encodes a member of the sirtuin family of proteins, homologs to the yeast Sir2 protein. Members of the sirtuin family are characterized by a sirtuin core domain and grouped into four classes. The functions of human sirtuins have not yet been determined; however, yeast sirtuin proteins are known to regulate epigenetic gene silencing and suppress recombination of rDNA. Studies suggest that the human sirtuins may function as intracellular regulatory proteins with mono-ADP-ribosyltransferase activity. The protein encoded by this gene is included in class I of the sirtuin family. Several transcript variants are resulted from alternative splicing of this gene.
COX6C	cytochrome c oxidase subunit VIc	Cytochrome c oxidase, the terminal enzyme of the mitochondrial respiratory chain, catalyzes the electron transfer from reduced cytochrome c to oxygen. It is a heteromeric complex consisting of 3 catalytic subunits encoded by mitochondrial genes and multiple structural subunits encoded by nuclear genes. The mitochondrially-encoded subunits function in electron transfer, and the nuclear-encoded subunits may be involved in the regulation and assembly of the complex. This nuclear gene encodes subunit VIc, which has 77% amino acid sequence identity with mouse subunit VIc. This gene is up-regulated in prostate cancer cells. A pseudogene has been found on chromosomes 16p12.
MARK4	MAP/microtubule affinity-regulating kinase 4	This gene encodes a member of the microtubule affinity-regulating kinase family. These protein kinases phosphorylate microtubule-associated proteins and regulate the transition between stable and dynamic microtubules. The encoded protein is associated with the centrosome throughout mitosis and may be involved in cell cycle control. Expression of this gene is a potential marker for cancer, and the encoded protein may also play a role in Alzheimer's disease. Pseudogenes of this gene are located on both the short and long arm of chromosome 3. Alternatively spliced transcript variants encoding multiple isoforms have been observed for this gene.
NDUFB9	NADH dehydrogenase (ubiquinone) 1 beta subcomplex, 9, 22kDa	
SLC6A9	solute carrier family 6 (neurotransmitter transporter, glycine), member 9	
STK3	serine/threonine kinase 3	This gene encodes a serine/threonine protein kinase activated by proapoptotic molecules indicating the encoded protein functions as a growth suppressor. Cleavage of the protein product by caspase removes the inhibitory C-terminal portion. The N-terminal portion is transported to the nucleus where it homodimerizes to form the active kinase which promotes the condensation of chromatin during apoptosis. Multiple transcript variants encoding different isoforms have been found for this gene.
PTK2	PTK2 protein tyrosine kinase 2	This gene encodes a cytoplasmic protein tyrosine kinase which is found concentrated in the focal adhesions that form between cells growing in the presence of extracellular matrix constituents. The encoded protein is a member of the FAK subfamily of protein tyrosine kinases but lacks significant sequence similarity to kinases from other subfamilies. Activation of this gene may be an important early step in cell growth and intracellular signal transduction pathways triggered in response to certain neural peptides or to cell interactions with the extracellular matrix. Several transcript variants encoding different isoforms have been found for this gene, but the full-length natures of only three of them have been determined.
PKHD1L1	polycystic kidney and hepatic disease 1 (autosomal recessive)-like 1	
MKL1	megakaryoblastic leukemia (translocation) 1	The protein encoded by this gene interacts with the transcription factor myocardin, a key regulator of smooth muscle cell differentiation. The encoded protein is predominantly nuclear and may help transduce signals from the cytoskeleton to the nucleus. This gene is involved in a specific translocation event that creates a fusion of this gene and the RNA-binding motif protein-15 gene. This translocation has been associated with acute megakaryocytic leukemia.
HSPG2	heparan sulfate proteoglycan 2	This gene encodes the perlecan protein, which consists of a core protein to which three long chains of glycosaminoglycans (heparan sulfate or chondroitin sulfate) are attached. The perlecan protein is a large multidomain proteoglycan that binds to and cross-links many extracellular matrix components and cell-surface molecules. It has been shown that this protein interacts with laminin, prolargin, collagen type IV, FGFBP1, FBLN2, FGF7 and Transthyretin, etc. and plays essential roles in multiple biological activities. Perlecan is a key component of the vascular extracellular matrix, where it helps to maintain the endothelial barrier function. It is a potent inhibitor of smooth muscle cell proliferation and is thus thought to help maintain vascular homeostasis. It can also promote growth factor (e.g., FGF2) activity and thus stimulate endothelial growth and re-generation. It is a major component of basement membranes, where it is involved in the stabilization of other molecules as well as being involved with glomerular permeability to macromolecules and cell adhesion. Mutations in this gene cause Schwartz-Jampel syndrome type 1, Silverman-Handmaker type of dyssegmental dysplasia, and Tardive dyskinesia.
NIPAL3	NIPA-like domain containing 3	
UBFD1	ubiquitin family domain containing 1	

SLC7A6	solute carrier family 7 (amino acid transporter light chain, y+L system), member 6	
KDM6A	lysine (K)-specific demethylase 6A	This gene is located on the X chromosome and is the corresponding locus to a Y-linked gene which encodes a tetratricopeptide repeat (TPR) protein. The encoded protein of this gene contains a JmjC-domain and catalyzes the demethylation of tri/dimethylated histone H3.
EIF1AX	eukaryotic translation initiation factor 1A, X-linked	This gene encodes an essential eukaryotic translation initiation factor. The protein is required for the binding of the 43S complex (a 40S subunit, eIF2/GTP/Met-tRNAi and eIF3) to the 5' end of capped RNA.
GRHL2	grainyhead-like 2 (Drosophila)	The protein encoded by this gene is a transcription factor that can act as a homodimer or as a heterodimer with either GRHL1 or GRHL3. Defects in this gene are a cause of non-syndromic sensorineural deafness autosomal dominant type 28 (DFNA28).
CCDC6	coiled-coil domain containing 6	This gene encodes a coiled-coil domain-containing protein. The encoded protein is ubiquitously expressed and may function as a tumor suppressor. A chromosomal rearrangement resulting in the expression of a fusion gene containing a portion of this gene and the intracellular kinase-encoding domain of the ret proto-oncogene is the cause of thyroid papillary carcinoma.
GLE1	GLE1 RNA export mediator homolog (yeast)	This gene encodes a predicted 75-kDa polypeptide with high sequence and structure homology to yeast Gle1p, which is nuclear protein with a leucine-rich nuclear export sequence essential for poly(A)+RNA export. Inhibition of human GLE1L by microinjection of antibodies against GLE1L in HeLa cells resulted in inhibition of poly(A)+RNA export. Immunoflourescence studies show that GLE1L is localized at the nuclear pore complexes. This localization suggests that GLE1L may act at a terminal step in the export of mature RNA messages to the cytoplasm. Two alternatively spliced transcript variants encoding different isoforms have been found for this gene.
PEPD	peptidase D	This gene encodes a member of the peptidase family. The protein forms a homodimer that hydrolyzes dipeptides or tripeptides with C-terminal proline or hydroxyproline residues. The enzyme serves an important role in the recycling of proline, and may be rate limiting for the production of collagen. Mutations in this gene result in prolidase deficiency, which is characterized by the excretion of large amount of di- and tri-peptides containing proline. Multiple transcript variants encoding different isoforms have been found for this gene.
NPHS1	nephrosis 1, congenital, Finnish type (nephrin)	This gene encodes a member of the immunoglobulin family of cell adhesion molecules that functions in the glomerular filtration barrier in the kidney. The gene is primarily expressed in renal tissues, and the protein is a type-1 transmembrane protein found at the slit diaphragm of glomerular podocytes. The slit diaphragm is thought to function as an ultrafilter to exclude albumin and other plasma macromolecules in the formation of urine. Mutations in this gene result in Finnish-type congenital nephrosis 1, characterized by severe proteinuria and loss of the slit diaphragm and foot processes.
LAPTM4B	lysosomal protein transmembrane 4 beta	
ERCC2	excision repair cross-complementing rodent repair deficiency, complementation group 2	The nucleotide excision repair pathway is a mechanism to repair damage to DNA. The protein encoded by this gene is involved in transcription-coupled nucleotide excision repair and is an integral member of the basal transcription factor BTF2/TFIIH complex. The gene product has ATP-dependent DNA helicase activity and belongs to the RAD3/XPD subfamily of helicases. Defects in this gene can result in three different disorders, the cancer-prone syndrome xeroderma pigmentosum complementation group D, trichothiodystrophy, and Cockayne syndrome. Alternatively spliced transcript variants encoding different isoforms have been found for this gene.
PGCP	carboxypeptidase Q	
MORN1	MORN repeat containing 1	
FAM84B	family with sequence similarity 84, member B	
MATN2	matrilin 2	This gene encodes a member of the von Willebrand factor A domain containing protein family. This family of proteins is thought to be involved in the formation of filamentous networks in the extracellular matrices of various tissues. This protein contains five von Willebrand factor A domains. The specific function of this gene has not yet been determined. Two transcript variants encoding different isoforms have been found for this gene.
NIPA1	non imprinted in Prader-Willi/Angelman syndrome 1	This gene encodes a magnesium transporter that associates with early endosomes and the cell surface in a variety of neuronal and epithelial cells. This protein may play a role in nervous system development and maintenance. Multiple transcript variants encoding different isoforms have been found for this gene. Mutations in this gene have been associated with autosomal dominant spastic paraplegia 6.
TMCO4	transmembrane and coiled-coil domains 4	
ATAD3B	ATPase family, AAA domain containing 3B	ATAD3A (MIM 612316) and ATAD3B are mitochondrial membrane proteins that contribute to the stabilization of large mitochondrial DNA (mtDNA)-protein complexes called nucleoids (He et al., 2007 [PubMed 17210950]).
CDH11	cadherin 11, type 2, OB-cadherin (osteoblast)	This gene encodes a type II classical cadherin from the cadherin superfamily, integral membrane proteins that mediate calcium-dependent cell-cell adhesion. Mature cadherin proteins are composed of a large N-terminal extracellular domain, a single membrane-spanning domain, and a small, highly conserved C-terminal cytoplasmic domain. Type II (atypical) cadherins are defined based on their lack of a HAV cell adhesion recognition sequence specific to type I cadherins. Expression of this particular cadherin in osteoblastic cell lines, and its upregulation during differentiation, suggests a specific function in bone development and maintenance.
LRRC36	leucine rich repeat containing 36	
WSB1	WD repeat and SOCS box containing 1	This gene encodes a member of the WD-protein subfamily. This protein shares a high sequence identity to mouse and chick proteins. It contains several WD-repeats spanning most of the protein and an SOCS box in the C-terminus. Alternatively spliced transcript variants encoding distinct isoforms have been found for this gene.
PDE4DIP	phosphodiesterase 4D interacting protein	The protein encoded by this gene serves to anchor phosphodiesterase 4D to the Golgi/centrosome region of the cell. Defects in this gene may be a cause of myeloproliferative disorder (MBD) associated with eosinophilia. Several transcript variants encoding different isoforms have been found for this gene.
PTPN12	protein tyrosine phosphatase, non-receptor type 12	The protein encoded by this gene is a member of the protein tyrosine phosphatase (PTP) family. PTPs are signaling molecules that regulate a variety of cellular processes including cell growth, differentiation, mitotic cycle, and oncogenic transformation. This PTP contains a C-terminal PEST motif, which serves as a protein-protein interaction domain, and may regulate protein intracellular half-life. This PTP was found to bind and dephosphorylate the product of the oncogene c-ABL and thus may play a role in oncogenesis. This PTP was also shown to interact with, and dephosphorylate, various products related to cytoskeletal structure and cell adhesion, such as p130 (Cas), CAKbeta/PTK2B, PSTPIP1, and paxillin. This suggests it has a regulatory role in controlling cell shape and mobility. Alternative splicing results in multiple transcript variants encoding distinct isoforms.
RET	ret proto-oncogene	This gene, a member of the cadherin superfamily, encodes one of the receptor tyrosine kinases, which are cell-surface molecules that transduce signals for cell growth and differentiation. This gene plays a crucial role in neural crest development, and it can undergo oncogenic activation in vivo and in vitro by cytogenetic rearrangement. Mutations in this gene are associated with the disorders multiple endocrine neoplasia, type IIA, multiple endocrine neoplasia, type IIB, Hirschsprung disease, and medullary thyroid carcinoma. Two transcript variants encoding different isoforms have been found for this gene. Additional transcript variants have been described but their biological validity has not been confirmed.
CCBL1	cysteine conjugate-beta lyase, cytoplasmic	This gene encodes a cytosolic enzyme that is responsible for the metabolism of cysteine conjugates of certain halogenated alkenes and alkanes. This metabolism can form reactive metabolites leading to nephrotoxicity and neurotoxicity. Increased levels of this enzyme have been linked to schizophrenia. Multiple transcript variants that encode different isoforms have been identified for this gene.

< Gene list in the MCODE network modules >

Module 1 : Rho Signaling

Gene Name	Official Full Name	NCBI Gene Summary
ARAP3	ArfGAP with RhoGAP domain, ankyrin repeat and PH domain 3	This gene encodes a phosphoinositide binding protein containing ARF-GAP, RHO-GAP, RAS-associating, and pleckstrin homology domains. The ARF-GAP and RHO-GAP domains cooperate in mediating rearrangements in the cell cytoskeleton and cell shape. It is a specific PtdIns(3,4,5)P3/PtdIns(3,4)P2-stimulated Arf6-GAP protein. An alternatively spliced transcript has been found for this gene, but its biological validity has not been determined.
ARHGAP6	Rho GTPase activating protein 6	This gene encodes a member of the rhoGAP family of proteins which play a role in the regulation of actin polymerization at the plasma membrane during several cellular processes. This protein is thought to have two independent functions, one as a GTPase-activating protein with specificity for RhoA, and another as a cytoskeletal protein that promotes actin remodeling. Multiple alternatively spliced transcript variants encoding different isoforms have been found for this gene.
ARHGAP29	Rho GTPase activating protein 29	
ARHGAP31	Rho GTPase activating protein 31	This gene encodes a GTPase-activating protein (GAP). A variety of cellular processes are regulated by Rho GTPases which cycle between an inactive form bound to GDP and an active form bound to GTP. This cycling between inactive and active forms is regulated by guanine nucleotide exchange factors and GAPs. The encoded protein is a GAP shown to regulate two GTPases involved in protein trafficking and cell growth.
ARHGAP40	Rho GTPase activating protein 40	
STARD8	StAR-related lipid transfer (START) domain containing 8	This gene encodes a member of a subfamily of Rho GTPase activating proteins that contain a steroidogenic acute regulatory protein related lipid transfer domain. The encoded protein localizes to focal adhesions and may be involved in regulating cell morphology. This protein may also function as a tumor suppressor.
STARD13	StAR-related lipid transfer (START) domain containing 13	This gene encodes a protein which contains an N-terminal sterile alpha motif (SAM) for protein-protein interactions, followed by an ATP/GTP-binding motif, a GTPase-activating protein (GAP) domain, and a C-terminal STAR-related lipid transfer (START) domain. It may be involved in regulation of cytoskeletal reorganization, cell proliferation, and cell motility, and acts as a tumor suppressor in hepatoma cells. The gene is located in a region of chromosome 13 that is associated with loss of heterozygosity in hepatocellular carcinomas. Alternatively spliced transcript variants encoding different isoforms have been described for this gene
RHOB	ras homolog gene family, member B	
RHOJ	ras homolog gene family, member J	ARHJ belongs to the Rho family of small GTP-binding proteins. Rho proteins regulate the dynamic assembly of cytoskeletal components for several physiologic processes, such as cell proliferation and motility and the establishment of cell polarity. They are also involved in pathophysiologic process, such as cell transformation and metastasis.
RHOV	ras homolog gene family, member V	
A2M	alpha-2-macroglobulin	Alpha-2-macroglobulin is a protease inhibitor and cytokine transporter. It inhibits many proteases, including trypsin, thrombin and collagenase. A2M is implicated in Alzheimer disease (AD) due to its ability to mediate the clearance and degradation of A-beta, the major component of beta-amyloid deposits
SYDE1	synapse defective 1, Rho GTPase, homolog 1 (C. elegans)	
AMBP	alpha-1-microglobulin/bikunin precursor	This gene encodes a complex glycoprotein secreted in plasma. The precursor is proteolytically processed into distinct functioning proteins: alpha-1-microglobulin, which belongs to the superfamily of lipocalin transport proteins and may play a role in the regulation of inflammatory processes, and bikunin, which is a urinary trypsin inhibitor belonging to the superfamily of Kunitz-type protease inhibitors and plays an important role in many physiological and pathological processes. This gene is

		located on chromosome 9 in a cluster of lipocalin genes
PDGFB	platelet-derived growth factor beta polypeptide	The protein encoded by this gene is a member of the platelet-derived growth factor family. The four members of this family are mitogenic factors for cells of mesenchymal origin and are characterized by a motif of eight cysteines. This gene product can exist either as a homodimer (PDGF-BB) or as a heterodimer with the platelet-derived growth factor alpha polypeptide (PDGF-AB), where the dimers are connected by disulfide bonds. Mutations in this gene are associated with meningioma. Reciprocal translocations between chromosomes 22 and 7, at sites where this gene and that for COL1A1 are located, are associated with a particular type of skin tumor called dermatofibrosarcoma protuberans resulting from unregulated expression of growth factor. Two alternatively spliced transcript variants encoding different isoforms have been identified for this gene
OCRL	oculocerebrorenal syndrome of Lowe	This gene encodes a phosphatase enzyme that is involved in actin polymerization and is found in the trans-Golgi network. Mutations in this gene cause oculocerebrorenal syndrome of Lowe and also Dent disease.

Module 2 : Cell Cycle: Mitosis

Gene Name	Official Full Name	NCBI Gene Summary
CCNB1	cyclin B1	The protein encoded by this gene is a regulatory protein involved in mitosis. The gene product complexes with p34(cdc2) to form the maturation-promoting factor (MPF). Two alternative transcripts have been found, a constitutively expressed transcript and a cell cycle-regulated transcript, that is expressed predominantly during G2/M phase. The different transcripts result from the use of alternate transcription initiation sites
CCNB2	cyclin B2	Cyclin B2 is a member of the cyclin family, specifically the B-type cyclins. The B-type cyclins, B1 and B2, associate with p34cdc2 and are essential components of the cell cycle regulatory machinery. B1 and B2 differ in their subcellular localization. Cyclin B1 co-localizes with microtubules, whereas cyclin B2 is primarily associated with the Golgi region. Cyclin B2 also binds to transforming growth factor beta RII and thus cyclin B2/cdc2 may play a key role in transforming growth factor beta-mediated cell cycle control.
CHEK1	CHK1 checkpoint homolog (S. pombe)	
MCM10	minichromosome maintenance complex component 10	The protein encoded by this gene is one of the highly conserved mini-chromosome maintenance proteins (MCM) that are involved in the initiation of eukaryotic genome replication. The hexameric protein complex formed by MCM proteins is a key component of the pre-replication complex (pre-RC) and it may be involved in the formation of replication forks and in the recruitment of other DNA replication related proteins. This protein can interact with MCM2 and MCM6, as well as with the origin recognition protein ORC2. It is regulated by proteolysis and phosphorylation in a cell cycle-dependent manner. Studies of a similar protein in Xenopus suggest that the chromatin binding of this protein at the onset of DNA replication is after pre-RC assembly and before origin unwinding. Alternatively spliced transcript variants encoding distinct isoforms have been identified.
CDC6	cell division cycle 6 homolog (S. cerevisiae)	The protein encoded by this gene is highly similar to Saccharomyces cerevisiae Cdc6, a protein essential for the initiation of DNA replication. This protein functions as a regulator at the early steps of DNA replication. It localizes in cell nucleus during cell cyle G1, but translocates to the cytoplasm at the start of S phase. The subcellular translocation of this protein during cell cyle is regulated through its phosphorylation by Cdks. Transcription of this protein was reported to be regulated in response to mitogenic signals through transcriptional control mechanism involving E2F proteins.
CDC20	cell division cycle 20 homolog (S. cerevisiae)	CDC20 appears to act as a regulatory protein interacting with several other proteins at multiple points in the cell cycle. It is required for two microtubule-dependent processes, nuclear movement prior to anaphase and chromosome separation.

CDC45	cell division cycle 45 homolog (S. cerevisiae)	The protein encoded by this gene was identified by its strong similarity with Saccharomyces cerevisiae Cdc45, an essential protein required to the initiation of DNA replication. Cdc45 is a member of the highly conserved multiprotein complex including Cdc6/Cdc18, the minichromosome maintenance proteins (MCMs) and DNA polymerase, which is important for early steps of DNA replication in eukaryotes. This protein has been shown to interact with MCM7 and DNA polymerase alpha. Studies of the similar gene in Xenopus suggested that this protein play a pivotal role in the loading of DNA polymerase alpha onto chromatin. Multiple alternatively spliced transcript variants encoding different isoforms have been found for this gene
BUB1B	budding uninhibited by benzimidazoles 1 homolog beta (yeast)	This gene encodes a kinase involved in spindle checkpoint function. The protein has been localized to the kinetochore and plays a role in the inhibition of the anaphase-promoting complex/cyclosome (APC/C), delaying the onset of anaphase and ensuring proper chromosome segregation. Impaired spindle checkpoint function has been found in many forms of cancer.
NEK2	NIMA (never in mitosis gene a)-related kinase 2	This gene encodes a serine/threonine-protein kinase that is involved in mitotic regulation. This protein is localized to the centrosome, and undetectable during G1 phase, but accumulates progressively throughout the S phase, reaching maximal levels in late G2 phase. Alternatively spliced transcript variants encoding different isoforms with distinct C-termini have been noted for this gene.
NDC80	NDC80 homolog, kinetochore complex component (S. cerevisiae)	HEC is one of several proteins involved in spindle checkpoint signaling. This surveillance mechanism assures correct segregation of chromosomes during cell division by detecting unaligned chromosomes and causing prometaphase arrest until the proper bipolar attachment of chromosomes is achieved
MAD2L1	MAD2 mitotic arrest deficient-like 1 (yeast)	MAD2L1 is a component of the mitotic spindle assembly checkpoint that prevents the onset of anaphase until all chromosomes are properly aligned at the metaphase plate. MAD2L1 is related to the MAD2L2 gene located on chromosome 1. A MAD2 pseudogene has been mapped to chromosome 14.
ZWINT	ZW10 interactor	This gene encodes a protein that is clearly involved in kinetochore function although an exact role is not known. It interacts with ZW10, another kinetochore protein, possibly regulating the association between ZW10 and kinetochores. The encoded protein localizes to prophase kinetochores before ZW10 does and it remains detectable on the kinetochore until late anaphase. It has a uniform distribution in the cytoplasm of interphase cells. Alternatively spliced transcript variants encoding different isoforms have been found for this gene.
AURKB	aurora kinase B	Chromosomal segregation during mitosis as well as meiosis is regulated by kinases and phosphatases. The Aurora kinases associate with microtubules during chromosome movement and segregation. Aurora kinase B localizes to microtubules near kinetochores, specifically to the specialized microtubules called K-fibers, and Aurora kinase A (MIM 603072) localizes to centrosomes
BIRC5	baculoviral IAP repeat containing 5	This gene is a member of the inhibitor of apoptosis (IAP) gene family, which encode negative regulatory proteins that prevent apoptotic cell death. IAP family members usually contain multiple baculovirus IAP repeat (BIR) domains, but this gene encodes proteins with only a single BIR domain. The encoded proteins also lack a C-terminus RING finger domain. Gene expression is high during fetal development and in most tumors, yet low in adult tissues. Alternatively spliced transcript variants encoding distinct isoforms have been found for this gene.
CDCA8	cell division cycle associated 8	CDCA8 is a component of a chromosomal passenger complex required for stability of the bipolar mitotic spindle
PLK1	polo-like kinase 1	
PKMYT1	protein kinase, membrane associated tyrosine/threonine 1	The protein encoded by this gene is a member of the serine/threonine protein kinase family. This kinase preferentially phosphorylates and inactivates cell division cycle 2 protein (CDC2), and thus negatively regulates cell cycle G2/M transition. This kinase is associated with the membrane throughout the cell cycle. Its activity is highly regulated during the cell cycle. Protein kinases AKT1/PKB and PLK (Polo-like kinase) have been shown to phosphorylate and regulate the activity of this kinase. Alternatively spliced transcript variants encoding distinct isoforms have been reported.
CCNE1	cyclin E1	The protein encoded by this gene belongs to the highly conserved cyclin family, whose members are characterized by a dramatic periodicity in protein abundance through the cell cycle. Cyclins function as regulators of CDK kinases. Different

		cyclins exhibit distinct expression and degradation patterns which contribute to the temporal coordination of each mitotic event. This cyclin forms a complex with and functions as a regulatory subunit of CDK2, whose activity is required for cell cycle G1/S transition. This protein accumulates at the G1-S phase boundary and is degraded as cells progress through S phase. Overexpression of this gene has been observed in many tumors, which results in chromosome instability, and thus may contribute to tumorigenesis. This protein was found to associate with, and be involved in, the phosphorylation of NPAT protein (nuclear protein mapped to the ATM locus), which participates in cell-cycle regulated histone gene expression and plays a critical role in promoting cell-cycle progression in the absence of pRB. Two alternatively spliced transcript variants of this gene, which encode distinct isoforms, have been described. Two additional splice variants were reported but detailed nucleotide sequence information is not yet available.
MYBL2	v-myb myeloblastosis viral oncogene homolog (avian)-like 2	The protein encoded by this gene, a member of the MYB family of transcription factor genes, is a nuclear protein involved in cell cycle progression. The encoded protein is phosphorylated by cyclin A/cyclin-dependent kinase 2 during the S-phase of the cell cycle and possesses both activator and repressor activities. It has been shown to activate the cell division cycle 2, cyclin D1, and insulin-like growth factor-binding protein 5 genes. Transcript variants may exist for this gene, but their full-length natures have not been determined.
AMOTL2	angiomotin like 2	Angiomotin is a protein that binds angiostatin, a circulating inhibitor of the formation of new blood vessels (angiogenesis). Angiomotin mediates angiostatin inhibition of endothelial cell migration and tube formation in vitro. The protein encoded by this gene is related to angiomotin and is a member of the motins protein family.

Module 3 : Cellular Movement by ERK/MAPK Signaling

Gene Name	Official Full Name	NCBI Gene Summary
FYN	FYN oncogene related to SRC, FGR, YES	This gene is a member of the protein-tyrosine kinase oncogene family. It encodes a membrane-associated tyrosine kinase that has been implicated in the control of cell growth. The protein associates with the p85 subunit of phosphatidylinositol 3-kinase and interacts with the fyn-binding protein. Alternatively spliced transcript variants encoding distinct isoforms exist.
NEDD9	neural precursor cell expressed, developmentally down-regulated 9	
PRKCE	protein kinase C, epsilon	Protein kinase C (PKC) is a family of serine- and threonine-specific protein kinases that can be activated by calcium and the second messenger diacylglycerol. PKC family members phosphorylate a wide variety of protein targets and are known to be involved in diverse cellular signaling pathways. PKC family members also serve as major receptors for phorbol esters, a class of tumor promoters. Each member of the PKC family has a specific expression profile and is believed to play a distinct role in cells. The protein encoded by this gene is one of the PKC family members. This kinase has been shown to be involved in many different cellular functions, such as neuron channel activation, apoptosis, cardioprotection from ischemia, heat shock response, as well as insulin exocytosis. Knockout studies in mice suggest that this kinase is important for lipopolysaccharide (LPS)-mediated signaling in activated macrophages and may also play a role in controlling anxiety-like behavior.
PECAM1	platelet/endothelial cell adhesion molecule	The protein encoded by this gene is found on the surface of platelets, monocytes, neutrophils, and some types of T-cells, and makes up a large portion of endothelial cell intercellular junctions. The encoded protein is a member of the immunoglobulin superfamily and is likely involved in leukocyte migration, angiogenesis, and integrin activation
MAP2	microtubule-associated protein 2	This gene encodes a protein that belongs to the microtubule-associated protein family. The proteins of this family are thought to be involved in microtubule assembly, which is an essential step in neurogenesis. The products of similar genes in rat and mouse are neuron-specific cytoskeletal proteins that are enriched in dentrites, implicating a role in determining and stabilizing dentritic shape during neuron development. A number of alternatively spliced variants encoding distinct isoforms have been described

GZMB	granzyme B (granzyme 2, cytotoxic T-lymphocyte-associated serine esterase 1)	Cytolytic T lymphocytes (CTL) and natural killer (NK) cells share the remarkable ability to recognize, bind, and lyse specific target cells. They are thought to protect their host by lysing cells bearing on their surface 'nonself' antigens, usually peptides or proteins resulting from infection by intracellular pathogens. The protein encoded by this gene is crucial for the rapid induction of target cell apoptosis by CTL in cell-mediated immune response.
FGR	Gardner-Rasheed feline sarcoma viral (v-fgr) oncogene homolog	Gardner-RasheedThis gene is a member of the Src family of protein tyrosine kinases (PTKs). The encoded protein contains N-terminal sites for myristylation and palmitylation, a PTK domain, and SH2 and SH3 domains which are involved in mediating protein-protein interactions with phosphotyrosine-containing and proline-rich motifs, respectively. The protein localizes to plasma membrane ruffles, and functions as a negative regulator of cell migration and adhesion triggered by the beta-2 integrin signal transduction pathway. Infection with Epstein-Barr virus results in the overexpression of this gene. Multiple alternatively spliced variants, encoding the same protein, have been identified. feline sarcoma viral (v-fgr) oncogene homolog
PTK2	PTK2 protein tyrosine kinase 2	This gene encodes a cytoplasmic protein tyrosine kinase which is found concentrated in the focal adhesions that form between cells growing in the presence of extracellular matrix constituents. The encoded protein is a member of the FAK subfamily of protein tyrosine kinases but lacks significant sequence similarity to kinases from other subfamilies. Activation of this gene may be an important early step in cell growth and intracellular signal transduction pathways triggered in response to certain neural peptides or to cell interactions with the extracellular matrix. Several transcript variants encoding different isoforms have been found for this gene, but the full-length natures of only three of them have been determined.
RET	ret proto-oncogene	This gene, a member of the cadherin superfamily, encodes one of the receptor tyrosine kinases, which are cell-surface molecules that transduce signals for cell growth and differentiation. This gene plays a crucial role in neural crest development, and it can undergo oncogenic activation in vivo and in vitro by cytogenetic rearrangement. Mutations in this gene are associated with the disorders multiple endocrine neoplasia, type IIA, multiple endocrine neoplasia, type IIB, Hirschsprung disease, and medullary thyroid carcinoma. Two transcript variants encoding different isoforms have been found for this gene. Additional transcript variants have been described but their biological validity has not been confirmed.

Module 4 : IL-8 Antigen Presentation

Gene Name	Official Full Name	NCBI Gene Summary
CXCR1	chemokine (C-X-C motif) receptor 1	The protein encoded by this gene is a member of the G-protein-coupled receptor family. This protein is a receptor for interleukin 8 (IL8). It binds to IL8 with high affinity, and transduces the signal through a G-protein activated second messenger system. Knockout studies in mice suggested that this protein inhibits embryonic oligodendrocyte precursor migration in developing spinal cord. This gene, IL8RB, a gene encoding another high affinity IL8 receptor, as well as IL8RBP, a pseudogene of IL8RB, form a gene cluster in a region mapped to chromosome 2q33-q36.
CXCR2	chemokine (C-X-C motif) receptor 2	The protein encoded by this gene is a member of the G-protein-coupled receptor family. This protein is a receptor for interleukin 8 (IL8). It binds to IL8 with high affinity, and transduces the signal through a G-protein activated second messenger system. This receptor also binds to chemokine (C-X-C motif) ligand 1 (CXCL1/MGSA), a protein with melanoma growth stimulating activity, and has been shown to be a major component required for serum-dependent melanoma cell growth. This receptor mediates neutrophil migration to sites of inflammation. The angiogenic effects of IL8 in intestinal microvascular endothelial cells are found to be mediated by this receptor. Knockout studies in mice suggested that this receptor controls the positioning of oligodendrocyte precursors in developing spinal cord by arresting their migration. This gene, IL8RA, a gene encoding another high affinity IL8 receptor, as well as IL8RBP, a pseudogene of IL8RB, form a gene cluster in a region mapped to chromosome 2q33-q36. Alternatively spliced variants, encoding the same protein, have been identified.
CXCL2	chemokine (C-X-C motif) ligand 2	
CXCL3		

PPBP	pro-platelet basic protein (chemokine (C-X-C motif) ligand 7)	The protein encoded by this gene is a platelet-derived growth factor that belongs to the CXC chemokine family. This growth factor is a potent chemoattractant and activator of neutrophils. It has been shown to stimulate various cellular processes including DNA synthesis, mitosis, glycolysis, intracellular cAMP accumulation, prostaglandin E2 secretion, and synthesis of hyaluronic acid and sulfated glycosaminoglycan. It also stimulates the formation and secretion of plasminogen activator by synovial cells.

Module 5 : Notch Signaling

Gene Name	Official Full Name	NCBI Gene Summary
NOTCH1	notch 1	This gene encodes a member of the Notch family. Members of this Type 1 transmembrane protein family share structural characteristics including an extracellular domain consisting of multiple epidermal growth factor-like (EGF) repeats, and an intracellular domain consisting of multiple, different domain types. Notch family members play a role in a variety of developmental processes by controlling cell fate decisions. The Notch signaling network is an evolutionarily conserved intercellular signaling pathway which regulates interactions between physically adjacent cells. In Drosophilia, notch interaction with its cell-bound ligands (delta, serrate) establishes an intercellular signaling pathway that plays a key role in development. Homologues of the notch-ligands have also been identified in human, but precise interactions between these ligands and the human notch homologues remain to be determined. This protein is cleaved in the trans-Golgi network, and presented on the cell surface as a heterodimer. This protein functions as a receptor for membrane bound ligands, and may play multiple roles during development.
NOTCH4	notch 4	This gene encodes a member of the Notch family. Members of this Type 1 transmembrane protein family share structural characteristics including an extracellular domain consisting of multiple epidermal growth factor-like (EGF) repeats, and an intracellular domain consisting of multiple, different domain types. Notch family members play a role in a variety of developmental processes by controlling cell fate decisions. The Notch signaling network is an evolutionarily conserved intercellular signaling pathway which regulates interactions between physically adjacent cells. In Drosophilia, notch interaction with its cell-bound ligands (delta, serrate) establishes an intercellular signaling pathway that plays a key role in development. Homologues of the notch-ligands have also been identified in human, but precise interactions between these ligands and the human notch homologues remain to be determined. This protein is cleaved in the trans-Golgi network, and presented on the cell surface as a heterodimer. This protein functions as a receptor for membrane bound ligands, and may play a role in vascular, renal and hepatic development. This gene may be associated with susceptibility to schizophrenia in a small portion of cases. An alternative splice variant has been described but its biological nature has not been determined.
DLL1	delta-like 1	DLL1 is a human homolog of the Notch Delta ligand and is a member of the delta/serrate/jagged family. It plays a role in mediating cell fate decisions during hematopoiesis. It may play a role in cell-to-cell communication.
DLL4	delta-like 4	This gene is a homolog of the Drosophila delta gene. The delta gene family encodes Notch ligands that are characterized by a DSL domain, EGF repeats, and a transmembrane domain.
MFNG	MFNG O-fucosylpeptide 3-beta-N-acetylglucosaminyltransferase	This gene is a member of the fringe gene family which also includes radical and lunatic fringe genes. They all encode evolutionarily conserved secreted proteins that act in the Notch receptor pathway to demarcate boundaries during embryonic development. While their genomic structure is distinct from other glycosyltransferases, fringe proteins have a fucose-specific beta-1,3-N-acetylglucosaminyltransferase activity that leads to elongation of O-linked fucose residues on Notch, which alters Notch signaling.

Module 6 : RAMPs-GPCR Modulation

Gene Name	Official Full Name	NCBI Gene Summary
RAMP2	receptor (G protein-coupled) activity modifying protein 2	The protein encoded by this gene is a member of the RAMP family of single-transmembrane-domain proteins, called receptor (calcitonin) activity modifying proteins (RAMPs). RAMPs are type I transmembrane proteins with an extracellular N terminus and a cytoplasmic C terminus. RAMPs are required to transport calcitonin-receptor-like receptor (CRLR) to the plasma membrane. CRLR, a receptor with seven transmembrane domains, can function as either a calcitonin-gene-related peptide (CGRP) receptor or an adrenomedullin receptor, depending on which members of the RAMP family are expressed. In the presence of this (RAMP2) protein, CRLR functions as an adrenomedullin receptor. The RAMP2 protein is involved in core glycosylation and transportation of adrenomedullin receptor to the cell surface.
RAMP3	receptor (G protein-coupled) activity modifying protein 3	The protein encoded by this gene is a member of the RAMP family of single-transmembrane-domain proteins, called receptor (calcitonin) activity modifying proteins (RAMPs). RAMPs are type I transmembrane proteins with an extracellular N terminus and a cytoplasmic C terminus. RAMPs are required to transport calcitonin-receptor-like receptor (CRLR) to the plasma membrane. CRLR, a receptor with seven transmembrane domains, can function as either a calcitonin-gene-related peptide (CGRP) receptor or an adrenomedullin receptor, depending on which members of the RAMP family are expressed. In the presence of this (RAMP3) protein, CRLR functions as an adrenomedullin receptor.
VIPR1	vasoactive intestinal peptide receptor 1	This gene encodes a receptor for vasoactive intestinal peptide, a small neuropeptide. Vasoactive intestinal peptide is involved in smooth muscle relaxation, exocrine and endocrine secretion, and water and ion flux in lung and intestinal epithelia. Its actions are effected through integral membrane receptors associated with a guanine nucleotide binding protein which activates adenylate cyclase.
CALCRL	calcitonin receptor-like	

Module 7 : JUN-FOS Signaling

Gene Name	Official Full Name	NCBI Gene Summary
JUNB	jun B proto-oncogene	
JUND	jun D proto-oncogene	The protein encoded by this intronless gene is a member of the JUN family, and a functional component of the AP1 transcription factor complex. It has been proposed to protect cells from p53-dependent senescence and apoptosis. Alternate translation initiation site usage results in the production of different isoforms.
FOSB	FBJ murine osteosarcoma viral oncogene homolog B	The Fos gene family consists of 4 members: FOS, FOSB, FOSL1, and FOSL2. These genes encode leucine zipper proteins that can dimerize with proteins of the JUN family, thereby forming the transcription factor complex AP-1. As such, the FOS proteins have been implicated as regulators of cell proliferation, differentiation, and transformation. Alternatively spliced transcript variants encoding different isoforms have been found for this gene.
FOSL1	FOS-like antigen 1	The Fos gene family consists of 4 members: FOS, FOSB, FOSL1, and FOSL2. These genes encode leucine zipper proteins that can dimerize with proteins of the JUN family, thereby forming the transcription factor complex AP-1. As such, the FOS proteins have been implicated as regulators of cell proliferation, differentiation, and transformation.

Module 8

Gene Name	Official Full Name	NCBI Gene Summary

KRT15	keratin 15	The protein encoded by this gene is a member of the keratin gene family. The keratins are intermediate filament proteins responsible for the structural integrity of epithelial cells and are subdivided into cytokeratins and hair keratins. Most of the type I cytokeratins consist of acidic proteins which are arranged in pairs of heterotypic keratin chains and are clustered in a region on chromosome 17q21.2.
USHBP1	Usher syndrome 1C binding protein 1	
FAM107A	family with sequence similarity 107, member A	
C10orf10	chromosome 10 open reading frame 10	The expression of this gene is induced by fasting as well as by progesterone. The protein encoded by this gene contains a t-synaptosome-associated protein receptor (SNARE) coiled-coil homology domain and a peroxisomal targeting signal. Production of the encoded protein leads to phosphorylation and activation of the transcription factor ELK1
