# Supplementary material for: A High-Dimensional, Deep-Sequencing Study of Lung Adenocarcinoma in Female Never-Smokers
Source: PLoS One. 2013 Feb 6;8(2):e55596. doi: 10.1371/journal.pone.0055596 (PMC3566005; doi:10.1371/journal.pone.0055596)
Supplement: File S7 — Comparison with public expression data on NSCLC. (PDF) [file pone.0055596.s007.pdf]

Supplemental File 7. Comparison with public expression data on NSCLC

A number of microarray-based studies have been carried out on lung cancer. A logical question would be how our data compares with these previous studies. From public gene expression data on lung cancer in GEO and ArrayExpress databases, we identified 12 data sets each with both tumor and normal specimens.

To avoid the complications from meta-analysis (different platforms, labs, protocols, etc.), comparisons were done at the gene set level, not on expression values of individual genes. All array data were normalized with the *RMA* method, and DEGs were obtained by using the *limma* package in R with the adjusted p-value of 0.001. The DEG sets from various experiments were clustered by hierarchical method. The distance metric was the hyper-geometric p-value of testing co-membership between two gene sets.

Clustering of public experiments (see below) indicates that our result is closest to that of GSE19804 which profiled never-smoker female lung adenocarcinoma in Taiwan. The fact that a study on a patient with virtually identical demographic characteristics yielded results most similar to ours provides an additional validation of the current study.

Patient grouping according to the mutation subtype is critical for selecting treatment methods. GSE31210 contains mutation information for EGFR, KRAS, ALK genes that are established as key biomarkers for subtyping lung adenocarcinoma. Comparing up and down DEG sets, our patient group was shown to be closest to the no-mutation group in GSE31210. Again, this provides further validation of our study given that all of our patients belong to no-mutation group. In sum, comparison with publicly available data not only confirmed the validity of our data set, but also raises an important and testable hypothesis that never-smoker female adenocarcinoma patients constitute a distinctive group from those with EGFR, KRAS, and ALK mutations.

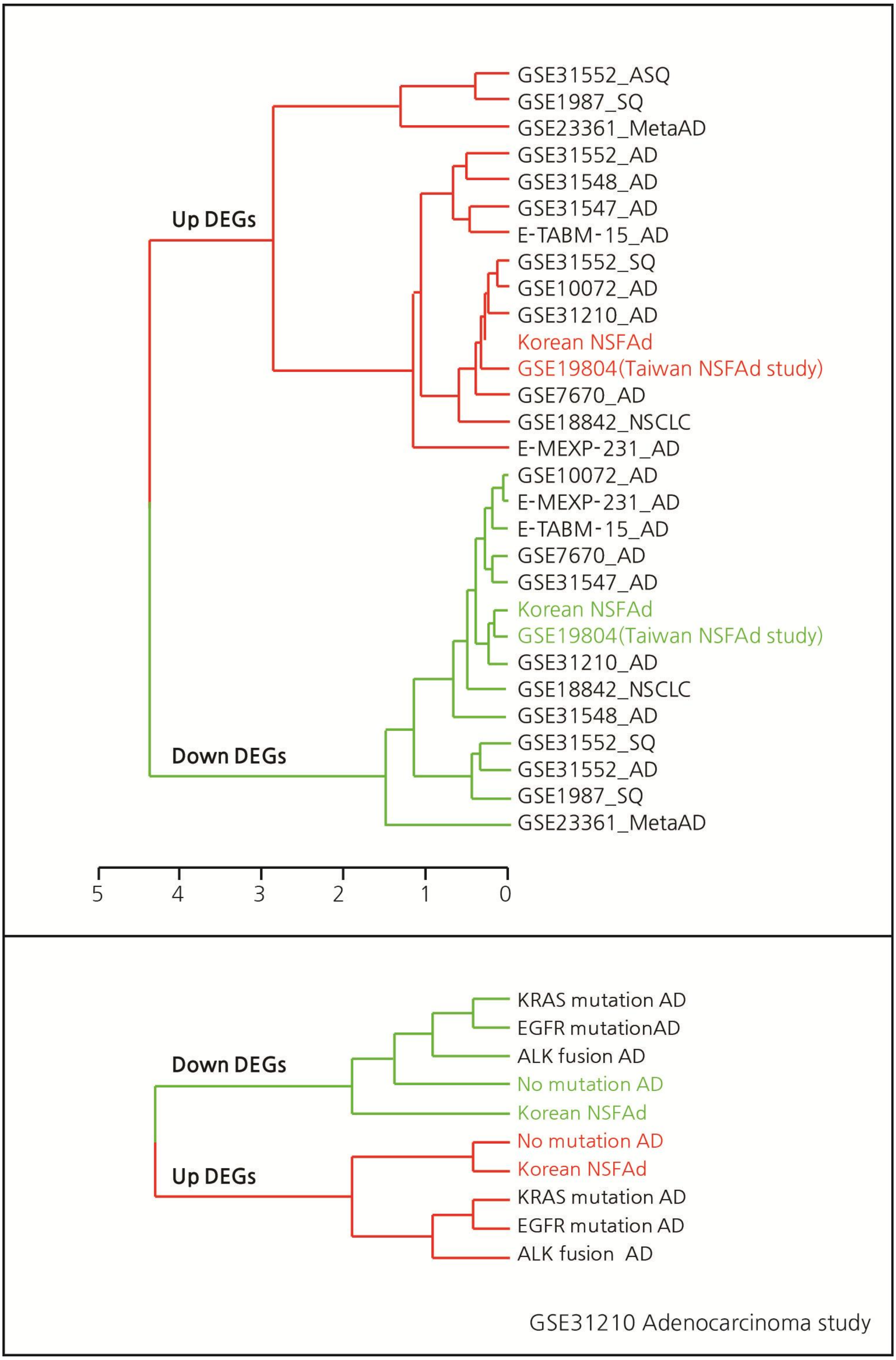
GSE31552_ASQ
GSE1987_SQ
GSE23361_MetaAD
GSE31552_AD
GSE31548_AD
GSE31547_AD
E-TABM-15_AD
GSE31552_SQ
GSE10072_AD
GSE31210_AD
Korean NSFAd
GSE19804(Taiwan NSFAd study)
GSE7670_AD
GSE18842_NSCLC
E-MEXP-231_AD
Up DEGs
GSE10072_AD
E-MEXP-231_AD
E-TABM-15_AD
GSE7670_AD
GSE31547_AD
Korean NSFAd
GSE19804(Taiwan NSFAd study)
GSE31210_AD
GSE18842_NSCLC
GSE31548_AD
GSE31552_SQ
GSE31552_AD
GSE1987_SQ
GSE23361_MetaAD
Down DEGs
5
4
3
2
1
0
KRAS mutation AD
EGFR mutationAD
ALK fusion AD
No mutation AD
Korean NSFAd
Down DEGs
No mutation AD
Korean NSFAd
KRAS mutation AD
EGFR mutation AD
ALK fusion AD
Up DEGs
GSE31210 Adenocarcinoma study
