# Supplementary material for: A High-Dimensional, Deep-Sequencing Study of Lung Adenocarcinoma in Female Never-Smokers
Source: PLoS One. 2013 Feb 6;8(2):e55596. doi: 10.1371/journal.pone.0055596 (PMC3566005; doi:10.1371/journal.pone.0055596)
Supplement: File S8 — Supplemental information; Supplemental materials, methods, figures, tables and references. (PDF) [file pone.0055596.s008.pdf]

Table of Contents

Supplemental Materials and Methods 3
1. Patient Samples and Experimental Methods........ 3
2. Mutation Analysis (Exome-seq data)........ 5
    2.1 Sequence alignment and mutation calling 5
    2.2 Validation of somatic mutations........ 6
3. Transcriptome Analysis (RNA-Seq data)........ 6
    3.1 RNA quantification and differential expression analysis........ 6
    3.2 Detection of fusion transcripts........ 7
    3.3 Small RNA sequencing analysis 9
4. Array-CGH and MeDIP-Seq Data Analyses........ 10
    4.1 Array-CGH data analysis for copy number variations (CNVs) 10
    4.2 MeDIP-Seq data analysis for DNA methylation........ 11
5. Network and Functional Analysis........ 11
    5.1 Functional analysis of DEGs, somatic mutations, and fusion genes 12
    5.2 Network analysis of DEGs, somatic mutations, and fusion genes 12

Supplemental Figures 13
Figure S1. Overview of experimental data and analysis........ 13
Figure S2. Circos plots for each patient........ 14
Figure S3. Classification of somatic mutations. 15
Figure S4. Workflow for analyzing small RNA-Seq data. 16
Figure S5. Length distribution of small RNA-Seq data (in RPM). 17
Figure S6. MicroRNA-target relations with inversely correlated expression........ 18
Figure S7. CNV plot by chromosome for each patient........ 19
Figure S8. Genomic regions with copy number gains or losses. 22
Figure S9. Statistical enrichment test of functional terms in IPA. 23
Figure S10. Overall PPI network of 1536 genes........ 24
Figure S11. Network modules from MCODE. 25
Figure S12. Functional grouping of MCODE modules in 3 broad categories........ 26

Supplemental Tables 27
Table S1. Characteristics of Patients........ 27
Table S2. Summary statistics for mapping exome-seq data 28
Table S3. List of experimentally confirmed somatic mutations 29
Table S4. RNA-Seq data mapping summary 31
Table S5. Number of differentially regulated genes and isoforms 32
Table S6. Primer sequences for detecting fusion transcripts 33
Table S7. Mapping statistics of small RNA-Seq data........ 34
Table S8. List of differentially expressed microRNAs........ 35
Table S9. Inversely correlated microRNA-target relations........ 36
Table S10. Number of genes with copy number variations outside 1.62-2.46 for each patient........ 41
Table S11. Chromosomal gains and losses in previously published papers and in present study........ 42
Table S12. Correlations between copy number variations and RNA expression 43
Table S13. Correlations between DNA methylation and RNA expression........ 47
Table S14. Mapping Summary for MeDIP-Seq data........ 50
Table S15. Top scoring gene functions from Ingenuity Pathway Analysis (IPA)........ 51

Supplemental References 52

Supplemental Files

File S1. Complete pathological reports
File S2. List of differentially expressed genes and isoforms (DEGs and DEIs)
File S3. MicroRNA expression plot for tumor and normal tissues
File S4. List of CNV genes in at least 3 out of 6 patients
File S5. List of DMR genes
File S6. Gene lists with brief summaries for somatic mutations and network modules
File S7 Comparison with public expression data on NSCLC

Supplemental Materials and Methods

1. Patient Samples and Experimental Methods

Patient sample description

Patient samples were obtained from lung cancer patients who had undergone curative surgery. All samples were collected with the written informed consent from patient and prior approval of the institutional review board (Samsung Medical Center-Institutional Review Board; approval number 2010-08-063-006). All cases were first-time (i.e. non-recurrent) lung cancers. None of the patients had distant metastasis or family history of lung cancer (see Table S1).

DNA and RNA preparations for NGS

Genomic DNA and total RNA were extracted from single surgical sample of each patient which contained primary lung adenocarcinoma tumor and adjacent noncancerous lung tissue. The samples were snap-frozen or were stored in RNAlater RNA Stabilization (Qiagen, Germany) solution. Total RNA was isolated using miRNeasy Mini Kit columns according to the manufacturer's protocol (Qiagen). The quality of RNA, which was determined by the RNA integrity number (RIN), was assessed by an Agilent 2100 Bioanalyzer using the RNA 6000 Nano Chip (Agilent Technologies, Amstelveen, The Netherlands). All samples showed RNA Integrity Numbers > 7.0. The quantity of total RNA was determined by NanoDrop (ND-8000) Spectrophotometer (Thermo Scientific, Wilmington, DE, USA) and by Ribogreen analysis (Invitrogen, Carlsbad, CA, USA). Genomic DNA was extracted from the tissues according to standard protocols (proteinase K digestion, phenol/chloroform extraction, and ethanol precipitation). Detailed protocols are available upon request.

Exome -seq sample preparation and sequencing

We prepared and sequenced the exome using the Solexa sequencing technology platform (Genome Analyzer IIx, Illumina, San Diego, CA) following the manufacturer's instructions. We sheared 3 ug of genomic DNA using the Covaris System. The fragmented DNA with an average size of 150 bp was end-repaired using T4 DNA polymerase and Klenow polymerase, and Illumina paired-end adaptor oligonucleotides were ligated to the ends. We analyzed the ligation mixture by electrophoresis on an agarose gel and isolated fragments measuring 200-

250 bp. The purified DNA library was hybridized with the SureSelect Human All Exons V1 probe set (Agilent Technologies) to capture 44 Mb of targeted exons following the manufacturer's instructions. We prepared a Genome Analyzer IIx paired-end flowcell using a captured exome library. Clusters of PCR colonies were then sequenced on the Genome Analyzer IIx platform using the manufacturer's recommended protocols. Detailed protocols are available upon request.

RNA-seq sample preparation and sequencing

Total RNA was prepared in accordance with Illumina's sample preparation protocol for PE sequencing of mRNA. mRNA was isolated from total RNA preparations using poly-T oligo-attached magnetic beads and fragmented with the fragmentation buffer in the mRNA Sequencing Sample Prep Kit (Illumina). Subsequent steps were identical to exome sequencing, except that the size of the purified fragments from the agarose gel was 300 bp. Detailed protocols are available upon request.

Small RNA-seq sample preparation and sequencing

Purified total RNA was processed using the Illumina small RNA sample preparation kit following the manufacturer's protocol. Small RNA fragments, 22 to 30 nucleotides long, were prepared from a PAGE gel and reverse transcribed. Sequencing was performed using the Illumina Genome Analyzer IIx, with 36-bp single read sequencing.

Methylated DNA immunoprecipitation-sequencing (MeDIP-seq) sample preparation and sequencing

Methylated CpG island-associated DNA was prepared using methylation-specific antibody against 5-methylcytidine (Eurogentec, Seraing, Belgium) and Dynabeads with M-280 sheep antibody to mouse IgG (Invitrogen Dynal, Oslo, Norway). Immunoprecipitated DNA was processed for high throughput sequencing using the Illumina ChIP-Seq Sample Prep Kit. For sequencing, a 200 bp-insert sized library was prepared from agarose gel electrophoresis and size selection. Sequencing was performed using the Illumina Genome Analyzer IIx, and 50 bp-long single reads were generated. Detailed protocols are available upon request.

Genomic-PCR, RT-PCR and Sanger sequencing

Somatic mutations and fusion transcript candidates were validated by PCR amplification and Sanger sequencing. Typically, 20 ng of genomic DNA and cDNA equivalent to 40 ng of input total RNA were subjected to PCR amplification using Platinum Taq DNA polymerase (Invitrogen, Carlsbad, CA). Detailed conditions for PCR are available upon request. The PCR products were visually examined by agarose gel electrophoresis, purified using the PCR purification kit (Qiagen) and subjected to conventional Sanger sequencing. When subcloning was necessary, the TOPcloner™ TA kit (Enzynomics, South Korea) was used, following the manufacturer's protocol.

2. Mutation Analysis (Exome-seq data)

2.1 Sequence alignment and mutation calling

Single-end 75-bp reads from the exome-seq were mapped to the human reference genome (build 19) using the Burrows-Wheeler Aligner software (*BWA*, v0.5.9) [1]. An average of 34 million reads was generated for each sample, of which 59% aligned to the targeted regions. Overall, 79% of the targeted regions were covered. Aligned reads were processed using *Picard* software (http://picard.sourceforge.net, v1.42) to remove PCR duplicates. The Genome Analysis Toolkit (*GATK*, v1.0.5506) was then used to perform local realignment around indels and to recalibrate base quality scores to produce a more accurate alignment for each sample [2]. Table S2 shows the statistical summary for mapping the exome-seq data for each patient.

Mutation calling was achieved using *JointSNVmix* software (v0.6.2) to take advantage of the paired nature of the tumor and normal samples. *JointSNVmix* provides the posterior probability of somatic mutation ('somatic probability') as the reliability index. Our initial attempt with *VarScan* software (v1.0) produced numerous cases of false positives in subsequent validation experiments [3]. Validation experiments showed that the somatic probabilities below 0.990 were mostly false. The GenomicAnnotator module in *GATK* and *SIFT* were used to annotate the variants in terms of coding potential and to predict the functional effects of the coding variants [4]. The classification of somatic mutations with respect to gene structure and function is illustrated in Figure S3 and further described in File S6.

2.2 Validation of somatic mutations

Using the cutoff values of 0.990 and 0.999, we obtained 335 and 189 candidates for somatic mutation, respectively. Among 189 candidates with a somatic probability over 0.999, we selected mutations in CDS, UTR, and noncoding exons for experimental validation.

Conventional Sanger sequencing following PCR amplification confirmed 45 valid mutations with 55 false predictions and 3 ambiguous cases out of a total of 103 cases. The prediction accuracy was below 44%, even with the rigorous criterion of somatic probability over 0.999. Testing additional candidates with a somatic probability between 0.990-0.999, we obtained just one positive out of 9 cases. Candidates for somatic probability below 0.990 turned out to be false in all 21 tested cases. We found only one additional confirmed mutation predicted by *VarScan* only. This low success ratio may be due to contamination by normal cells and/or heterogeneity of the tumor samples. In total, we identified 47 somatic mutations (see Table S3). The sequences of oligonucleotide primers used for PCR and sequencing are available upon request.

3. Transcriptome Analysis (RNA-Seq data)

3.1 RNA quantification and differential expression analysis

We used the *NEUMA* program to quantify transcript abundance. Since *NEUMA* maps sequence reads to the established transcriptome, identification of novel isoforms is not supported. However, the *NEUMA* program's primary merit is the accuracy of quantification, as its algorithm mimics the real-time PCR process (agreement ratio over 0.9 with experimental data).

Mapping and quantification. Mapping of the paired end sequences was carried out using *NEUMA* (version v1.1.1) and *Bowtie* (version 0.12.7) on the hg19 Refseq RNA sequences (release 47) downloaded from the UCSC Genome Browser (http://genome.ucsc.edu). For 78-bp paired-end data, *Bowtie* was run with the –v 0 –a −maxins 600 option, i.e., retrieving all aligned positions, allowing only perfect matches and cDNA fragment sizes of 79-600 bp. The same range of cDNA fragment sizes was used to calculate the normalization factors for each transcript. We mapped all reads to 36,475 RNA isoforms corresponding to 22,744 genes within hg19 RefSeq RNA sequences. A summary of the mapping RNA-Seq data is provided in Table S4. Then we selected the abundance estimates of only 31,841 mRNA isoforms

(20,990 coding genes), taking into consideration the poly-A selection step in the RNA-Seq experimental procedure. Hierarchical clustering of RNA reads revealed two separate groups of normal and tumor tissues in a manner consistent across all 6 patients, validating the functional significance of the gene expression profiles. The RNA-Seq data and results of *NEUMA* quantification were deposited into the GEO database with the accession number of GSE37765.

Identification of differentially-expressed transcripts. We chose to use the *edgeR* program to detect differential expression mainly because it supports an experimental design using paired samples (i.e., tumor-normal pairs from the same person) [5]. However, we were hampered by a number of cases of low-level expression genes or cases of differential expression in only one or two patients. We therefore devised several filtering steps to isolate only the significant differential expression. Four conditions needed to be met for the genes to be selected as differentially expressed genes (DEGs) or differentially expressed isoforms (DEIs) – (i) overall differential expression from the *edgeR* analysis with FDR < 0.001, (ii) a minimum of 3 patients with significant differential expression, as tested by *edgeR* for individual differential expression with FDR < 0.01, (iii) consistent up/down regulation among different patients representing more than a two-fold change, and (iv) significant expression in at least 3 patients to remove genes with large fold changes within the noise expression level (FVKM>2 in either normal or tumor tissue). In total, we selected 1459 genes (543 upregulated and 916 downregulated in tumors) differentially expressed in female NSCLC never-smoker patients. A similar calculation for isoforms yielded 1320 DEIs (460 upregulated and 860 downregulated in tumors). The numbers of DEGs and DEIs according to nPatDE (the number of patients with significant differential expression in *edgeR* with FDR < 0.01) and nPat2fold (the number of patients with greater than 2-fold expression changes) are summarized in Table S5. The full lists of DEGs and DEIs are provided in the File S2.

3.2 Detection of fusion transcripts

We used the *FusionMap* and our own in-house developed program, *FusionScan*, to predict candidate fusion transcripts from the RNA-Seq data. These two programs produce a relatively small number of false positives because they require the fusion boundary to occur inside the sequence reads both in single and paired end data.

FusionMap prediction. The RNA-Seq reads were aligned to the human reference genome (hg19) using *BWA* (version 0.5.9) with default parameters. All unaligned or discordant reads, which accounted for approximately 40% of the total reads, were collected as an input file to *FusionMap*. The parameters were set as follows: the minimal end length of the seed read (α) = 25, the maximal hits of the read end (β) = 1, and the non-canonical splice pattern penalty (G) = 4. In total, *FusionMap* reported 337 candidates for fusion transcripts, 131 of which were tumor-specific (114 normal tissue-specific candidates and 92 common candidates). We manually examined the *BLAT* alignments for tumor-specific candidates to remove reads whose head or tail part was mapped to multiple genomic loci. Among 54 uniquely mapped candidates, we selected 7 inter-chromosomal and 14 intra-chromosomal events as final candidates for experimental validation. Intra-chromosomal cases were further divided into two groups, one with 4 events where fusion reads mapped onto opposite strands and the other with 10 events between non-neighboring genes on the same strand.

FusionScan prediction. Our in-house program is conceptually similar to *FusionMap*, but the detailed logic and filtering procedures are substantially different. Most notably, we limit the candidates to those with genomic breakpoints inside the introns. Thus, the fusion boundary for both head and tail genes matches the respective exon boundaries exactly. Genomic alignment to hg19 was achieved by using the *SSAHA2* (version 2.5.5) [6] program with the '-solexa –skip 6' option. Candidates for fusion transcripts must satisfy several stringent requirements: (i) a minimum alignment length of 25 bp for both head and tail genes, (ii) the fusion boundary matches the exon boundary at the exact position for both head and tail genes, (iii) the fusion loci are not in a repeat region or in a homologous region with a sequence identity of over 85%, (iv) there is consistent chromosomal identity in the non-fusion reads of paired-end data. Reads satisfying all four of the above conditions were called *seed* reads. Setting the minimum seed read count to 2, we obtained 382 fusion candidates, 91 of which were tumor-specific. We further filtered out cases without valid gene symbols and cases with head and tail genes belonging to the same gene family. In an effort to find supporting evidence, we aligned the original RNA-Seq data with the hypothetical fusion transcript to identify reads with partial overlap with either head or tail genes. These additional reads were designated as the *rescued* reads. After manual inspection of read alignments across the fusion boundary, we obtained 19 tumor-specific and 5 normal tissue-specific candidate fusion transcripts. Only tumor-specific cases were subjected to experimental

validation as final candidates.

Experimental Validation. We performed RT-PCR validation experiments followed by Sanger sequencing for 21 fusion candidates from *FusionMap* and 19 candidates from *FusionScan*. In total, we confirmed 19 cases of genuine fusion transcripts (11 common predictions, 6 *FusionScan* only, 2 *FusionMap* only). The list of confirmed fusion transcripts and primer pair sequences are shown in Tables 1 and Table S6. All candidates predicted by both programs turned out to be valid (11 cases). Candidates from *FusionMap* were valid in only 2 out of 10 cases. Candidates from *FusionScan* were valid in only 6 out of 8 cases. Detailed examination revealed that two of the 6 confirmed cases were classified as multi-reads in *FusionMap* and were consequently eliminated in the filtering steps.

3.3 Small RNA sequencing analysis

Computational pipeline. The workflow for analyzing small RNA-Seq data is shown in Figure S4. In this study, we focused on microRNA exclusively, even though our raw sequencing data included other types of small RNAs. Our analysis consisted of four parts: mapping, normalization and quantification, identification of differentially expressed microRNAs (DEmiRs), and identification of microRNAs inversely correlated with DEGs. Sequence reads were mapped with *Bowtie V.0.12.7* using the perfect match option into the known microRNA database (miRBase release 17) after removing the adaptor sequence (5'ATCTCGTATGCCGTCTTCTGCTTG). Table S7 shows the statistics of read mapping for each patient's data. On average, 70% and 76% of the total reads were mapped to the known microRNAs in normal and tumor tissues, respectively. The portion of mapped microRNAs was consistently higher by 5%-6% in tumor samples. The length distribution of reads is shown in Figure S5. Reads in the range of 20-23 bp occupy 87.2% of the total reads, reflecting an abundance of microRNAs among various groups of small RNAs. MicroRNA abundance was quantified by using the *TMM* normalization program [7]. The raw and normalized expression data were uploaded into the GEO database with the accession number of GSE37765.

Differentially expressed microRNAs (DEmiRs) were determined by using a similar process as that described above for mRNA. Four conditions needed to be met to be selected as DEmiRs: (i) overall differential expression from the *edgeR* analysis with FDR < 0.001, (ii)

a minimum of 3 patients with significant differential expression as tested by *edgeR* for individual differential expression with FDR < 0.01, (iii) a greater than two-fold consistent change in up/down regulation among different patients, and (iv) *significant* expression in at least 3 patients. For the last condition, we examined the distribution of normalized microRNA expression values in each patient and determined the upper 25th percentile as significantly expressed microRNA. Thus, the larger of the expression values in either tumor or normal tissues must be within the upper 25% in at least 3 patients to be eligible as a DEmiR. We found 40 DEmiRs (23 upregulated and 17 downregulated microRNAs) in cancer, satisfying these conditions (Table S8).

To identify additional functionally important microRNAs, we further investigated the inversely correlated expression between microRNAs and target genes. Targets were limited to the DEGs for this analysis. For DEmiR-DEG relations, we used both the validated and predicted microRNA-target relations. Validated target relations were obtained by merging the miRecords (version 3) [8], miRTarBase (version 2.5) [9], TarBase (version 5) [10] and miRWalk (last updated November, 2011) [11] databases that collected published results from the literature, and the predicted targets were obtained using TargetScan (version 6) [12]. Using a Pearson correlation cutoff of -0.5 and a P-value cutoff of 0.05, we identified 151 inversely correlated relations among (14 validated and 137 predicted). To explore inversely correlated 'non-DEmiR'-DEG relations, we only used the validated targets to reduce false positives. Using the same cutoffs and an additional requirement of a two-fold change in microRNA expression, we obtained 53 inversely correlated relations. In total, we obtained 204 inversely correlated relations between 31 microRNAs and 165 DEGs (Table S9). We also illustrated the inversely correlated relations in the microRNA-centric network as shown in Figure S6.

Overall, we identified 40 DEmiRs and 13 additional inversely correlated microRNAs that may play important roles in lung cancer development. The expression plot of these functionally important microRNAs in each patient is provided in File S3.

4. Array-CGH and MeDIP-Seq Data Analyses

4.1 Array-CGH data analysis for copy number variations (CNVs)

Array comparative genomic hybridization (CGH) experiments were performed using the Agilent 1M chip with a gender-matched normal human DNA sample as the universal control.

We subtracted the background intensity from the total spot intensity. To remove systematic biases in the chip assay, within-slide normalization was applied using Lowess normalization for log2 transformed data. The copy number plot for normal samples revealed that none of the normal samples showed any significant copy number alteration. The copy number gain or loss [i.e., $\log_2$ (tumor/normal)] was plotted by the chromosome number for each patient in Figure S7. Five out of six patients showed significant copy number alterations in tumor samples.

We used the circular binary segmentation method (*CBS*) to detect statistically significant somatic copy number alterations. A copy number gain or loss beyond $\log_2$ (tumor/normal) = ±0.3 (corresponding to the range outside 1.62-2.46 copies) was recorded for each patient. Figure S8 shows the copy number-altered regions in all six patients. Table S10 shows the summary statistics of copy number-altered genes and regions in each patient. Genes whose copy number was altered in at least three patients are provided in File S4.

4.2 MeDIP-Seq data analysis for DNA methylation

Single-end reads from MeDIP-seq were mapped to the human reference genome (build 19) using the alignment software *Eland* (version 2) using default parameters. The reads with base quality less than 20 and redundant reads were removed from further analysis. The statistics for the mapping of MeDIP reads is shown in Table S14. The number of unique reads for each patient was about 15.4 million (37.6%) on average.

DNA methylation patterns represent a potentially valuable biomarker in various types of cancer. We identified the differentially methylated regions (DMRs) using the *edgeR* program with an FDR cutoff value of 0.05. All aligned unique reads were extended to 200 bp in length to compensate for the difference in read length and to incorporate nearby potential methylation sites. We did not use the methylation level as an additional cutoff for MeDIP-Seq data. In total, we found 558 DMRs, almost 75% of which were in the promoter or 5’ UTR regions. The list of DMRs with genome annotation (from the UCSC genome browser database for hg19) is provided in File S5.

5. Network and Functional Analysis

5.1 Functional analysis of DEGs, somatic mutations, and fusion genes

We obtained 1,536 genes of potential functional importance by collecting 1,459 DEGs, 46 somatic mutations, and 37 fusion genes. Several genes with somatic mutations (*EML1*, *P2RY1*, *PRG4*, *TUBB1*) or genes involved in fusions (*GRHL2*, *RET*, *LAPTM4B*, *LRRC36*) were also differentially expressed; however, the causal relationship was not explored in this study. Ingenuity Pathway Analysis (IPA) software was used to look for statistical enrichment of functional terms in these 1,536 genes. The Benjamini-Hochberg multiple test correction was used with other options at the default setting. A bar plot of significant terms is shown in Figure S9. Top scoring functions from IPA are shown in Table S15.

5.2 Network analysis of DEGs, somatic mutations, and fusion genes

The molecular network for 1,536 genes was constructed with the *MIMI* (http://mimi.ncibi.org/MimiWeb/main-page.jsp, the last update on Apr. 29, 2010) protein-protein interaction (PPI) database. Figure S10 shows the overall PPI network created with the *MONGKIE* visualization software developed at our own center (http://mongkie.org, version 0.1Alpha).

To identify network modules of coherent function, we applied the *MCODE* (http://baderlab.org/Software/MCODE, version 1.32) program to find densely connected network components. We used the default parameters, except for the node score cutoff value of 0.5. We obtained the 8 network modules shown in Figure S11. The full list of genes is provided in File S6 with brief descriptions. Several modules from *MCODE* were involved in related functions. We were able to group the network modules into three broad functional categories as shown in Figure S12. Further analysis for biological meaning and pathway modeling is described in the main text.

Supplemental Figures

Figure S1. Overview of experimental data and analysis.

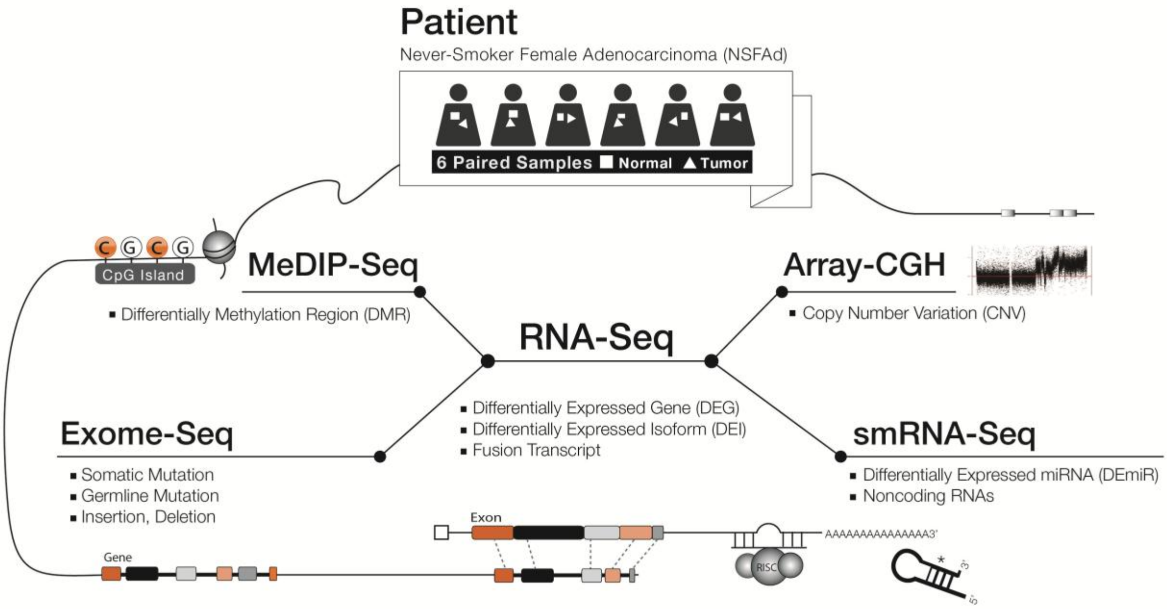

Figure S2. Circos plots for each patient.

The information content and color scheme are identical to Fig. 1 that merged results from six patients.

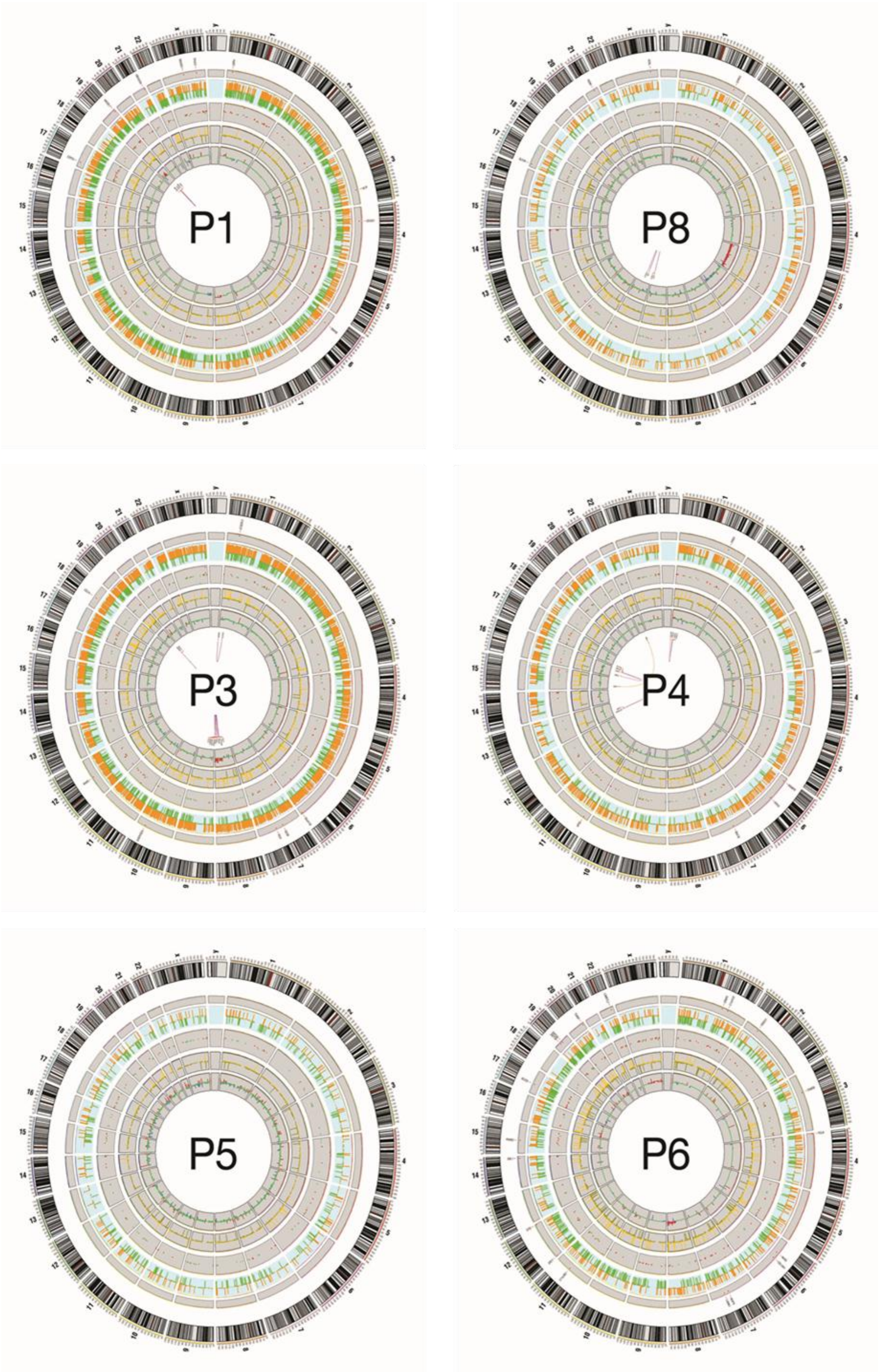

Figure S3. Classification of somatic mutations.

The distribution was obtained for 189 somatic mutations predicted by *JointSNVmix* with somatic probability > 0.999.

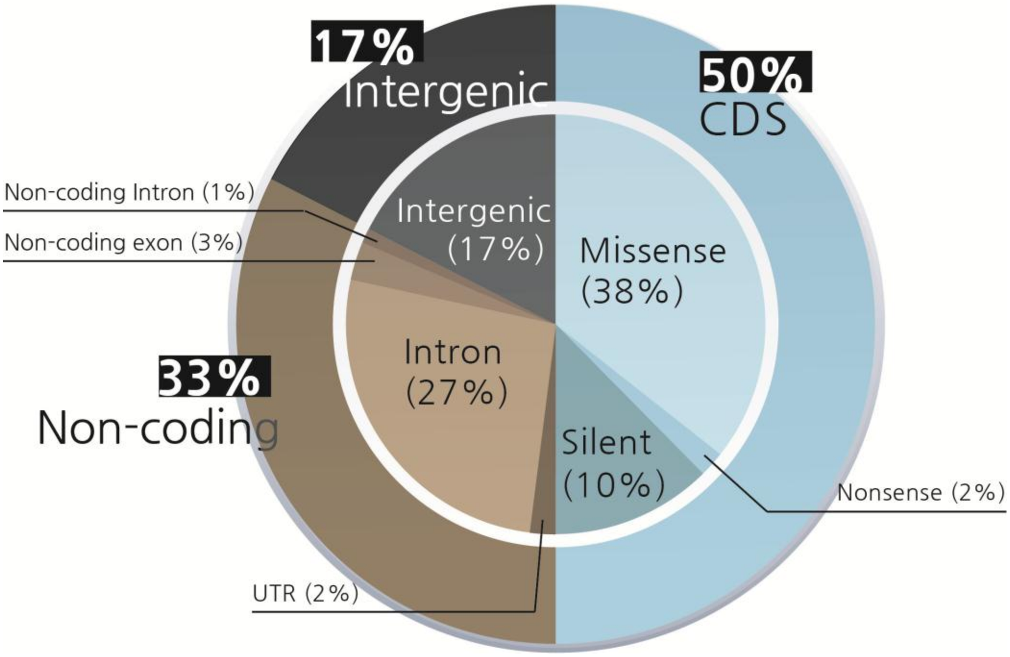

Figure S4. Workflow for analyzing small RNA-Seq data.

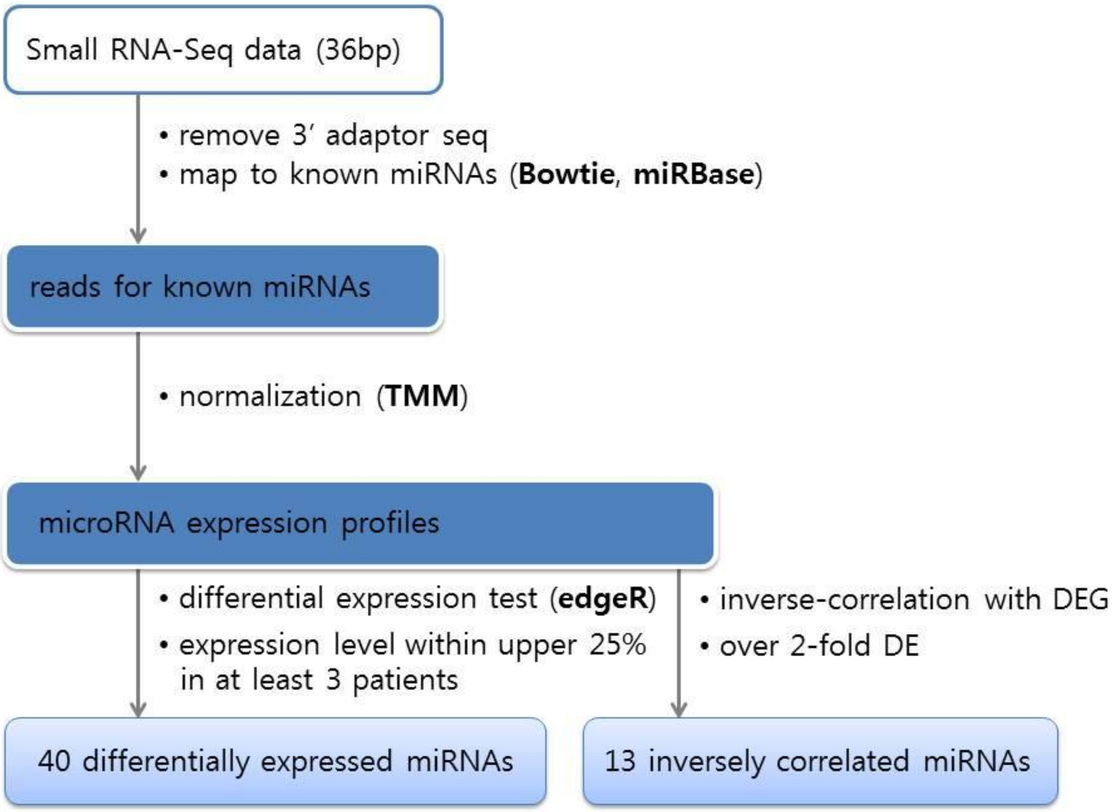

Figure S5. Length distribution of small RNA-Seq data (in RPM).
Read counts were normalized to the reads_per_million (RPM) sequences.

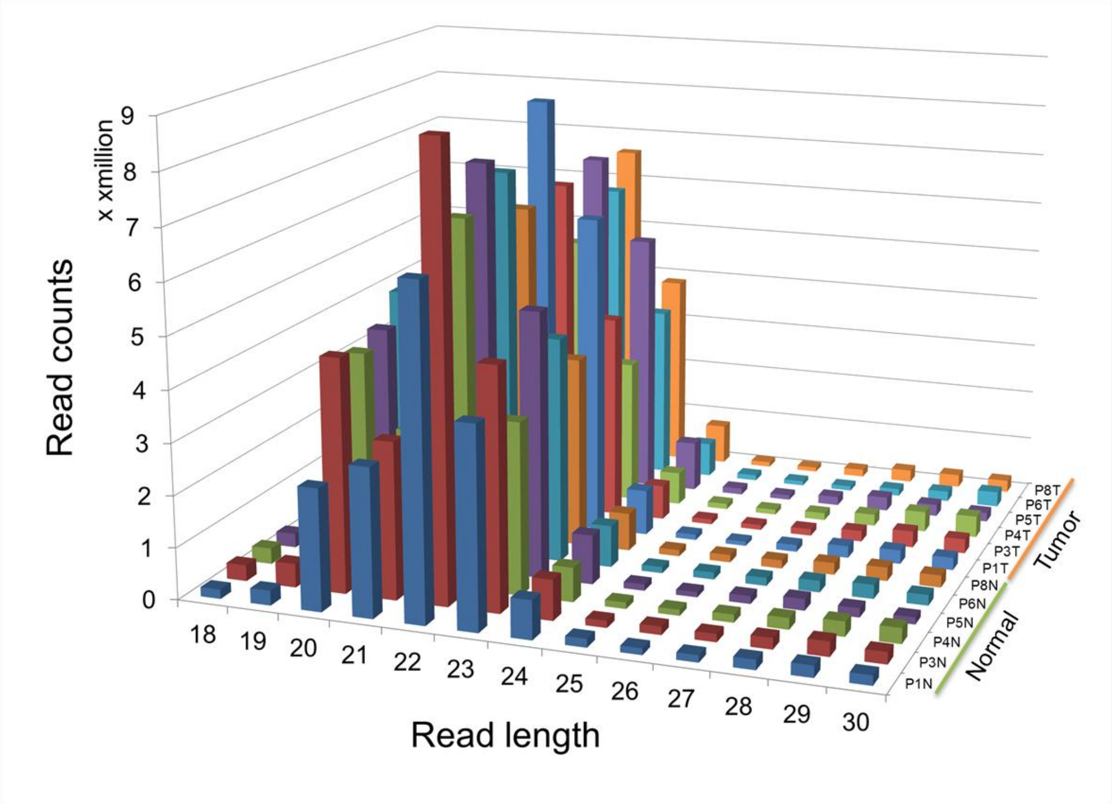

Figure S6. MicroRNA-target relations with inversely correlated expression.
We show the DemiR-DEG relations with the Pearson correlation coefficient < -0.5 and P-value < 0.05. Validated and predicted relations are indicated in solid and dotted lines, respectively. The node color indicates the gene expression level, where red color represent up-regulation in cancer. Triangles are DEmiRs. For convenience, we placed the up-regulated microRNAs in cancer in the left side.

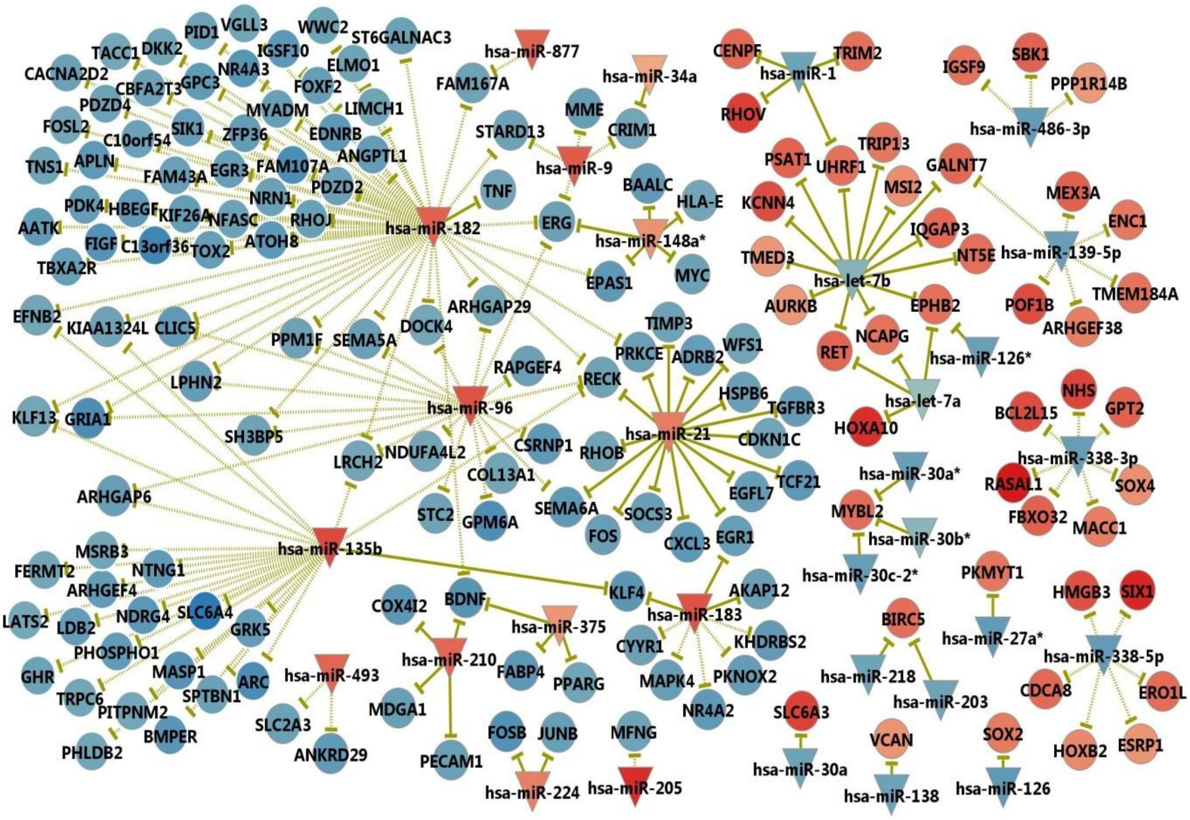

Figure S7. CNV plot by chromosome for each patient.
Copy numbers were plotted in log ratio, i.e., $\log_2$ (tumor/normal).

a

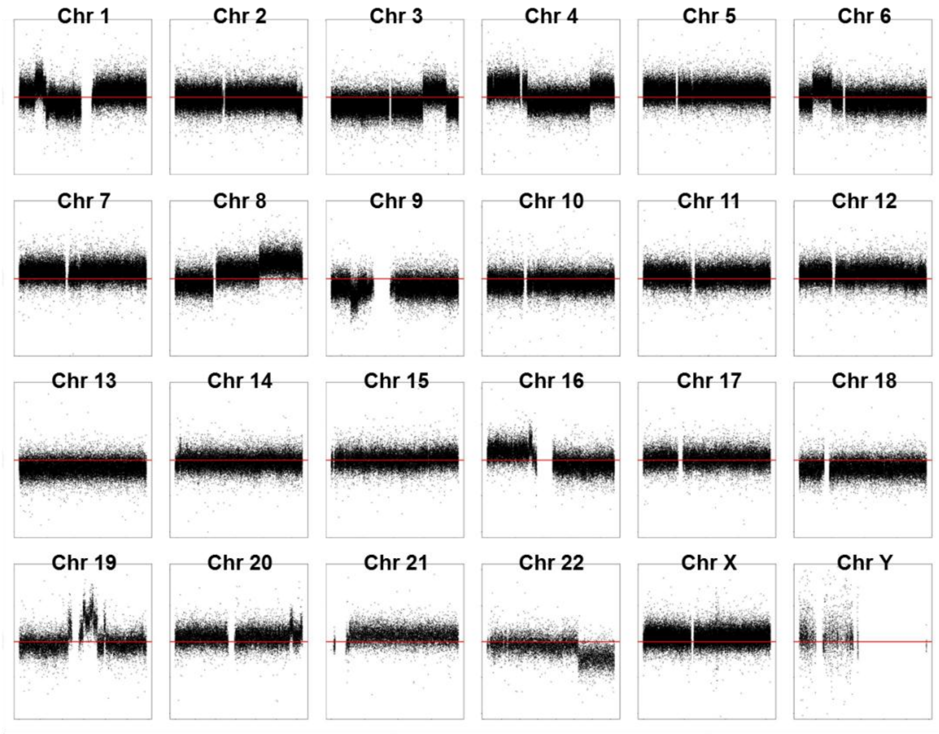

Patient 1

b

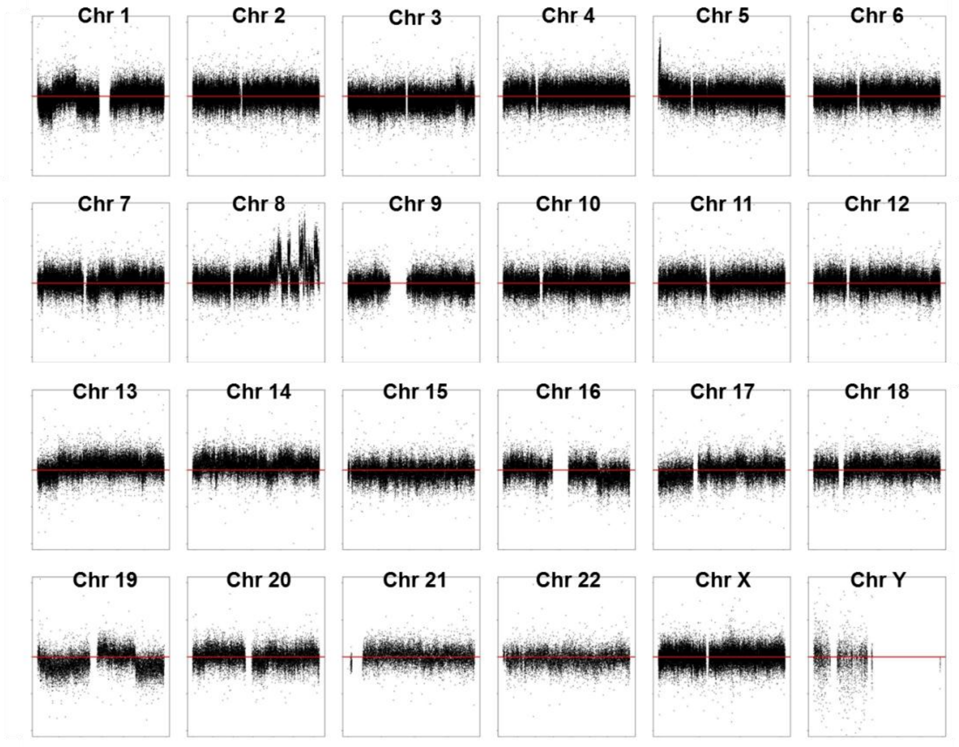

Patient 3

c

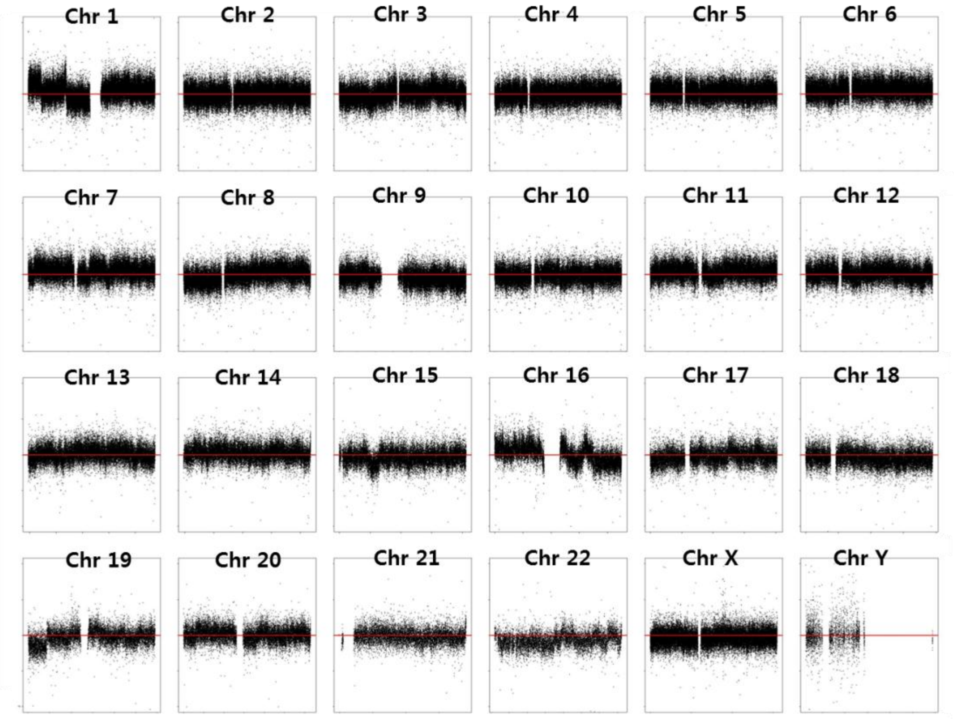

Patient 4

d

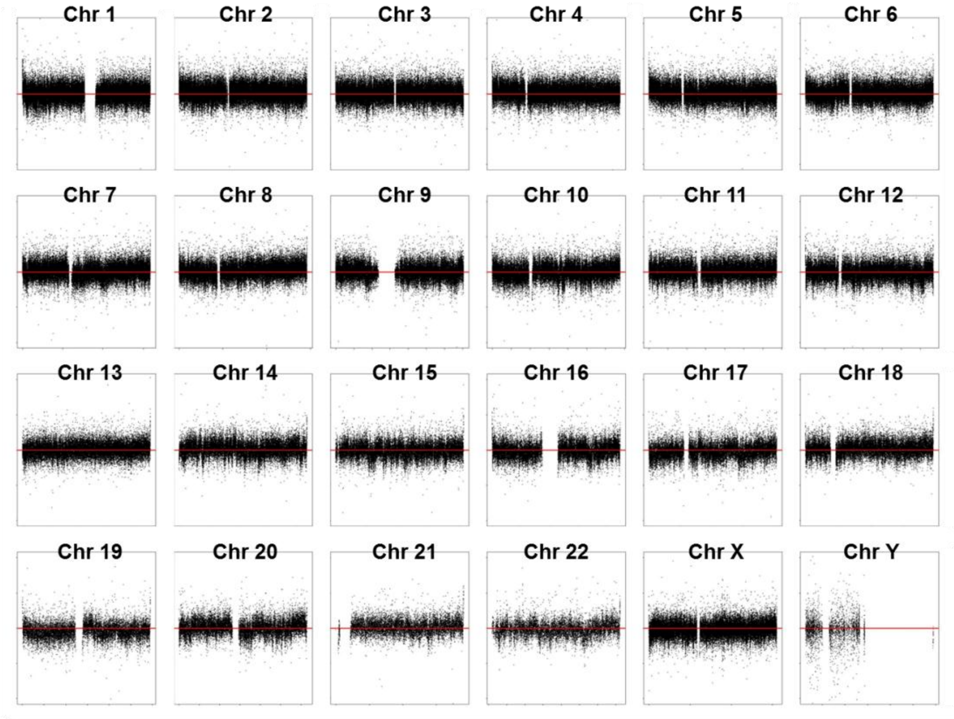

Patient 5

e

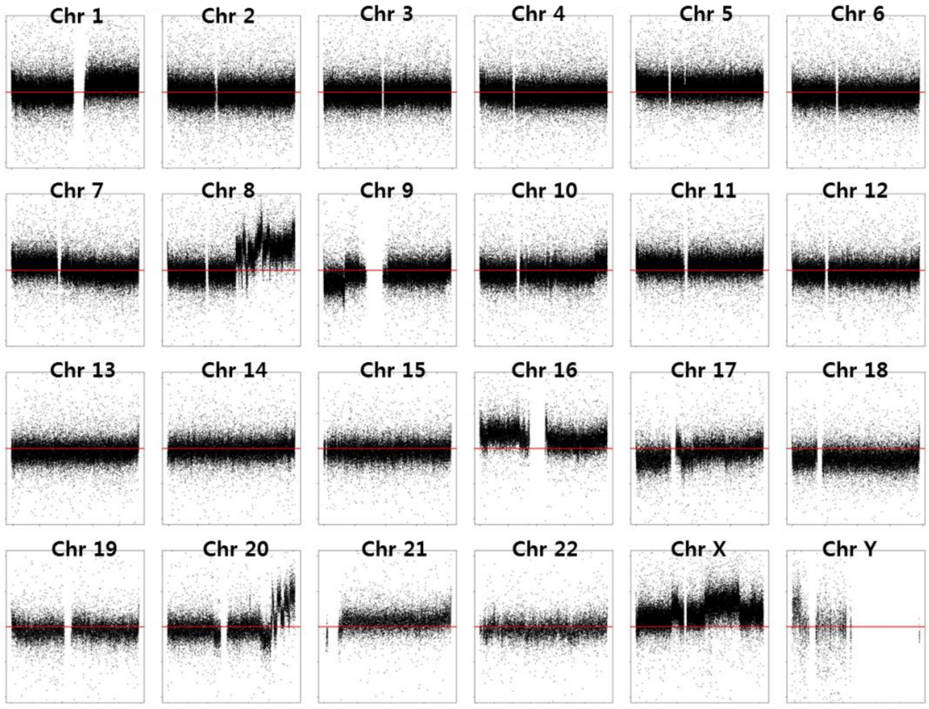

Patient 6

f

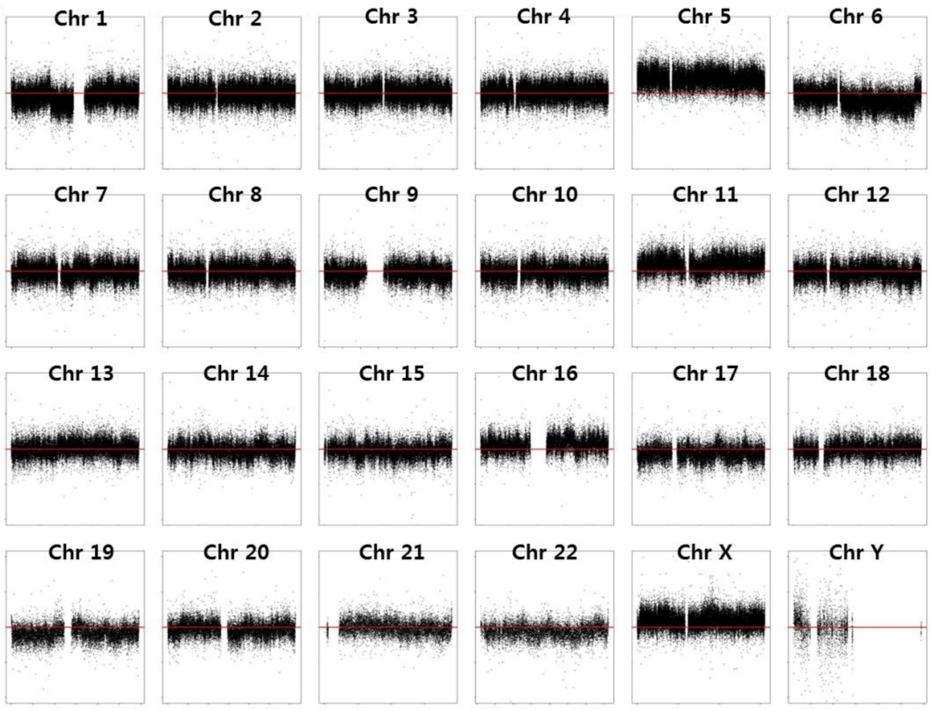

Patient 8

Figure S8. Genomic regions with copy number gains or losses.
Red and green indicate copy number gains or losses, respectively. Results from all six patients were overlaid to show the overall trend. Refer to Supplemental Figure 7 to identify responsible patients for each region.

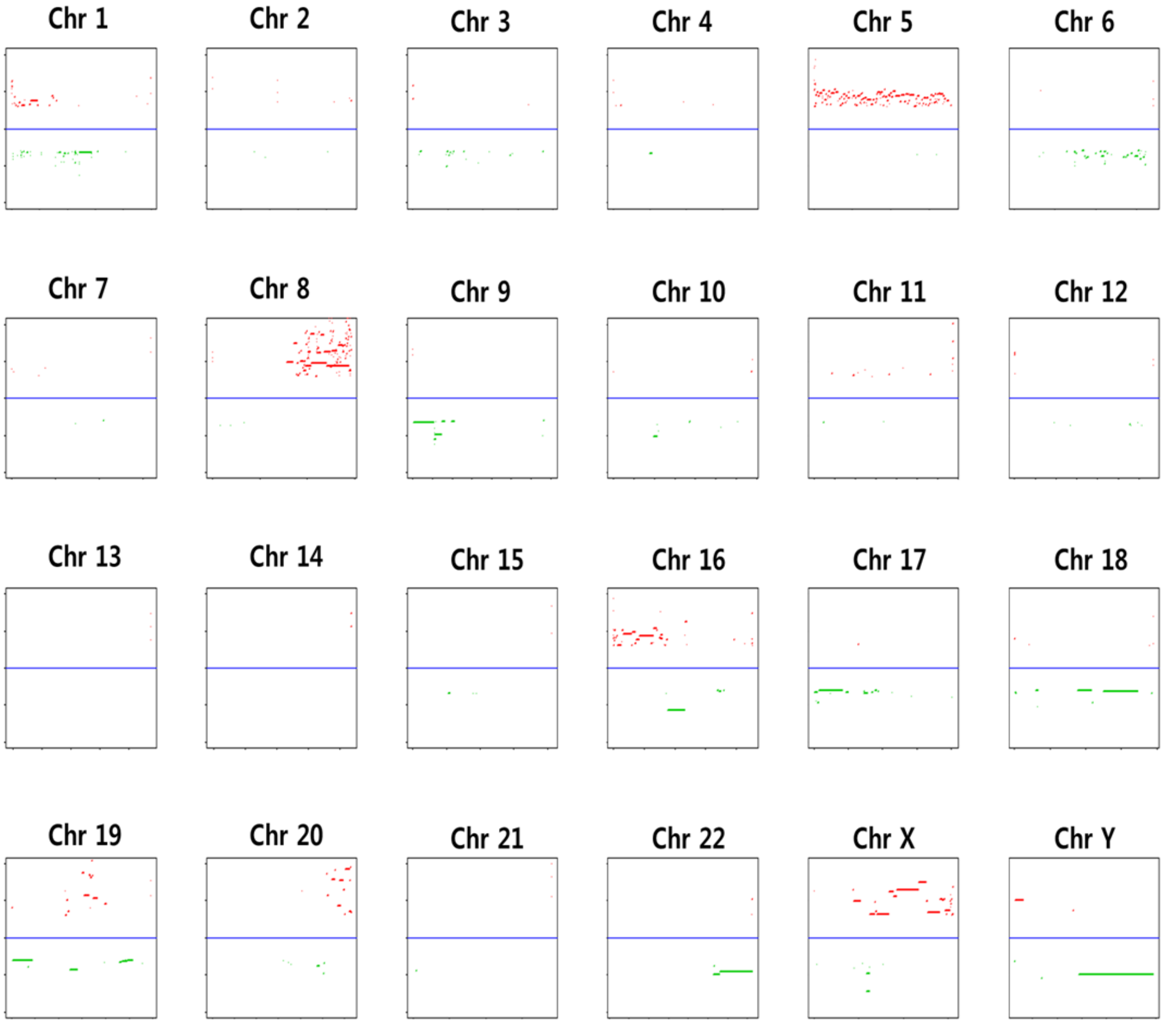

Figure S9. Statistical enrichment test of functional terms in IPA.

P-value after Benjamini-Hochberg multiple test correction was plotted in $-\log_{10}$ scale. Threshold for random permutation is shown in pale grey line.

a. Diseases and Disorders

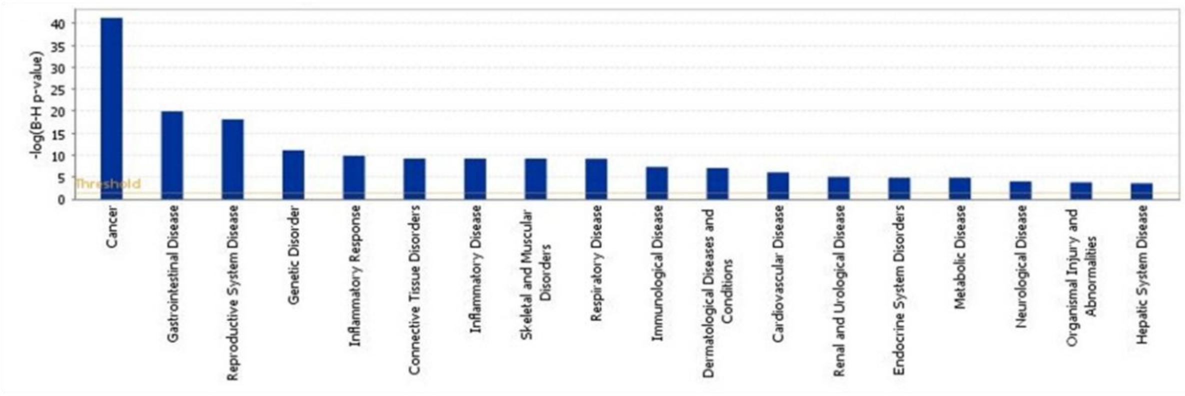

b. Molecular and Cellular Functions

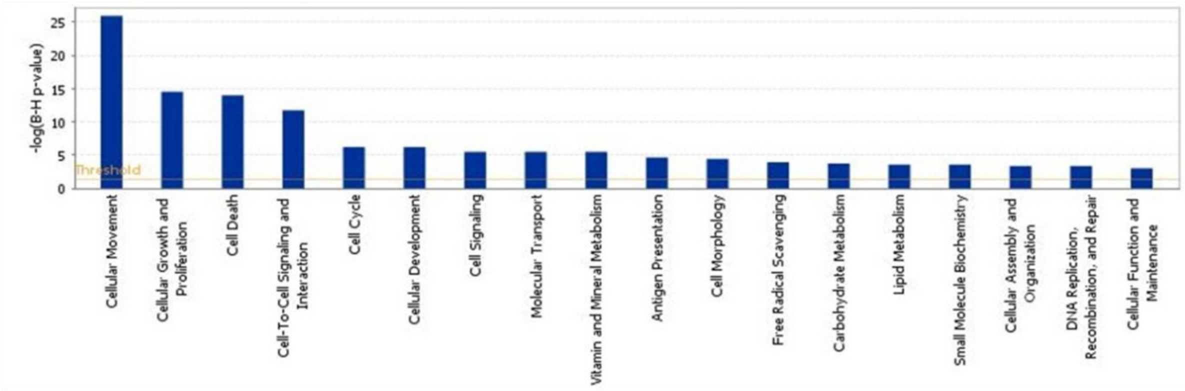

c. Physiological System Development and Function

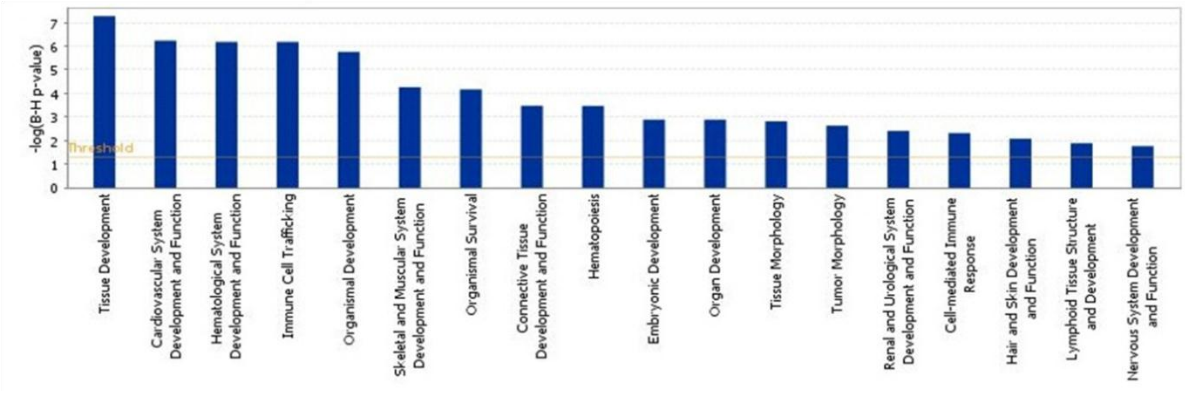

Figure S10. Overall PPI network of 1536 genes.

Gene expression level was overlaid in node color (red for up-regulation and blue for down-regulation in cancer). Circles, rectangles, crosses indicate the DEGs, somatic mutations, fusion genes, respectively.

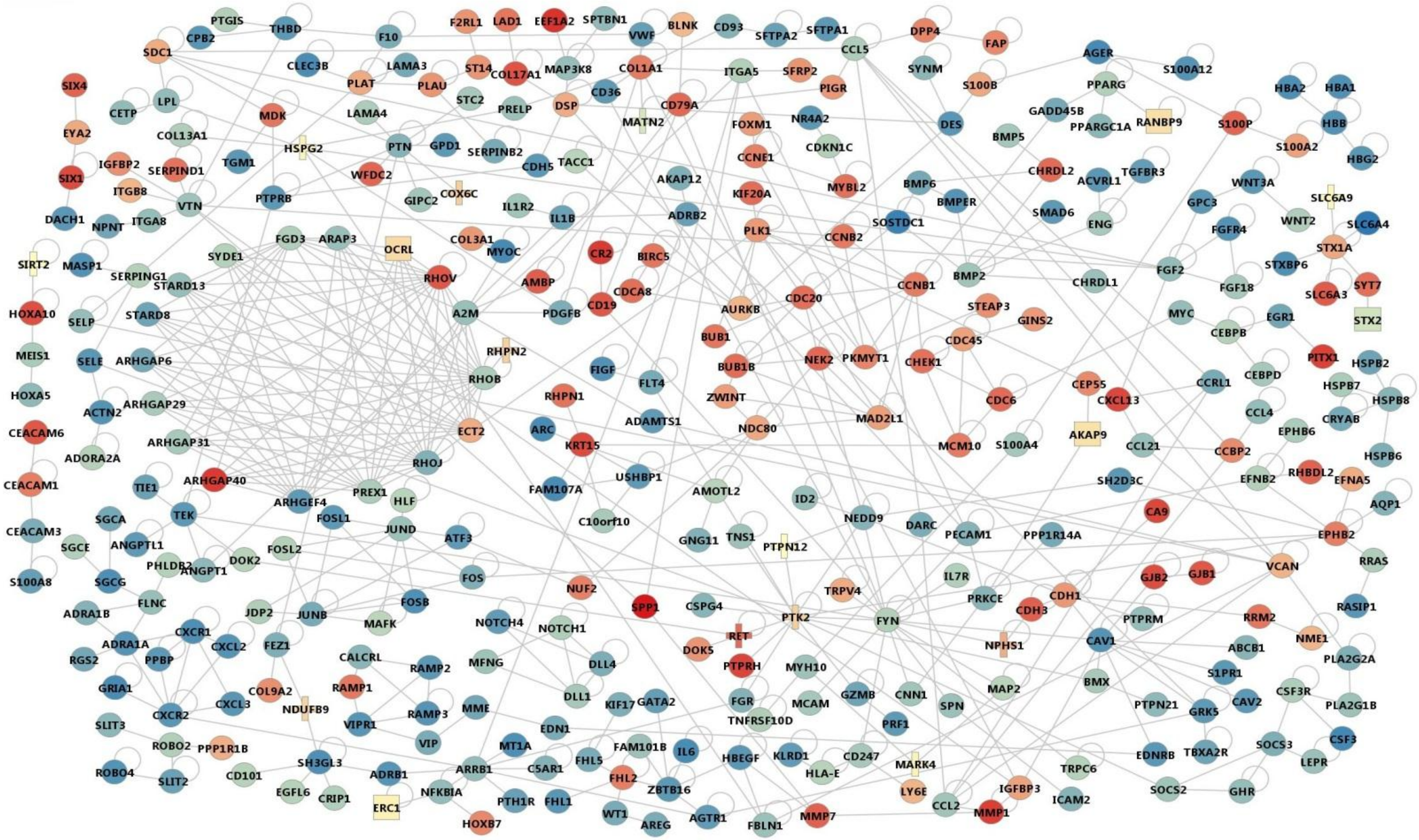

Figure S11. Network modules from MCODE.

Circles, rectangles and crosses represent DEGs, somatic mutations and fusion events, respectively. Node color indicates the expression changes (red for up-regulation and blue for down-regulation in cancer).

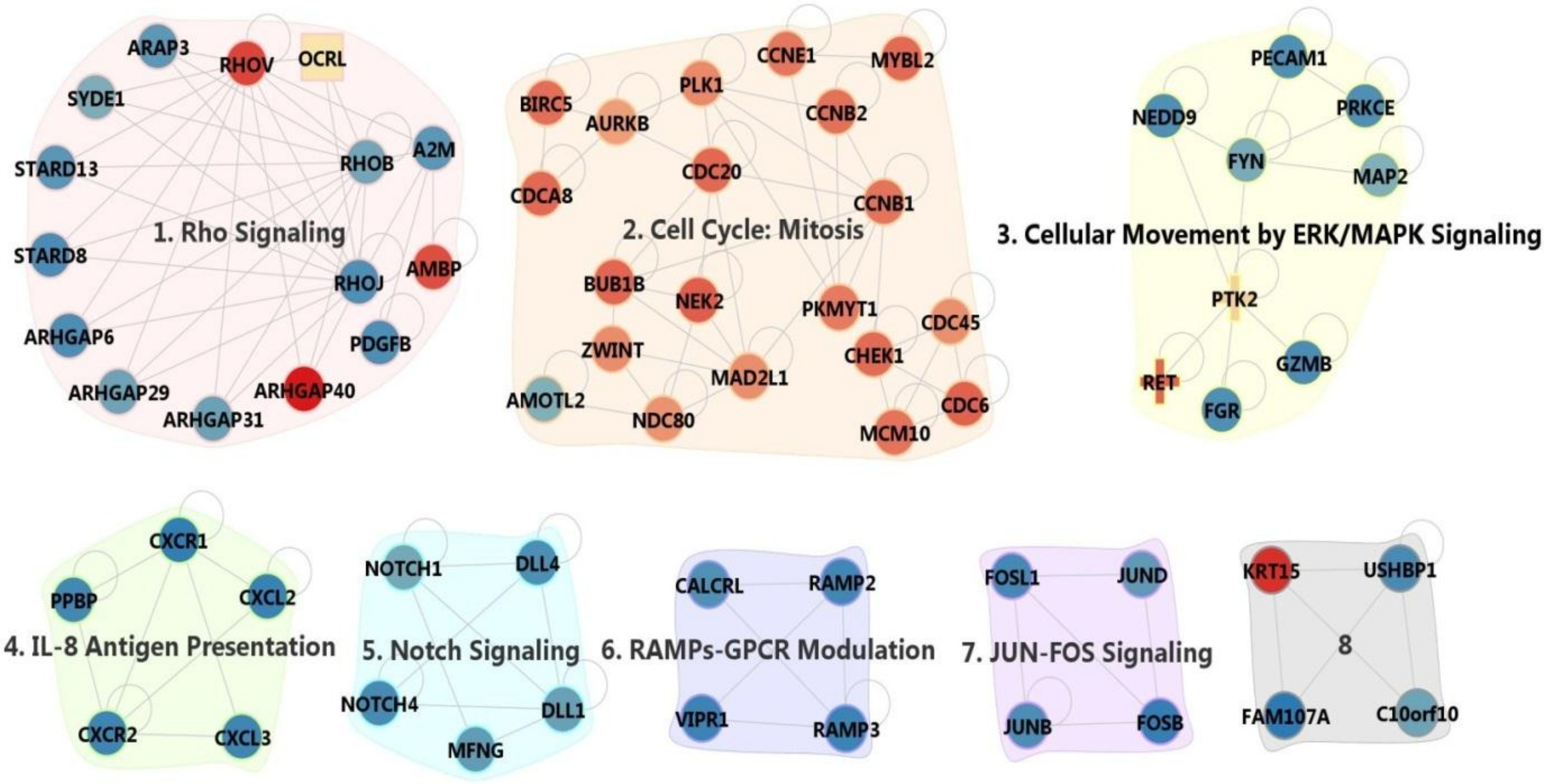

Figure S12. Functional grouping of MCODE modules in 3 broad categories.
Rho signaling is mainly responsible for 'cellular assembly and organization'. Down-regulated modules in Supplemental Figure 11 can be summarized into 'cellular growth and proliferation'.

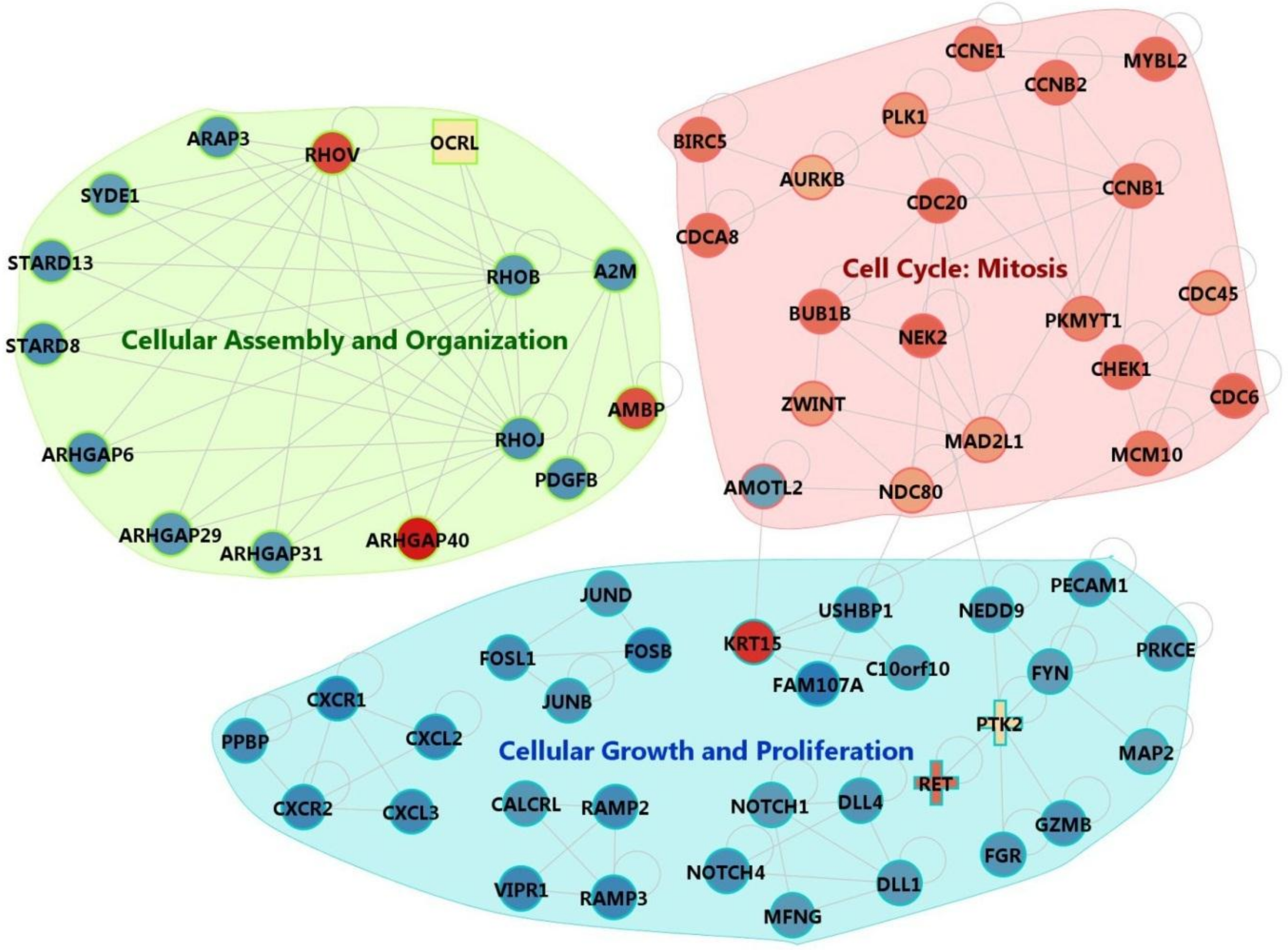

Supplemental Tables

Table S1. Characteristics of Patients

Bank code	Gender	age	Smoking	Family history of lung cancer	cStage*	Location of tumor	Preop. Chx/ Rx***	TNM pStage**	Tumor size (cm)	Cell type	differentiation
PAT1	F	44	No	No	T2aN0M0	Left lower lobe	No	T2aN0M0, 1B	4x4	ADC	Moderately differentiated
PAT3	F	53	No	No	T2aN0M0	Left lower lobe	No	T2aN0M0, 1B	3.2x3	ADC	Moderately differentiated
PAT4	F	60	No	No	T2bN0M0	Right lower lobe	No	T2bN2M0,3A	5.5x5x5	ADC	Moderately differentiated
PAT5	F	44	No	No	T2bN0M0	Left lower lobe	No	T2bN1M0, 2B	5.5x4x3.5	ADC	Poorly differentiated
PAT6	F	70	No	No	T1aN0M0	Right upper lobe	No	T1aN0M0, 1A	1.5x1	ADC	Well differentiated
PAT8	F	62	No	No	T1aN0M0	Left lower lobe	No	T1aN0M0, 1A	2.5x2.5x2	ADC	Moderately differentiated

* cStage; preoperative clinical stage
** pStage: postoperative pathological stage
*** preop Chx/Rx; preoperative chemotherapy/radiotherapy
Note: Patient 3 was also diagnosed with breast cancer unrelated with the NSCLC.

Table S2. Summary statistics for mapping exome-seq data

Sample	Total Reads	Mapped Reads	Reads on Target	Mean Depth	10X Coverage
P1N	39,800,845	38,988,924 (98.0%)	21,912,765 (56.2%)	36.37	78.1%
P1T	32,692,466	32,064,406 (98.1%)	19,303,030 (60.2%)	32.25	78.5%
P3N	31,438,776	30,818,210 (98.0%)	18,812,691 (61.0%)	31.44	79.0%
P3T	35,561,618	34,867,098 (98.1%)	21,371,742 (61.3%)	35.66	79.7%
P4N	35,409,407	34,658,179 (97.9%)	20,182,604 (58.2%)	33.56	79.6%
P4T	35,762,209	34,642,307 (96.9%)	20,532,966 (59.3%)	34.20	78.6%
P5N	32,537,610	31,577,241 (97.1%)	18,851,082 (59.7%)	31.43	77.4%
P5T	37,418,478	36,544,274 (97.7%)	22,190,233 (60.7%)	37.06	79.3%
P6N	34,806,841	34,114,765 (98.0%)	20,093,569 (58.9%)	33.54	78.1%
P6T	36,664,516	35,898,987 (97.9%)	21,080,018 (58.7%)	35.21	78.9%
P8N	34,234,550	33,406,156 (97.6%)	19,588,329 (58.6%)	32.68	77.5%
P8T	32,282,577	31,553,001 (97.7%)	18,105,065 (57.4%)	30.26	76.8%

N: normal, T: tumor.

Table S3. List of experimentally confirmed somatic mutations

Gene	Position	Base Substitution	Amino Acid Change	Sample	VarScan p-value	JSM Somatic Probability	Functional Region	Mutation Type
BAZ1B	chr7:72,877,265	T > C	E>G	P4	.	1.00000	CDS	Missense
GLP2R	chr17:9,765,384	G > A	G>R	P8	.	1.00000	CDS	Missense
GLP2R	chr17:9,765,385	G > C	G>A	P8	.	1.00000	CDS	Missense
IRAK3	chr12:66,610,996	G > A	E>K	P3	.	1.00000	CDS	Missense
KIR3DL1	chr19:55,331,286	G > C	E>D	P1	0.000005	1.00000	CDS	Missense
OCIAD1	chr4:48,851,982	A > G	Y>C	P1	0.000003	1.00000	CDS	Missense
OCRL	chrX:128,682,547	A > G	E>E	P8	.	1.00000	CDS	Silent
OR6K3	chr1:158,687,066	A > C	L>L	P6	.	1.00000	CDS	Silent
PSMD6	chr3:63,999,146	C > T	A>T	P6	.	1.00000	CDS	Missense
RANBP9	chr6:13,632,685	G > C	H>D	P4	.	1.00000	CDS	Missense
ZNF729	chr19:22,486,610	G > A	W>*	P6	.	1.00000	CDS	Nonsense
EML1	chr14:100,367,273	C > A	P>H	P6	0.001913	1.00000	CDS	Missense
POLN	chr4:2,209,719	C > T	D>N	P6	.	1.00000	CDS	Missense
ZNF536	chr19:31,048,126	C > T		P6	.	1.00000	UTR	.
C11orf82	chr11:82,645,226	C > T	P>L	P6	.	1.00000	CDS	Missense
UBASH3A	chr21:43,846,887	T > C	L>L	P1	0.001372	1.00000	CDS	Silent
HPS5	chr11:18,327,815	C > T	G>S	P4	.	1.00000	CDS	Missense
C6orf118	chr6:165,703,451	A > G	L>P	P3	.	1.00000	CDS	Missense
PEX1	chr7:92,132,501	C > T	D>N	P3	.	1.00000	CDS	Missense
THOC2	chrX:122,778,498	T > A	E>V	P1	0.000284	1.00000	CDS	Missense
FBXO11	chr2:48,049,365	T > C	D>A	P6	0.004079	1.00000	CDS	Missense
AKAP9	chr7:91,624,022	G > A	D>N	P6	0.001492	1.00000	CDS	Missense
HTR1E	chr6:87,725,857	G > A	D>N	P4	.	1.00000	CDS	Missense
TUBB1	chr20:57,599,185	G > A	G>S	P6	.	1.00000	CDS	Missense
MAGEE1	chrX:75,650,266	C > A	P>Q	P1	0.002037	1.00000	CDS	Missense
LAMB1	chr7:107,570,034	G > A	A>V	P6	0.002639	1.00000	CDS	Missense
ST3GAL3	chr1:44,363,949	G > A	R>H	P3	.	0.99999	CDS	Missense
COPS3	chr17:17,174,264	G > A	Q>*	P1	0.004029	0.99999	CDS	Nonsense
DNAH8	chr6:38,805,769	C > A	Q>K	P1	0.000016	0.99999	CDS	Missense
DYRK3	chr1:206,821,735	A > T	I>F	P8	0.001220	0.99998	CDS	Missense
GUSB	chr7:65,432,795	C > G	E>Q	P3	0.015379	0.99996	CDS	Missense
AXDND1	chr1:179,414,225	C > T	R>W	P6	.	0.99996	CDS	Missense
UBR4	chr1:19,513,749	T > A	Q>L	P1	.	0.99996	CDS	Missense

L3MBTL2	chr22:41,621,880	C > T	S>F	P6	0.005690	0.99987	CDS	Missense
RHAG	chr6:49,578,748	G > A	G>G	P6	0.004376	0.99987	CDS	Silent
SH3PXD2A	chr10:105,377,004	G > A	G>G	P3	.	0.99987	CDS	Silent
HLTF	chr3:148,789,413	T > C	D>G	P1	0.037203	0.99986	CDS	Missense
HUNK	chr21:33,368,156	C > T	L>L	P8	.	0.99984	CDS	Silent
ACCN1	chr17:32,483,181	G > A	P>L	P6	0.004968	0.99977	CDS	Missense
STX2	chr12:131,293,219	C > A	S>I	P6	0.042462	0.99977	CDS	Missense
PRG4	chr1:186,280,672	C > T	A>V	P4	0.001341	0.99965	CDS	Missense
FRMD5	chr15:44,180,429	A > T	T>T	P6	0.016518	0.99959	CDS	Silent
ERC1	chr12:1,517,325	G > T	R>L	P6	.	0.99927	CDS	Missense
COL19A1	chr6:70,733,529	G > A	G>E	P6	0.058559	0.99925	CDS	Missense
P2RY1	chr3:152,554,413	C > T	T>M	P4	.	0.99908	CDS	Missense
CELF4	chr18:35,145,519	C > T	S>N	P3	0.010098	0.99651	CDS	Missense
MAGEA2	chrX:151,885,759	C>A	A>D	P8	0.000021	N/A	CDS	Missense

* Entries with the missing *VarScan* p-value were not predicted as somatic mutations in *VarScan*.

Table S4. RNA-Seq data mapping summary

Sample	Total Reads	Mapped Reads	Unique Read 1	Unique Read 2	Mean Depth
P1N	77,029,708	28,883,762 (37.5%)	8,748,649	14,318,738	40.1
P1T	68,027,769	23,091,622 (33.9%)	8,177,784	13,437,274	32.0
P3N	68,729,173	27,020,525 (39.3%)	9,396,628	15,236,972	37.5
P3T	82,594,990	20,698,728 (25.1%)	7,942,937	13,255,831	28.7
P4N	71,638,908	32,253,913 (45.0%)	7,727,492	12,780,807	44.8
P4T	68,361,098	22,305,290 (32.6%)	6,791,026	11,454,601	31.0
P5N	68,476,906	24,964,793 (36.5%)	6,992,409	11,972,068	34.6
P5T	58,368,764	24,472,353 (41.9%)	6,581,958	11,293,555	34.0
P6N	53,972,605	27,149,300 (50.3%)	6,595,260	11,268,793	37.7
P6T	59,002,356	15,562,845 (26.4%)	6,543,922	11,268,793	21.6
P8N	62,685,510	20,648,179 (32.9%)	5,531,378	5,321,359	28.7
P8T	63,815,679	15,389,891 (24.1%)	5,260,089	5,027,813	21.4

* N, normal; T, tumor.

Table S5. Number of differentially regulated genes and isoforms

**Number of differentially regulated genes**													
nPatDE	nPat2fold (UP)					nPat2fold (DOWN)							Sum
	6	5	4	3	2	6	5	4	3	2	1	0	
6	98	89	36	6		289	180	104	36	4			842
5	6	89	31	4		3	103	61	12	1			310
4		5	85	7	1	1	4	44	19	1	2	1	170
3		3	2	81		1	2	3	40	4	1		137
Sum	104	186	154	98	1	294	289	212	107	10	3	1	1459
**Number of differentially regulated isoforms**													
nPatDE	nPat2fold (UP)					nPat2fold (DOWN)							Sum
	6	5	4	3	2	6	5	4	3	2	1	0	
6	81	50	18	2		260	133	52	7	1			604
5	9	90	21			18	132	49	3				322
4	3	17	73	1		10	20	64	14	1			203
3	3	6	9	76	1	1	18	21	53	2	1		191
Sum	96	163	121	79	1	289	303	186	77	4	1		1320

* nPatDE = number of patients with differential expression in *edgeR* test (FDR cutoff = 0.01) for each patient
* nPat2fold = number of patients over two fold expression change

Table S6. Primer sequences for detecting fusion transcripts

Fusion gene	Primer sequence (5'-3')-F	Primer sequence (5'-3')-R
RHPN2-PEPD	GGCCGGAGTAAATTGCAGAA	CGCAGATGCAGGTGTAGGAG
SIRT2-NPHS1	GGCTGGAACAGGAGGACTTG	AGCTCTGAGTGTCCCGCTCT
COX6C-LAPTM4B	AACTACCATGGCTCCCGAAGT	TGCCGTATGTATTCCTGAATGG
MARK4-ERCC2	GACTACCTCGTGTCGCATGG	AGTCGATTCCCTCGGACACTT
NDUFB9-PGCP	CAAGGTCCCAGAATGGTGCT	AGGCAGCATTTCTTCCATGTC
SLC6A9-MORN1	TGCTCTTGAGATCTGTGGCC	AGGCTGTCCAGGAACAGGAA
STK3-PTK2	TCGGATGAAGATGAGCTGGA	AATACTGGCCCAGGTGGTTG
PTK2-FAM84B	GGCAGCTGCTTACCTTGACC	AGCTGAAGTTGTGGGCTCCT
PKHD1L1-MATN2	CGTGGGATCAGGATTCAGTG	AGGCAGTGGCAGGAATAGGA
MKL1-NIPA1	CATCTTTCCCACCCTCTTGGT	AGGAAGCTAAAATGGACCCGA
HSPG2-TMCO4	CCTACACACGCCACCTGATCT	TCGGGTTCAGGAAATAACTGG
NIPAL3-ATAD3B	TGATTGCCAGTGTGGGCTAC	TCTGTGGCCGGCTTAAGAAC
UBFD1-CDH11	CAGGCAGAAACAACACAGGAA	CCGCAGACTTTGATGGTGAG
SLC7A6-LRRC36	GCCTGTGGGTCTCACTGCTA	GCCGGGACTACCAAAGACAG
KDM6A-WSB1	GTTGATCCCAGCTTTTGTCG	ACTGTGCGATGTCCTTGTGAC
EIF1AX-PDE4DIP	CTGGCCTCCAGCACCTACTT	GCTGGTGAGCCTCTCTGTCA
GRHL2-PTPN12	GCCCATGGAAGAGGAGTTTG	GTCCTTGGTGGTTTTGGAGG
CCDC6-RET	CCTGGAGGAGCTCACCAACC	TACCCTGCTCTGCCTTTCAGA
GLE1-CCBL1	TCAGCCCTAGATCAACCCTCA	GCATGAAGTCTCCACTGACAGC

Table S7. Mapping statistics of small RNA-Seq data

Sample	Total reads	3' removed reads	Reads on miRNA	Unmapped	Unique reads
P1N	28,645,348	17,741,526	12,998,220 (73.3%)	4,743,306 (26.7%)	468,113
P1T	34,742,127	22,193,690	17,356,451 (78.2%)	4,837,239 (21.8%)	384,296
P3N	32,728,775	23,684,929	15,457,357 (65.3%)	8,227,572 (34.7%)	390,295
P3T	30,531,469	19,386,535	14,321,414 (73.9%)	5,065,121 (26.1%)	297,653
P4N	29,140,944	20,325,562	14,202,756 (69.9%)	6,122,806 (30.1%)	367,457
P4T	29,433,890	15,214,198	10,971,932 (72.1%)	4,242,266 (27.9%)	366,762
P5N	30,970,140	22,981,541	16,928,225 (73.7%)	6,053,316 (26.3%)	308,108
P5T	30,105,887	19,611,741	15,517,262 (79.1%)	4,094,479 (20.9%)	336,814
P6N	31,808,386	22,587,121	16,063,355 (71.1%)	6,523,766 (28.9%)	324,151
P6T	28,894,660	15,240,466	11,737,779 (77.0%)	3,502,687 (22.9%)	326,881
P8N	32,016,524	19,651,378	13,281,147 (67.6%)	6,370,231 (32.4%)	317,851
P8T	32,667,150	17,044,498	13,050,868 (76.6%)	3,993,630 (23.4%)	361,839

* N, normal; T, tumor.

Table S8. List of differentially expressed microRNAs

miRNA ID	FDR	logFC	nPatDE	nPat2fold	miRNA ID	FDR	logFC	nPatDE	nPat2fold
hsa-miR-9	5.61E-05	2.44	6	5+	hsa-miR-27a*	6.47E-06	-2.60	6	6-
hsa-miR-9*	6.49E-05	2.38	6	6+	hsa-miR-30a*	3.28E-04	-2.19	6	5-
hsa-miR-96	3.11E-06	2.64	6	6+	hsa-miR-30c-2*	1.24E-04	-2.30	5	5-
hsa-miR-127-3p	4.52E-04	2.14	6	6+	hsa-miR-126	9.23E-07	-2.79	6	6-
hsa-miR-134	1.55E-04	2.31	6	5+	hsa-miR-126*	2.79E-04	-2.06	6	6-
hsa-miR-135b	4.01E-07	2.90	6	6+	hsa-miR-135a	1.59E-04	-2.48	6	5-
hsa-miR-135b*	1.56E-08	3.32	6	6+	hsa-miR-139-5p	3.35E-04	-2.05	6	6-
hsa-miR-182	1.49E-04	2.29	6	5+	hsa-miR-144	7.82E-07	-2.98	6	6-
hsa-miR-183	8.43E-06	2.67	6	5+	hsa-miR-144*	6.54E-07	-2.99	6	5-
hsa-miR-183*	1.53E-06	2.88	6	6+	hsa-miR-206	2.10E-07	-3.54	5	5-
hsa-miR-205	6.79E-08	3.57	5	5+	hsa-miR-338-5p	7.94E-06	-2.58	6	6-
hsa-miR-210	2.49E-04	2.16	5	5+	hsa-miR-338-3p	1.05E-04	-2.30	6	5-
hsa-miR-301b	3.06E-05	2.49	6	6+	hsa-miR-451	1.50E-06	-2.94	6	6-
hsa-miR-380*	3.19E-05	2.76	5	5+	hsa-miR-486-5p	1.17E-07	-3.26	6	5-
hsa-miR-409-3p	7.23E-06	2.93	5	5+	hsa-miR-486-3p	1.20E-04	-2.46	5	5-
hsa-miR-410	4.52E-04	2.33	6	5+	hsa-miR-584	8.48E-05	-2.37	6	5-
hsa-miR-432	4.52E-04	2.24	5	5+	hsa-miR-4532	8.85E-05	-2.37	6	5-
hsa-miR-493	3.23E-04	2.19	5	5+					
hsa-miR-495	8.48E-05	2.48	5	5+					
hsa-miR-877	2.67E-04	2.22	5	5+					
hsa-miR-889	9.06E-04	2.12	5	5+					
hsa-miR-1246	2.14E-04	2.55	4	5+					
hsa-miR-1269	1.87E-18	9.48	6	3+					

* FDR, log_2FC(fold change) from *edgeR* program
* nPatDE = number of patients with differential expression in *edgeR* test (FDR cutoff = 0.01) for each patient
* nPat2fold = number of patients over two fold expression change (+,- indicates up- or downregulation in tumor tissue)

Table S9. Inversely correlated microRNA-target relations

microRNA*	DEG Name	miRNA exp log_2FC	DEG exp log_2FC	Pearson Correlation	P-value for Correlation	Validation**
hsa-miR-126	*SOX2*	-2.790	1.739	-0.594	0.041	O
hsa-miR-126*	*EPHB2*	-2.060	1.891	-0.657	0.020	O
hsa-miR-139-5p	*ARHGEF38*	-2.048	1.718	-0.707	0.010	X
hsa-miR-139-5p	*ENC1*	-2.048	1.778	-0.628	0.029	X
hsa-miR-139-5p	*GALNT7*	-2.048	2.070	-0.707	0.010	X
hsa-miR-139-5p	*MEX3A*	-2.048	2.265	-0.635	0.027	X
hsa-miR-139-5p	*POF1B*	-2.048	2.785	-0.656	0.021	X
hsa-miR-139-5p	*TMEM184A*	-2.048	1.861	-0.754	0.005	X
hsa-miR-27a*	*PKMYT1*	-2.595	1.818	-0.638	0.026	O
hsa-miR-30a*	*MYBL2*	-2.189	2.029	-0.625	0.030	O
hsa-miR-30c-2*	*MYBL2*	-2.299	2.029	-0.685	0.014	O
hsa-miR-338-3p	*BCL2L15*	-2.298	2.763	-0.688	0.013	X
hsa-miR-338-3p	*FBXO32*	-2.298	2.267	-0.770	0.003	X
hsa-miR-338-3p	*GPT2*	-2.298	2.293	-0.818	0.001	X
hsa-miR-338-3p	*MACC1*	-2.298	1.696	-0.688	0.013	X
hsa-miR-338-3p	*NHS*	-2.298	2.990	-0.713	0.009	X
hsa-miR-338-3p	*RASAL1*	-2.298	4.046	-0.638	0.026	X
hsa-miR-338-3p	*SOX4*	-2.298	1.470	-0.718	0.009	X
hsa-miR-338-5p	*CDCA8*	-2.577	2.072	-0.627	0.029	X
hsa-miR-338-5p	*ERO1L*	-2.577	2.001	-0.727	0.007	X
hsa-miR-338-5p	*ESRP1*	-2.577	1.381	-0.659	0.020	X
hsa-miR-338-5p	*HMGB3*	-2.577	2.708	-0.595	0.041	X
hsa-miR-338-5p	*HOXB2*	-2.577	1.554	-0.634	0.027	X
hsa-miR-338-5p	*SIX1*	-2.577	3.695	-0.735	0.006	X
hsa-miR-486-3p	*IGSF9*	-2.454	1.884	-0.658	0.020	X
hsa-miR-486-3p	*PPP1R14B*	-2.454	1.325	-0.697	0.012	X
hsa-miR-486-3p	*SBK1*	-2.454	2.228	-0.650	0.022	X
hsa-let-7a	*EPHB2*	-0.957	1.891	-0.915	0.000	O
hsa-let-7a	*HOXA10*	-0.957	3.500	-0.595	0.041	O
hsa-let-7a	*NCAPG*	-0.957	1.859	-0.680	0.015	O
hsa-let-7a	*RET*	-0.957	2.220	-0.578	0.049	O
hsa-let-7b	*AURKB*	-1.138	1.375	-0.636	0.026	O
hsa-let-7b	*EPHB2*	-1.138	1.891	-0.793	0.002	O
hsa-let-7b	*GALNT7*	-1.138	2.070	-0.712	0.009	O
hsa-let-7b	*IQGAP3*	-1.138	2.118	-0.627	0.029	O
hsa-let-7b	*KCNN4*	-1.138	2.746	-0.595	0.041	O
hsa-let-7b	*MSI2*	-1.138	1.475	-0.691	0.013	O
hsa-let-7b	*NCAPG*	-1.138	1.859	-0.579	0.049	O
hsa-let-7b	*NT5E*	-1.138	1.937	-0.577	0.049	O
hsa-let-7b	*PSAT1*	-1.138	2.233	-0.711	0.010	O
hsa-let-7b	*RET*	-1.138	2.220	-0.542	0.069	O

hsa-let-7b	*TMED3*	-1.138	1.359	-0.628	0.029	O
hsa-let-7b	*TRIP13*	-1.138	1.789	-0.582	0.047	O
hsa-let-7b	*UHRF1*	-1.138	1.883	-0.626	0.029	O
hsa-miR-1	*CENPF*	-1.669	2.106	-0.631	0.028	O
hsa-miR-1	*RHOV*	-1.669	3.142	-0.695	0.012	O
hsa-miR-1	*TRIM2*	-1.669	1.891	-0.577	0.049	O
hsa-miR-1	*UHRF1*	-1.669	1.883	-0.725	0.008	O
hsa-miR-138	*VCAN*	-1.746	1.407	-0.700	0.011	O
hsa-miR-148a*	*BAALC*	1.428	-2.662	-0.637	0.026	O
hsa-miR-148a*	*EPAS1*	1.428	-2.882	-0.709	0.010	O
hsa-miR-148a*	*ERG*	1.428	-1.885	-0.749	0.005	O
hsa-miR-148a*	*HLA-E*	1.428	-1.350	-0.685	0.014	O
hsa-miR-148a*	*MYC*	1.428	-1.782	-0.611	0.035	O
hsa-miR-203	*BIRC5*	-1.610	1.961	-0.642	0.024	O
hsa-miR-21	*ADRB2*	1.730	-2.378	-0.824	0.001	O
hsa-miR-21	*CDKN1C*	1.730	-1.396	-0.691	0.013	O
hsa-miR-21	*CXCL3*	1.730	-3.048	-0.680	0.015	O
hsa-miR-21	*EGFL7*	1.730	-2.410	-0.660	0.020	O
hsa-miR-21	*EGR1*	1.730	-2.240	-0.756	0.004	O
hsa-miR-21	*FOS*	1.730	-2.071	-0.613	0.034	O
hsa-miR-21	*HSPB6*	1.730	-2.074	-0.746	0.005	O
hsa-miR-21	*PRKCE*	1.730	-1.947	-0.775	0.003	O
hsa-miR-21	*RECK*	1.730	-1.571	-0.770	0.003	O
hsa-miR-21	*RHOB*	1.730	-1.474	-0.720	0.008	O
hsa-miR-21	*SEMA6A*	1.730	-2.921	-0.802	0.002	O
hsa-miR-21	*SOCS3*	1.730	-2.082	-0.671	0.017	O
hsa-miR-21	*TCF21*	1.730	-2.936	-0.681	0.015	O
hsa-miR-21	*TGFBR3*	1.730	-2.416	-0.821	0.001	O
hsa-miR-21	*TIMP3*	1.730	-1.602	-0.730	0.007	O
hsa-miR-21	*WFS1*	1.730	-1.437	-0.680	0.015	O
hsa-miR-218	*BIRC5*	-1.556	1.961	-0.584	0.046	O
hsa-miR-224	*FOSB*	1.689	-3.776	-0.685	0.014	O
hsa-miR-224	*JUNB*	1.689	-2.162	-0.660	0.019	O
hsa-miR-30a	*SLC6A3*	-1.742	3.060	-0.589	0.044	O
hsa-miR-30b*	*MYBL2*	-1.092	2.029	-0.784	0.003	O
hsa-miR-34a	*CRIM1*	1.126	-1.384	-0.618	0.032	O
hsa-miR-375	*BDNF*	1.385	-3.438	-0.601	0.039	O
hsa-miR-375	*FABP4*	1.385	-3.066	-0.580	0.048	O
hsa-miR-375	*PPARG*	1.385	-1.399	-0.632	0.028	O
hsa-miR-135b	*ARC*	2.905	-4.397	-0.700	0.011	X
hsa-miR-135b	*ARHGAP6*	2.905	-2.177	-0.764	0.004	X
hsa-miR-135b	*ARHGEF4*	2.905	-2.477	-0.686	0.014	X
hsa-miR-135b	*BMPER*	2.905	-3.268	-0.663	0.019	X
hsa-miR-135b	*EFNB2*	2.905	-1.379	-0.735	0.007	X

hsa-miR-135b	*FERMT2*	2.905	-1.375	-0.788	0.002	X
hsa-miR-135b	*GHR*	2.905	-1.788	-0.712	0.009	X
hsa-miR-135b	*GRK5*	2.905	-2.511	-0.782	0.003	X
hsa-miR-135b	*KIAA1324L*	2.905	-1.605	-0.637	0.026	X
hsa-miR-135b	*KLF13*	2.905	-1.384	-0.721	0.008	X
hsa-miR-135b	*KLF4*	2.905	-3.234	-0.759	0.004	O
hsa-miR-135b	*LATS2*	2.905	-1.296	-0.762	0.004	X
hsa-miR-135b	*LDB2*	2.905	-2.454	-0.759	0.004	X
hsa-miR-135b	*LRCH2*	2.905	-1.368	-0.702	0.011	X
hsa-miR-135b	*MASP1*	2.905	-3.558	-0.696	0.012	X
hsa-miR-135b	*MSRB3*	2.905	-1.365	-0.677	0.016	X
hsa-miR-135b	*NDRG4*	2.905	-3.020	-0.614	0.034	X
hsa-miR-135b	*NTNG1*	2.905	-2.765	-0.750	0.005	X
hsa-miR-135b	*PHLDB2*	2.905	-1.385	-0.769	0.003	X
hsa-miR-135b	*PHOSPHO1*	2.905	-2.645	-0.725	0.008	X
hsa-miR-135b	*PITPNM2*	2.905	-1.304	-0.686	0.014	X
hsa-miR-135b	*RECK*	2.905	-1.571	-0.674	0.016	X
hsa-miR-135b	*SLC6A4*	2.905	-6.677	-0.576	0.050	X
hsa-miR-135b	*SPTBN1*	2.905	-1.841	-0.723	0.008	X
hsa-miR-135b	*TRPC6*	2.905	-1.357	-0.691	0.013	X
hsa-miR-182	*AATK*	2.286	-2.766	-0.583	0.046	X
hsa-miR-182	*ANGPTL1*	2.286	-2.520	-0.687	0.013	X
hsa-miR-182	*APLN*	2.286	-2.899	-0.666	0.018	X
hsa-miR-182	*ARHGAP29*	2.286	-1.512	-0.757	0.004	X
hsa-miR-182	*ATOH8*	2.286	-2.939	-0.633	0.027	X
hsa-miR-182	*BDNF*	2.286	-3.438	-0.676	0.016	X
hsa-miR-182	*C10orf54*	2.286	-1.894	-0.625	0.030	X
hsa-miR-182	*C13orf36*	2.286	-5.180	-0.633	0.027	X
hsa-miR-182	*CACNA2D2*	2.286	-1.805	-0.610	0.035	X
hsa-miR-182	*CBFA2T3*	2.286	-1.740	-0.691	0.013	X
hsa-miR-182	*CLIC5*	2.286	-2.774	-0.737	0.006	X
hsa-miR-182	*DKK2*	2.286	-1.432	-0.720	0.008	X
hsa-miR-182	*DOCK4*	2.286	-1.304	-0.789	0.002	X
hsa-miR-182	*EDNRB*	2.286	-2.962	-0.629	0.028	X
hsa-miR-182	*EFNB2*	2.286	-1.379	-0.754	0.005	X
hsa-miR-182	*EGR3*	2.286	-2.181	-0.720	0.008	X
hsa-miR-182	*ELMO1*	2.286	-1.483	-0.667	0.018	X
hsa-miR-182	*EPAS1*	2.286	-2.882	-0.667	0.018	X
hsa-miR-182	*ERG*	2.286	-1.885	-0.843	0.001	X
hsa-miR-182	*FAM107A*	2.286	-4.328	-0.639	0.025	X
hsa-miR-182	*FAM167A*	2.286	-1.634	-0.583	0.047	X
hsa-miR-182	*FAM43A*	2.286	-1.988	-0.624	0.030	X
hsa-miR-182	*FIGF*	2.286	-3.573	-0.734	0.007	X
hsa-miR-182	*FOSL2*	2.286	-1.381	-0.692	0.013	X

hsa-miR-182	*FOXF2*	2.286	-2.232	-0.641	0.025	X
hsa-miR-182	*GPC3*	2.286	-2.732	-0.649	0.022	X
hsa-miR-182	*GRIA1*	2.286	-4.300	-0.763	0.004	X
hsa-miR-182	*HBEGF*	2.286	-2.880	-0.625	0.030	X
hsa-miR-182	*IGSF10*	2.286	-2.945	-0.681	0.015	X
hsa-miR-182	*KIAA1324L*	2.286	-1.605	-0.655	0.021	X
hsa-miR-182	*KIF26A*	2.286	-2.337	-0.627	0.029	X
hsa-miR-182	*KLF13*	2.286	-1.384	-0.672	0.017	X
hsa-miR-182	*LIMCH1*	2.286	-1.528	-0.602	0.038	X
hsa-miR-182	*LPHN2*	2.286	-2.529	-0.763	0.004	X
hsa-miR-182	*LRCH2*	2.286	-1.368	-0.879	0.000	X
hsa-miR-182	*MYADM*	2.286	-2.089	-0.725	0.008	X
hsa-miR-182	*NFASC*	2.286	-1.720	-0.601	0.039	X
hsa-miR-182	*NR4A3*	2.286	-3.354	-0.655	0.021	X
hsa-miR-182	*NRN1*	2.286	-1.921	-0.645	0.024	X
hsa-miR-182	*PDK4*	2.286	-2.396	-0.663	0.019	X
hsa-miR-182	*PDZD2*	2.286	-1.931	-0.652	0.022	X
hsa-miR-182	*PDZD4*	2.286	-1.776	-0.710	0.010	X
hsa-miR-182	*PID1*	2.286	-1.743	-0.631	0.028	X
hsa-miR-182	*PPM1F*	2.286	-1.772	-0.717	0.009	X
hsa-miR-182	*PRKCE*	2.286	-1.947	-0.620	0.031	X
hsa-miR-182	*RECK*	2.286	-1.571	-0.783	0.003	X
hsa-miR-182	*RHOJ*	2.286	-2.033	-0.746	0.005	X
hsa-miR-182	*SEMA5A*	2.286	-1.991	-0.755	0.005	X
hsa-miR-182	*SH3BP5*	2.286	-1.655	-0.812	0.001	X
hsa-miR-182	*SIK1*	2.286	-2.382	-0.699	0.011	X
hsa-miR-182	*ST6GALNAC3*	2.286	-1.902	-0.700	0.011	X
hsa-miR-182	*STARD13*	2.286	-1.757	-0.804	0.002	X
hsa-miR-182	*TACC1*	2.286	-1.302	-0.700	0.011	X
hsa-miR-182	*TBXA2R*	2.286	-2.278	-0.684	0.014	X
hsa-miR-182	*TNF*	2.286	-1.524	-0.649	0.022	O
hsa-miR-182	*TNS1*	2.286	-1.681	-0.634	0.027	X
hsa-miR-182	*TOX2*	2.286	-2.405	-0.648	0.023	X
hsa-miR-182	*VGLL3*	2.286	-1.408	-0.675	0.016	X
hsa-miR-182	*WWC2*	2.286	-1.631	-0.722	0.008	X
hsa-miR-182	*ZFP36*	2.286	-2.441	-0.620	0.031	X
hsa-miR-183	*AKAP12*	2.674	-2.076	-0.661	0.019	O
hsa-miR-183	*CYYR1*	2.674	-1.829	-0.650	0.022	X
hsa-miR-183	*EGR1*	2.674	-2.240	-0.588	0.044	O
hsa-miR-183	*KHDRBS2*	2.674	-2.691	-0.697	0.012	X
hsa-miR-183	*KLF4*	2.674	-3.234	-0.685	0.014	O
hsa-miR-183	*MAPK4*	2.674	-2.169	-0.597	0.040	X
hsa-miR-183	*NR4A2*	2.674	-2.820	-0.655	0.021	X
hsa-miR-183	*PKNOX2*	2.674	-2.532	-0.603	0.038	X

hsa-miR-205	*MFNG*	3.566	-1.653	-0.646	0.023	X
hsa-miR-210	*BDNF*	2.158	-3.438	-0.625	0.030	O
hsa-miR-210	*COX4I2*	2.158	-3.098	-0.663	0.019	O
hsa-miR-210	*MDGA1*	2.158	-2.121	-0.592	0.043	O
hsa-miR-210	*PECAM1*	2.158	-1.995	-0.760	0.004	O
hsa-miR-493	*ANKRD29*	2.187	-2.770	-0.677	0.016	X
hsa-miR-493	*SLC2A3*	2.187	-1.876	-0.578	0.049	X
hsa-miR-877	*FAM167A*	2.221	-1.634	-0.608	0.036	X
hsa-miR-9	*CRIM1*	2.441	-1.384	-0.584	0.046	X
hsa-miR-9	*ERG*	2.441	-1.885	-0.608	0.036	X
hsa-miR-9	*MME*	2.441	-2.237	-0.614	0.034	X
hsa-miR-9	*STARD13*	2.441	-1.757	-0.615	0.033	X
hsa-miR-96	*ARHGAP29*	2.641	-1.512	-0.621	0.031	X
hsa-miR-96	*ARHGAP6*	2.641	-2.177	-0.595	0.041	X
hsa-miR-96	*CLIC5*	2.641	-2.774	-0.664	0.019	X
hsa-miR-96	*COL13A1*	2.641	-1.450	-0.599	0.040	X
hsa-miR-96	*CSRNP1*	2.641	-2.793	-0.578	0.049	X
hsa-miR-96	*DOCK4*	2.641	-1.304	-0.634	0.027	X
hsa-miR-96	*ERG*	2.641	-1.885	-0.687	0.014	X
hsa-miR-96	*GPM6A*	2.641	-4.222	-0.614	0.034	X
hsa-miR-96	*GRIA1*	2.641	-4.300	-0.594	0.042	X
hsa-miR-96	*LPHN2*	2.641	-2.529	-0.648	0.023	X
hsa-miR-96	*LRCH2*	2.641	-1.368	-0.683	0.014	X
hsa-miR-96	*NDUFA4L2*	2.641	-1.357	-0.588	0.044	X
hsa-miR-96	*PPM1F*	2.641	-1.772	-0.582	0.047	X
hsa-miR-96	*RAPGEF4*	2.641	-1.872	-0.702	0.011	X
hsa-miR-96	*RECK*	2.641	-1.571	-0.583	0.047	X
hsa-miR-96	*SEMA5A*	2.641	-1.991	-0.656	0.021	X
hsa-miR-96	*SEMA6A*	2.641	-2.921	-0.615	0.033	X
hsa-miR-96	*SH3BP5*	2.641	-1.655	-0.640	0.025	X
hsa-miR-96	*STC2*	2.641	-1.563	-0.670	0.017	X

* Red, blue indicate the up- and downregulated DEmiRs. Non-DEmiRs are shown in black.
** O=validated target in the literature, X=predicted target of DEmiR.

Table S10. Number of genes with copy number variations outside 1.62-2.46 for each patient

	Gain/Loss	P1	P3	P4	P5	P6	P8
Gene	Gain	298	187	184	106	726	390
	Loss	346	151	158	156	304	303
Region	Gain	38	80	46	142	109	81
	Loss	41	87	44	124	49	87

Table S11. Chromosomal gains and losses in previously published papers and in present study

	Kim *et al.* 2005	Garnis *et al.* 2006	Choi *et al.* 2006	Weir *et al.* 2007	Job *et al.* 2010	Present Study
	Array-CGH	Array-CGH	Array-CGH	SNP array	Array-CGH	Array-CGH
	n=50	n= 28 (28 cell lines)	n=15	n=371	n=60	n=6
	NSCLC including 21 adenocarcinomas	NSCLC including 18 adenocarcinomas	Lung adeno-carcinoma	Adeno-carcinomas	Adeno-carcinomas	NSCLC
	Unknown smoking status	2 never smokers	Unknown smoking status	The number of never smokers was not reported in the 371 cases[7]	60 never smokers	6 never smokers
Gains	1q, 3q, 5p, 17p, 19p, 19q, 20q, 22q	1q, 2p, 5p, 7p, 8q, 20q	1p36.33, 2q35, 5q35.3, 7p15.2, 7q35, 8q24.3, 11p15.4, 16p13.3, 17q25.3, 19q13.42, 20q13.33, 21q22.3, 22q13.33	1q, 5p, 6p, 7p, 7q, 8q, 16p, 17q, 20p, 20q	1q21.1, 1q21.2, 1q21.3, 1q32.1, 2p21, 5p15.33, 5p15.32, 5p12-5p11, 5q11.2, 5q35.2-5q35.3, 5q35.3, 7p11.2, 7q11.2, 8q24.21, 8q24.23, 12p11.21, 12q15, 14q13.2-14q21.1, 18p11.32, 20q13.2, 20q13.31, 20q13.33	5p, 10q26, 8q22, 8q23, 8q24, 16p13, 20q13
Losses	3p, 4q, 9p, 17p, Yp, Yq	3p, 4q, 8p, 9p, 10p, 13q, 18q	1p21.1, 1q31.2, 2p16.3–2p16.2, 4q35.1, 5q13.1, 7p12.3, 9p11.2, 11p15.1, 11q12.2, 13q33.1, 14q32.33, 19p13.	3p, 5q, 6q, 8p, 9p, 9q, 10q, 12p, 13q, 15q, 17p, 18q, 19p, 19q, 21q, 22q	1p22.1, 3q11.2, 6q11.1, 8q11.22, 8q11.23, 9p21.3, 9q21.13, 16q23.1, 20p12.1	1p12, 1p13, 9p21

Table S12. Correlations between copy number variations and RNA expression

Gene ID	RNA exp.<br>Mean of log2(FC)	CNV change<br>Mean of log2(FC)	Pearson<br>corr. Coefficient
PTP4A3	0.606	0.409	0.984
RHPN1	1.736	0.315	0.980
NSMCE2	0.793	0.304	0.978
KIAA0196	0.713	0.296	0.977
ZNF572	0.950	0.296	0.962
NIPAL2	0.948	0.271	0.960
NDUFB9	0.746	0.371	0.957
HRSP12	1.204	0.235	0.955
SLC39A4	1.172	0.299	0.951
MTERFD1	0.875	0.254	0.939
PHF20L1	0.774	0.321	0.931
STK3	1.013	0.269	0.930
FAM91A1	1.023	0.277	0.929
CHRAC1	0.608	0.268	0.922
C8orf51	1.074	0.315	0.920
FBXL6	1.018	0.299	0.919
ATAD2	1.332	0.227	0.910
RPL30	0.657	0.235	0.909
POLR2K	0.804	0.261	0.904
ZNF706	0.589	0.230	0.902
C8orf76	1.297	0.262	0.900
GPR172A	1.105	0.299	0.899
RNF139	0.417	0.212	0.898
C8orf47	2.438	0.235	0.894
TOP1MT	0.927	0.329	0.890
WDYHV1	0.549	0.227	0.885
SLC16A1	-0.334	-0.282	0.884
NUDCD1	0.894	0.259	0.880
YWHAZ	0.984	0.231	0.879
JRK	0.448	0.378	0.878
ZHX1	0.405	0.245	0.869
FAM84B	1.249	0.332	0.864
PPP1R16A	0.750	0.299	0.858
SQLE	0.866	0.296	0.855
DERL1	0.825	0.273	0.854
TATDN1	0.984	0.291	0.850
LRIG2	0.031	-0.261	0.842
TSTA3	1.067	0.257	0.840
COX6C	0.866	0.254	0.838
TRMT12	0.557	0.212	0.837
C8orf73	1.972	0.315	0.837

CPSF1	0.772	0.299	0.837
ANKRD46	0.429	0.282	0.836
TMEM65	0.714	0.212	0.835
TSNARE1	0.567	0.382	0.833
EBAG9	0.931	0.338	0.832
TMEM74	0.466	0.351	0.830
ADCK5	1.167	0.299	0.820
ARHGAP39	0.834	0.299	0.810
TRAPPC9	0.671	0.326	0.807
LY6D	3.738	0.378	0.803
PYCRL	0.893	0.257	0.802
FAM83A	4.318	0.262	0.798
PTDSS1	0.243	0.254	0.797
VPS28	0.215	0.299	0.796
ZC3H3	0.460	0.315	0.791
PTK2	0.867	0.286	0.789
KIFC2	0.845	0.299	0.788
FAM49B	0.720	0.210	0.786
RNF19A	0.907	0.245	0.786
PABPC1	1.240	0.279	0.782
ZNF251	0.669	0.278	0.759
FOXH1	0.164	0.299	0.754
ANXA13	0.836	0.288	0.753
ZNF696	0.540	0.329	0.751
ENY2	0.749	0.338	0.748
BAI1	0.195	0.397	0.743
C8orf55	1.001	0.378	0.739
GSDMD	0.241	0.315	0.731
RECQL4	1.204	0.299	0.722
PVT1	1.617	0.342	0.713
WDR67	0.681	0.262	0.706
SLC45A4	0.487	0.400	0.681
LRRC14	0.517	0.299	0.680
POP1	0.522	0.248	0.679
SLURP1	0.425	0.378	0.676
HSF1	0.236	0.226	0.668
BOP1	0.945	0.312	0.660
ZFP41	0.579	0.329	0.656
TSPYL5	1.076	0.309	0.647
LY6E	1.450	0.308	0.634
NDRG1	0.659	0.285	0.630
GLI4	0.162	0.329	0.627
TRIB1	-0.462	0.285	0.625
LOC100131726	0.506	0.262	0.619

LYNX1	-0.056	0.378	0.613
TIGD5	0.495	0.257	0.607
FLJ42969	0.146	0.231	0.606
LRRC6	1.057	0.308	0.596
SNX31	0.183	0.258	0.595
LY6K	1.126	0.378	0.594
LRRC24	-0.222	0.299	0.593
LOC100133669	0.666	0.308	0.591
SPAG1	1.227	0.257	0.587
NAPRT1	0.586	0.257	0.575
ZFAT	0.361	0.297	0.553
PGCP	0.158	0.248	0.552
MTSS1	-0.009	0.321	0.532
GPT	0.235	0.299	0.532
LOC157381	0.367	0.296	0.531
NCRNA00051	0.000	0.367	0.517
LOC728724	0.128	0.331	0.509
PSCA	1.191	0.378	0.509
EEF1D	0.400	0.257	0.506
LY6H	0.058	0.308	0.502
FBXO32	2.201	0.273	0.497
MFSD3	0.964	0.299	0.489
UQCRB	0.310	0.254	0.488
LYPD2	-0.798	0.378	0.477
FBXO43	0.339	0.261	0.455
PCMTD2	0.480	0.318	0.423
SDC2	-0.093	0.254	0.418
WISP1	1.351	0.290	0.395
DENND3	-1.194	0.358	0.393
FER1L6	0.633	0.267	0.386
C8orf31	0.708	0.308	0.357
MYC	-1.599	0.253	0.313
EIF2C2	0.176	0.268	0.282
GDF6	-0.073	0.254	0.263
CDKN2A	0.882	-0.304	0.243
VPS13B	0.961	0.213	0.215
MAFA	0.019	0.315	0.177
KCNK9	0.055	0.434	0.163
ASAP1	0.024	0.229	0.130
ADCY8	-0.819	0.337	0.104
ZHX2	-0.125	0.303	0.096
OSR2	0.439	0.213	0.095
RGS22	-0.111	0.250	0.081
KCNS2	0.159	0.294	0.070

MPG	0.666	0.377	0.069
SYBU	0.521	0.413	0.052
RHBDF1	0.443	0.410	0.035
TG	0.272	0.338	-0.018
GPIHBP1	-2.835	0.308	-0.054
PKHD1L1	-1.464	0.338	-0.072
CYHR1	0.601	0.299	-0.075
SNRNP25	0.896	0.410	-0.085
TMEM71	-0.022	0.321	-0.106
POLR3K	1.060	0.410	-0.143
C8ORFK29	0.028	0.272	-0.152
AKR7A2P1	-0.061	-0.282	-0.243
SLA	-0.751	0.374	-0.251
FLJ43860	-0.010	0.409	-0.396
TRHR	-0.026	0.344	-0.398
C9orf53	0.029	-0.304	-0.451
GML	0.050	0.370	-0.511
KLHL38	0.109	0.288	-0.523
KCNV1	0.027	0.325	-0.523
HPYR1	0.033	0.279	-0.546
GPR20	-0.640	0.409	-0.613
ARC	-2.163	0.378	-0.717
NKX6-2	-0.010	-0.077	-0.737
ST3GAL1	-0.034	0.285	-0.894

* FC = fold change in tumor vs. normal

Table S13. Correlations between DNA methylation and RNA expression

Gene ID	RNA exp.<br>Mean of log2(FC)	Methylation change<br>Mean of log2(T-N)	Pearson<br>corr. Coefficient
BNIPL	0.218	-1.528	-0.971
MT1X	-1.440	1.195	-0.899
DHRS13	0.950	-1.413	-0.833
ADAMTS20	0.046	2.025	-0.764
LOC389333	0.257	1.849	-0.759
FABP5	-0.792	2.038	-0.740
GLRB	0.524	2.598	-0.729
ACBD5	0.266	-1.547	-0.674
FOXC2	-0.260	2.073	-0.662
KLHL35	0.344	1.686	-0.657
KIAA1467	-0.167	-1.163	-0.623
TBX20	0.027	1.997	-0.592
MRPL28	0.403	1.545	-0.579
ATRNL1	0.033	1.814	-0.547
NBPF10	0.195	1.651	-0.539
IL20	-0.248	1.415	-0.531
ITGA6	-0.769	2.286	-0.525
CCL8	-0.520	1.452	-0.521
NEDD8	0.303	1.626	-0.511
PCDHB3	-0.041	1.768	-0.502
HERC5	0.422	-2.029	-0.469
RGS4	-0.663	1.957	-0.454
ZBED2	-2.141	-2.117	-0.441
PERP	1.028	-1.119	-0.439
RBP4	-1.544	1.513	-0.435
POU3F3	0.042	1.768	-0.407
RALYL	0.007	1.575	-0.404
TMEM71	-0.022	1.610	-0.399
NPY2R	0.008	1.862	-0.395
MNT	-0.280	1.887	-0.389
PLEKHM1	-0.256	1.444	-0.387
SYN1	0.334	1.315	-0.375
CLCN2	0.159	-2.061	-0.366
GDNF	0.259	1.306	-0.363
ATM	0.591	1.318	-0.351
ABCB4	0.577	1.523	-0.319
MEP1A	0.193	1.292	-0.304
TMEM109	-0.425	1.582	-0.299
RIPPLY2	-0.020	1.647	-0.281
SLC35A3	0.290	-2.083	-0.279
DUXA	-0.028	1.415	-0.237
FAT1	1.051	1.533	-0.228
GBX2	0.066	2.145	-0.219

FLRT2	-0.418	2.302	-0.185
PCNX	-0.050	1.951	-0.116
DHRS7	0.513	1.771	-0.103
HAND2	0.058	1.601	-0.085
CYB5R1	0.292	1.484	-0.064
TRMT12	0.557	1.431	-0.061
CNTN5	0.025	1.878	-0.053
DDB1	0.396	1.704	-0.051
MEOX2	-0.426	1.796	-0.021
INSM1	-0.014	2.245	-0.005
C5orf38	-0.500	1.885	0.014
OLIG1	-0.053	1.544	0.014
MAML1	0.274	-1.411	0.020
ZNF195	0.681	2.184	0.075
KLHL26	-0.091	-1.425	0.079
LHFPL1	0.091	1.674	0.080
EYA1	0.440	1.528	0.083
CDH9	-0.005	1.195	0.090
ALX1	0.139	1.701	0.092
ZIC3	0.000	1.342	0.108
FAM196B	-0.008	1.920	0.112
HMX1	0.014	2.570	0.121
GRIK2	0.535	1.550	0.131
WWC2	-1.402	1.896	0.141
EPC2	0.064	-1.444	0.145
ETV3	-0.626	1.749	0.146
SLC27A3	-0.715	1.473	0.158
CPT2	0.513	1.918	0.174
ZNF850	0.108	1.710	0.179
AGT	0.730	1.487	0.190
PNLIPRP1	0.000	1.500	0.200
SCN7A	-1.726	-1.814	0.201
CDO1	-2.182	2.050	0.215
FBXL19	0.801	-2.091	0.218
CDH16	0.334	1.573	0.231
DLX6	0.468	1.821	0.233
NKX2-4	0.027	2.196	0.245
SIM1	0.044	2.093	0.247
EFNA4	1.604	1.594	0.268
TAL1	-1.979	1.045	0.274
TTC30A	0.464	1.636	0.280
NEDD8-MDP1	-0.029	1.626	0.287
NDUFS6	0.846	1.846	0.297
LPPR5	-0.260	1.557	0.316
FBXL7	-0.971	-1.224	0.342
ITPR3	0.382	1.751	0.348
HOXB4	-0.108	2.084	0.349

WT1	-0.852	1.824	0.355
TOX3	2.338	1.629	0.362
ZBTB25	0.606	-1.260	0.367
METTL14	0.099	1.749	0.388
FCAMR	0.372	1.415	0.419
FOXB1	0.173	1.852	0.423
HOXB6	0.401	1.516	0.435
DMRTA2	0.612	2.305	0.447
DENND1B	0.692	-1.302	0.454
HMX3	-0.023	1.837	0.459
BTBD7	0.162	2.145	0.485
SPIC	0.037	1.610	0.495
HOXD8	0.644	1.626	0.497
ROCK2	0.014	2.277	0.517
C1orf50	0.505	1.890	0.543
ZNF167	0.279	1.846	0.547
PIK3R2	0.469	-1.814	0.580
SLC10A5	0.418	1.415	0.591
ZNF673	-0.299	1.981	0.606
ATP5J	0.446	1.346	0.617
C8orf82	0.616	1.273	0.638
GPR27	-0.149	1.950	0.643
XCL1	0.252	1.082	0.649
SCAF11	0.344	1.912	0.666
KIAA1409	-0.024	1.751	0.689
ATP6V1A	0.215	-1.452	0.703
YPEL3	-0.146	1.680	0.710
ZNF445	0.066	1.654	0.731
BRIX1	0.543	2.173	0.736
ST3GAL1	-0.034	1.864	0.769
PNMA2	0.865	1.459	0.775
PMM2	0.971	1.890	0.799
DPM1	0.097	1.328	0.814
AJAP1	-0.402	1.723	0.842

* FC = fold change in tumor vs. normal

Table S14. Mapping Summary for MeDIP-Seq data

Sample	Read Length (bp)	# Total Reads	# Mapped Reads	# Unique Reads
P1N	101	41,779,688	39,548,851 (94.66%)	15,793,858 (39.94%)
P1T	101	38,417,109	36,503,317 (95.02%)	15,625,835 (42.81%)
P3N	101	37,574,772	35,756,820 (95.16%)	14,031,413 (39.24%)
P3T	50	50,891,334	42,044,848 (82.62%)	18,107,822 (43.07%)
P4N	50	59,214,920	46,856,827 (79.13%)	15,087,562 (32.20%)
P4T	50	56,687,621	46,337,339 (81.74%)	17,790,651 (38.39%)
P5N	50	58,092,682	46,192,828 (79.52%)	14,840,240 (32.13%)
P5T	50	53,599,728	44,132,418 (82.34%)	16,596,380 (37.61%)
P6N	75	41,654,421	38,132,616 (91.55%)	14,516,599 (38.07%)
P6T	101	39,105,545	36,468,222 (93.26%)	14,089,757 (38.64%)
P8N	50	52,503,587	38,309,352 (72.97%)	12,765,207 (33.32%)
P8T	50	58,414,225	45,588,245 (78.04%)	16,060,922 (35.23%)

*N, normal; T, tumor.

Table S15. Top scoring gene functions from Ingenuity Pathway Analysis (IPA)

ID	Top Functions	Score	Focus Molecules
1	Tissue Morphology, Hematological System Development and Function, Cellular Growth and Proliferation	30	32
2	Cell Cycle, Cancer, Cellular Assembly and Organization	28	31
3	Cell Death, Cancer, Cellular Movement	24	29
4	Cellular Growth and Proliferation, Cell Death, Hematological System Development and Function	23	28
5	Skeletal and Muscular System Development and Function, Tissue Morphology, Cardiovascular Disease	20	26
6	Cancer, Dermatological Diseases and Conditions, Cell Death	15	18
7	Cell-To-Cell Signaling and Interaction, Cellular Movement, Immune Cell Trafficking	13	21
8	Cellular Growth and Proliferation, Cancer, Genetic Disorder	12	20
9	Embryonic Development, Organ Development, Organismal Development	11	19
10	Cancer, Cellular Development, Gene Expression	11	19

* Focus molecules: the number of Network Eligible Molecules per network.

Supplemental References

1. Li H, Durbin R (2009) Fast and accurate short read alignment with Burrows-Wheeler transform. Bioinformatics 25: 1754-1760.
2. McKenna A, Hanna M, Banks E, Sivachenko A, Cibulskis K, et al. (2010) The Genome Analysis Toolkit: a MapReduce framework for analyzing next-generation DNA sequencing data. Genome research 20: 1297-1303.
3. Koboldt DC, Chen K, Wylie T, Larson DE, McLellan MD, et al. (2009) VarScan: variant detection in massively parallel sequencing of individual and pooled samples. Bioinformatics 25: 2283-2285.
4. Ng PC, Henikoff S (2003) SIFT: Predicting amino acid changes that affect protein function. Nucleic acids research 31: 3812-3814.
5. Robinson MD, McCarthy DJ, Smyth GK (2010) edgeR: a Bioconductor package for differential expression analysis of digital gene expression data. Bioinformatics 26: 139-140.
6. Ning Z, Cox AJ, Mullikin JC (2001) SSAHA: a fast search method for large DNA databases. Genome research 11: 1725-1729.
7. Robinson MD, Oshlack A (2010) A scaling normalization method for differential expression analysis of RNA-seq data. Genome biology 11: R25.
8. Xiao F, Zuo Z, Cai G, Kang S, Gao X, et al. (2009) miRecords: an integrated resource for microRNA-target interactions. Nucleic acids research 37: D105-110.
9. Hsu SD, Lin FM, Wu WY, Liang C, Huang WC, et al. (2011) miRTarBase: a database curates experimentally validated microRNA-target interactions. Nucleic acids research 39: D163-169.
10. Papadopoulos GL, Reczko M, Simossis VA, Sethupathy P, Hatzigeorgiou AG (2009) The database of experimentally supported targets: a functional update of TarBase. Nucleic acids research 37: D155-158.
11. Dweep H, Sticht C, Pandey P, Gretz N (2011) miRWalk--database: prediction of possible miRNA binding sites by "walking" the genes of three genomes. Journal of biomedical informatics 44: 839-847.
12. Garcia DM, Baek D, Shin C, Bell GW, Grimson A, et al. (2011) Weak seed-pairing stability and high target-site abundance decrease the proficiency of lsy-6 and other microRNAs. Nature structural & molecular biology 18: 1139-1146.
